# Thermal runaway-induced short-circuit arc in highly integrated lithium-ion battery systems: mechanisms, thresholds, and mitigation strategies

**DOI:** 10.1038/s44172-026-00657-w

**Published:** 2026-04-06

**Authors:** Zhenxing Yu, Chuan Chen, Pengfei Zhao, Yantao Qiao, Yu Cao, Binwei He, Zhimin Liu

**Affiliations:** Department of Power Batteries, Intelligent Vehicle Group, Li Auto Inc., Beijing, China

**Keywords:** Batteries, Chemical engineering

## Abstract

Thermal runaway-induced short-circuit arc in battery cell blocks or modules pose severe risks to electric vehicle safety; Despite this risk, however, their failure mechanisms, path and safety thresholds remain insufficiently understood. This study addresses these gaps by investigating short-circuit arc in commercial battery systems. Three short-circuit arc modes (case-to-case, case-to-busbar, and busbar-to-busbar) and corresponding failure paths were identified, with the case-to-case mode confirmed as the most hazardous. Experimental characterization reveals three phenomena during the case-to-case mode: weak Joule heating, intrinsic breakdown, and thermal breakdown with temperatures exceeding 1400 °C under 117 V and 7.2 mm, capable of triggering adjacent cell thermal runaway. A quadratic correlation between the critical breakdown voltage and electrode spacing was established, and a risk map was proposed. This study clarifies the mechanisms of thermal runaway-induced short-circuit arc, providing a quantitative safety design tool for highly integrated/high-voltage battery systems.

## Abstract (web summary)

Zhenxing Yu and colleagues report that thermal runaway in lithium-ion battery releases conductive ejecta, which induces three short-circuit arc modes in high-voltage systems (case-to-case arc being the most dangerous failure mode). They reveal a three-stage arc mechanism, establish a quadratic relationship between critical breakdown voltage and electrode spacing, and propose a risk map to guide safer design of highly integrated and ultra-fast charging battery systems.

## Introduction

Fire incidents in electric vehicles attributed to thermal runaway (TR) in power batteries have emerged as the most critical hazard compromising both user safety and industrial advancement^1^. Developing battery systems with enhanced thermal safety performance^2^ or even with No Thermal Propagation (NTP) ability has become an indispensable direction for nearly all battery suppliers and vehicle manufacturers. During cell TR, substantial heat, gas, and conductive ejecta are released, accompanied by component deformation. This heat can induce TR in adjacent cells, while conductive ejecta may bridge electrical components, triggering short-circuit, arc, and subsequent fires^3–5^. NTP technology refers to the use of insulation materials, thermal barriers, and exhaust components integrated within battery systems to prevent other cells from undergoing cascading TR and fire^6^. Owing to the high temperature, electrical conductivity, and unpredictable deposition, this solid ejecta poses a high risk of causing short circuit and arc in battery systems^7,8^. This failure mode has become the greatest technical challenge.

**Fig. 1:**
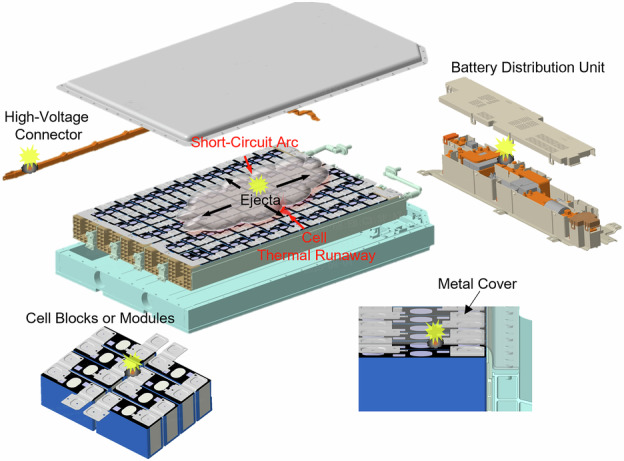
Common short-circuit arc forms are induced by cell thermal runaway in battery systems. Insets illustrate the different short-circuit forms.

There are already some common forms of short circuit and arc triggered by conductive ejecta, as shown in Fig. 1: short-circuit arc in the Battery Distribution Unit (BDU), short-circuit arc between high-voltage connectors, short-circuit arc between metal structural components, and short-circuit arc in the cell blocks or modules. Current commercial battery systems incorporate a range of insulation protection measures, such as composite material protective boxes for BDU, ceramic fiber insulation coatings, and mica material protective plates on the surface of high-voltage components.

Currently, a wealth of research has been reported on the high-voltage arcs of lithium-ion batteries^9–11^. Notably, short-circuit arcs induced by TR ejecta from cells have attracted research interest. The effects of the electrode gap, ejecta particle size and composition (primarily cathode material and copper fragments), and load resistance on short-circuit arc have been investigated^12–14^. It has been preliminarily revealed that there is a quadratic positive correlation between the critical breakdown voltage and spacing, whereas there is a negative correlation with the particle size. However, nearly all studies have relied on introducing one or more additional electrodes adjacent to the cells, which are not present in practical battery systems, to investigate short-circuit arc behaviors^15,16^. Therefore, the formation mechanism and path of high-voltage short-circuit arc caused by actual ejecta in practical battery systems are still unclear, and safety thresholds still require optimization.

This study addresses these gaps by investigating the high-voltage short-circuit arc failure mechanism and path in commercial Lithium-Nickel-Manganese-Cobalt-Oxide (NCM, Ni> 60 at. %) battery systems. Three short-circuit arc modes (case-to-case, case-to-busbar, and busbar-to-busbar) and corresponding failure paths were identified. By quantifying the critical thermal breakdown voltage across different electrode spacings in case-to-case mode, this study provides critical safety design guidelines for the development of highly integrated pack structures, such as Cell-to-Pack (CTP) and Cell-to-Chassis (CTC) systems, and ultrafast-charging lithium-ion battery systems (e.g., 5 C @ 800 V platform systems)^17^. These systems have more compact structures and higher platform voltages that need to meet the specified electrical clearance requirements^18,19^.

## Results and Discussion

### TR-induced short-circuit arc modes

**Fig. 2:**
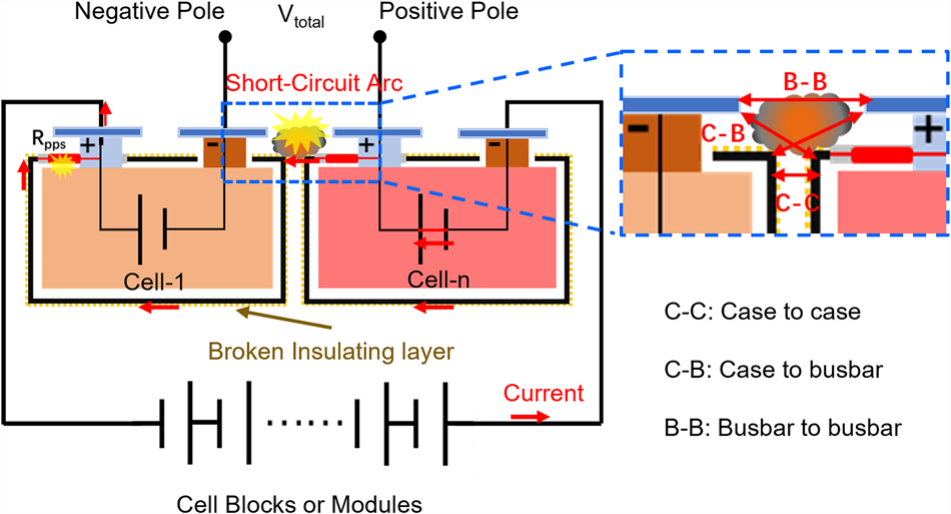
Equivalent circuit and failure modes of single cell thermal runaway-induced short-circuit arc on the cell blocks or modules. The inset illustrates three short-circuit failure modes: case-to-case, busbar-to-busbar and case-to-busbar.

Among all the failure forms shown in Fig. 1, the short-circuit arc on the cell blocks or modules poses the highest safety risk. In the circuit of a high-voltage battery system composed of multiple cells connected in series, there is a voltage difference between the different cells and their cases. The value of this voltage difference is the sum of the individual cell voltages between these two cells (or two short-circuit points). This voltage difference is the root cause of short-circuit and arc. Because the short-circuit arc with temperature far exceeding 1000 °C^20^ can easily melt the aluminum cases of cells, further causing leakage of the electrolyte. Additionally, this heat can also transfer into the interiors of cells through metal components, melting separators and triggering internal short circuits of the jelly roll (JR), ultimately leading to the TR of adjacent cells. This failure mode has become a key factor triggering thermal propagation in highly integrated battery systems. Based on the identified short-circuit paths and the involved short-circuit electrodes, the short-circuit arc on the cell blocks or modules is defined as three failure modes, as illustrated in Fig. 2.

1. Case-to-case (C-C): Conductive metal ejecta bridges the case of the TR cell and the adjacent cell, of which the insulation layers have been broken by the high temperature of cell TR.
2. Busbar-to-busbar (B-B): Ejecta connects the adjacent two exposed busbars or electrode terminals of the TR cell and adjacent cell.
3. Case-to-busbar (C-B): Ejecta connects the case and the adjacent busbar of the TR cell and adjacent cell.

From the equivalent circuit of the short-circuit arc on the cell blocks or modules, it can be understood that the short-circuit voltages for all three modes correspond to the total voltage of the series-connected cells in the affected circuit. When the conductive ejecta bridges the gap between the two cells, the circuit is connected, and a short-circuit current is generated. If the voltage exceeds the critical breakdown voltage, a severe arcing event may occur. In the C-C and C-B modes, the Polyphenylene Sulfide (PPS) insulator between the positive terminal and the cell case with a certain resistance, which could enhance the electrochemical corrosion under extreme conditions, may undergo thermal degradation or electrical breakdown due to heating. This introduces an additional TR risk to the adjacent cells. We propose that the C-C mode presents the most severe safety risk owing to its smaller electrode spacing (generally, 1–10 mm), higher total voltage, and proximity to the internal JRs of the cells. In contrast, the B-B mode exhibits a relatively lower risk than other two failure modes, characterized by a larger typical electrode spacing (generally, 10–25 mm between busbars) and a greater distance from the cell JR. Therefore, this study focused on investigating the failure mechanism and safety thresholds of the C-C mode using a commercial battery system and a custom-built device.

### Results of three short-circuit arc tests in battery system

**Fig. 3:**
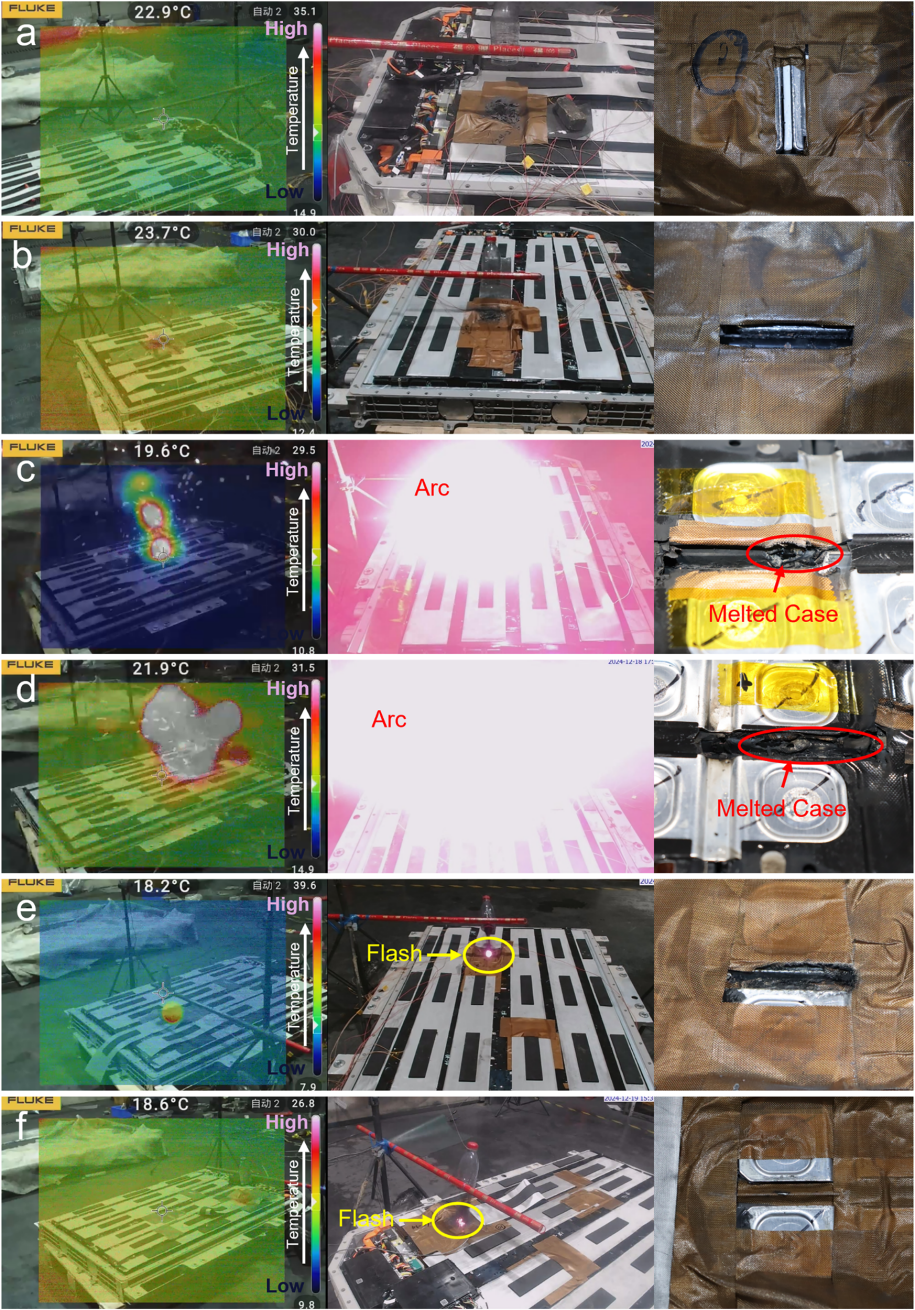
Infrared images, video snapshots, and post-test sample photographs from short-circuit arc tests in battery system. Case-to-case mode tests: (**a**) 7.1 V under 4.6 mm electrode spacing; (**b**) 53.1 V, (**c**) 116.9 V, and (**d**) 159.4 V under 7.2 mm electrode spacing; Case-to-busbar mode test: (**e**) 113.0 V under 11.0 mm; Busbar-to-busbar mode test: (**f**) 120.5 V under 11.5 mm.

To validate the three short-circuit arc modes above, a 42.8 kWh and 367.5 V @ 1/3 C commercial battery system with the 1P98S 117 Ah Lithium-Nickel-Manganese-Cobalt-Oxide (Ni> 60 at. %) -graphite cells was tested. Prior to testing, the original insulation protection of the battery system was removed to induce the short-circuit arc more easily. Simultaneously, to ensure safety after triggering an short-circuit arc, the open-circuit voltage (OCV) of the battery system was adjusted to 3.5 V to prevent TR of adjacent cells, thereby avoiding uncontrollable thermal propagation. Six tests were designed to cover all three short-circuit arc modes, with variations in the electrode spacing and voltage at different positions within the battery block. The results are shown in Fig. 3. In the C-C mode, when the short-circuit voltage was 7.06 V with 4.6 mm electrode spacing, there was no temperature increase in the infrared camera. When the voltage increased to 53.1 V, a slight temperature rise occurred at the short-circuit position, as shown in Fig. 3b. When the short-circuit voltage further increased to 116.9 V and 159.4 V, the severe short-circuit arc accompanied by a significant temperature rise broke the cases of cells within approximately one second. The disassembly results of the broken cells showed that the JR and other components inside also melted significantly, as shown in Supplementary Fig. 1. In the C-B mode test under 113.0 V with 11.0 mm electrode spacing, there were many obvious flashes at the short-circuit point, accompanied by a temperature rise in the infrared camera, but no severe short-circuit arc phenomenon, as shown in Fig. 3e. In the B-B test mode under 120.5 V with 11.5 mm electrode spacing, it had similar flashes and temperature increases to that of 113.0 V in the C-B mode. The corresponding videos are included in the supporting information as Supplementary Movies 1–5. Based on the above results, we conclude that the short-circuit arc in the C-C mode poses a greater safety hazard to adjacent cells than the other two modes.

**Fig. 4:**
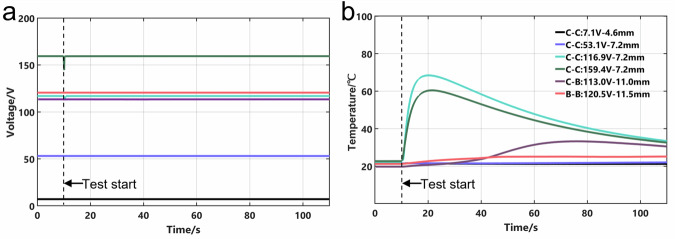
The data of short-circuit arc tests under different conditions in three failure modes in battery system. **a** Short-circuit voltage curves; (**b**) Temperature curves close to the short-circuit point.

Figure 4 presents the curves of short-circuit voltage and temperature of the busbar close to the short-circuit point. When the short-circuit voltage exceeded 116.9 V, once the ejecta was added to the short-circuit point at the 10^th^ second, there was a rapid temperature rise and voltage drop simultaneously owing to the short-circuit arc. In the 159.4 V test, the voltage decreased by approximately 15 V, with the busbar reaching a maximum temperature exceeding 60 °C. Notably, the internal temperature of the short-circuit point can reach over a thousand degrees^21^, which can easily melt the aluminum case of the cells and cause electrolyte leakage, as shown in Fig. 3c, d. However, the ejecta at the short-circuit point was rapidly expelled during the arc-induced explosion, resulting in an open circuit and the temperature returning to room temperature. Two key factors explain why the 116.9 V and 159.4 V tests (7.2 mm spacing) did not induce cell thermal runaway: On the one hand, the OCV of battery system was only 3.5 V, a relatively safe level. On the other hand, the short-circuit was terminated when the conductive ejecta was dispersed by the arc-induced explosion. The experimental results demonstrating cell TR caused by the short-circuit arc are shown in Supplementary Figs. 2 and 3. Collectively, these findings confirm that the C-C mode poses the highest safety risk, with short-circuit arc occurrence directly correlated with voltage and electrode spacing.

### Results of C-C mode short-circuit arc tests on cells

**Fig. 5:**
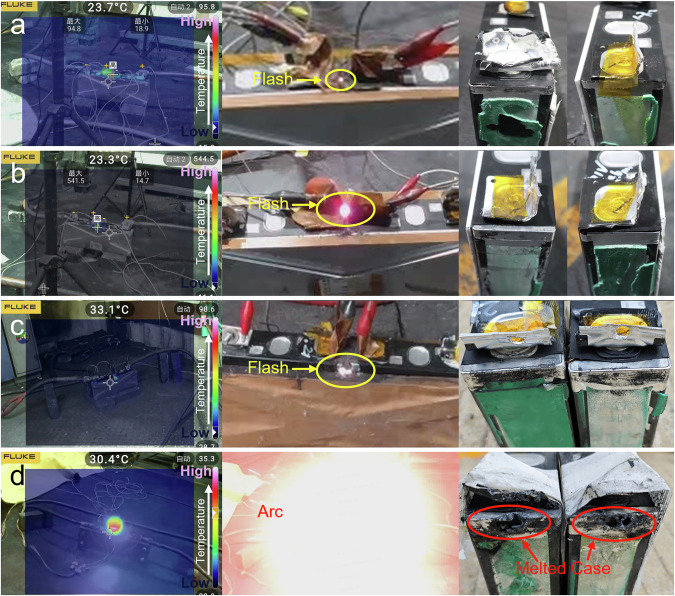
Infrared images, video snapshots, and post-test sample photographs from case-to-case mode tests on cells. **(a**–**d**) correspond to short-circuit voltages of 55.8 V, 91.2 V, 103.0 V, and 117.0 V, respectively, with a fixed electrode spacing of 7.2 mm.

To investigate the short-circuit arc mechanism and safety threshold of the C-C mode short-circuit arc, further experiments were conducted using the same commercial 117 Ah prismatic NCM cells. A custom-built experimental device comprising a voltage boosting, short-circuit tester, and fixture that can adjust the spacing of the two cells was established. Two cells were fixed with a lateral electrode spacing of 7.2 mm using a fixture. The voltage boosting module can provide an adjustable high-voltage to the cells to simulate the actual high voltage condition in the battery system. The Short-Circuit tester is used as a switch to control the on/off of the circuit and collect the short-circuit current through the shunt. High-speed data acquisition was used to record the temperature and voltage during the testing process. The observations are shown in Fig. 5. Infrared imaging confirmed significant temperature increases at the short-circuit point in all tests upon high-voltage application. Within the voltage range of 55.8 to 103 V (Fig. 5a–c), numerous flashes accompanied by crackling sounds were observed on the surface of the ejecta. The flash brightness intensified progressively with increasing short-circuit voltage, but the discharge process ceased within one minute, and the cell cases retained structural integrity. At 117 V (Fig. 5d), the short-circuit point emitted an intense arc light followed by an explosion. The corresponding videos are included in the supporting information as Supplementary Movies 6–9. The cell cases adjacent to the short-circuit point exhibited significant melting and electrolyte leakage.

**Fig. 6:**
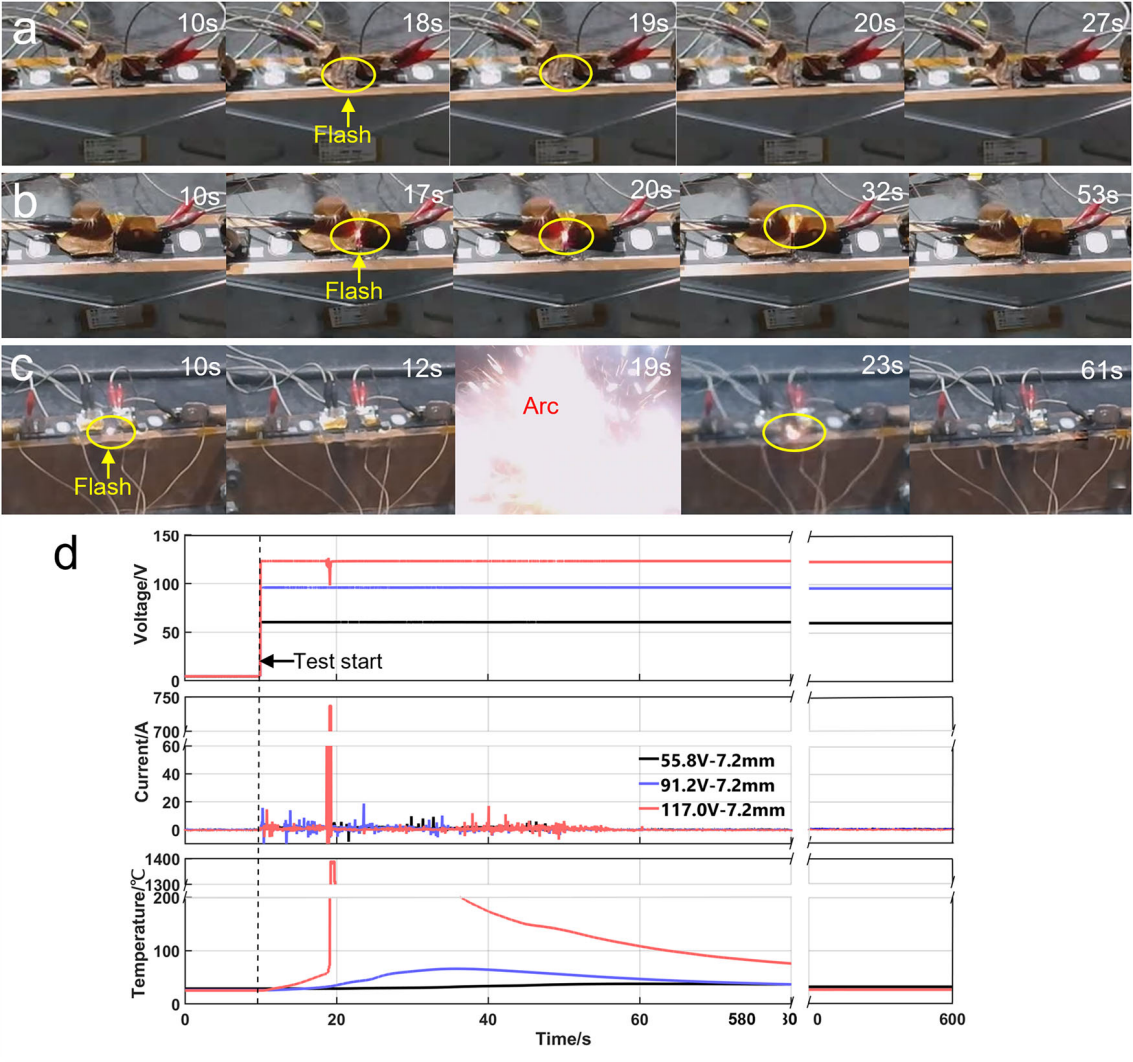
Short-circuit arc phenomena and associated data of case-to-case mode test on cells. (**a**–**c**) are short-circuit arc phenomena under 55.8 V, 91.2 V and 117.0 V, respectively, with a fixed electrode spacing of 7.2 mm; (**d**) Corresponding curves of short-circuit voltage, current and temperature during tests.

Figure 6 shows the C-C mode short-circuit arc behavior and curves of the voltage, current, and temperature of above experiments. Three distinct phenomena were observed during the test. First, when a 55.8 V voltage was applied to the cells at the 10^th^ second, some weak flashes appeared on the ejecta surface at the 18^th^ second (Fig. 6a), and there was a weak and unstable current, accompanied by a slow temperature rise that stabilized until the end of the test (Fig. 6d). Then, under 91.2 V, multiple high-amplitude pulse currents were recorded within 43 s, with a maximum current of nearly 20 A. These pulses corresponded to faint short-circuit flashes on the ejecta surface and a temperature increase to approximately 60 °C (Fig. 6b). The temperature subsequently dropped to approximately 40 °C after ~30 s and remained stable thereafter. The voltages in all these tests remained stable, with no significant fluctuations. Finally, when the voltage reached 117.0 V, the short-circuit point emitted an intense arc light with an explosive sound at the 19^th^ second, as shown in Fig. 6c. This triggered a rapid temperature surge to ~1400 °C, a 25 V voltage drop, and an instantaneous peak current of approximately 740 A, as shown in Fig. 6d. The results above indicate that a severe short-circuit arc poses a critical safety hazard to battery cells.

**Fig. 7:**
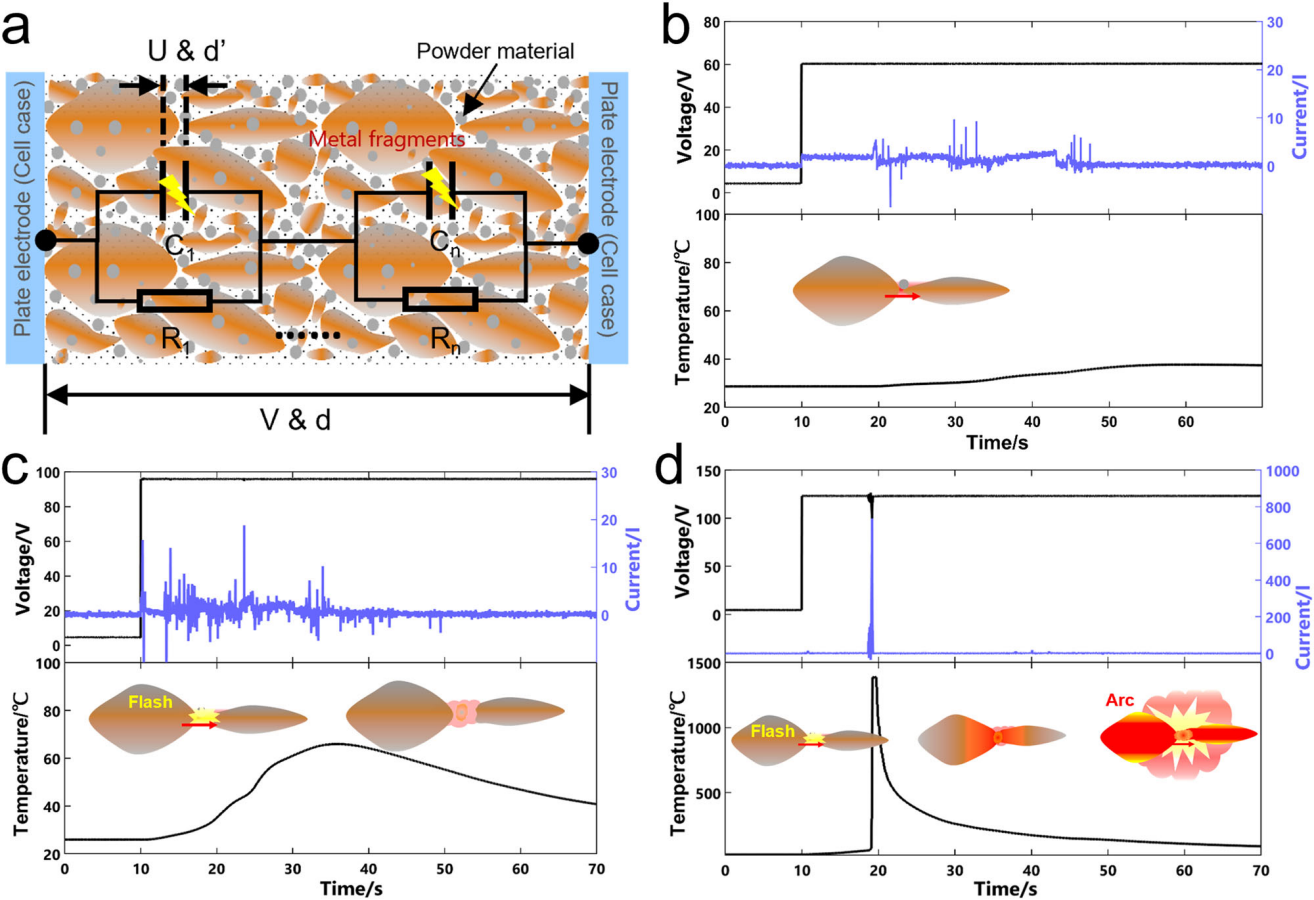
Equivalent circuit model and forming mechanism of three different modes. **a** Equivalent circuit model of conductive network in ejecta; (**b**–**d**) represent the forming mechanisms of Joule heat, intrinsic breakdown, and thermal breakdown, respectively, under 55.6 V, 91.2 V, and 117.0 V.

An equivalent circuit model was established, as shown in Fig. 7a. This model consists of two parallel electrodes (representing the cases of two cells) with ejecta sandwiched between them. In this model, it is assumed that the substances and gaps in the ejecta are uniformly distributed in the gap. Within a local unit of the ejecta, conductive metal fragments form interconnected conductive networks, and the interface of the gap accumulates a large amount of electric charge, forming a capacitance effect. Each local unit is modeled as a parallel combination of a conductive network contact resistor R and a gap capacitor C. The overall equivalent circuit is constructed by connecting multiple such local units in series. Joule heat, intrinsic breakdown, and thermal breakdown can occur in this model under different conditions, which can explain the three different phenomena in the above experiments. Contact resistance generates Joule heat during short-circuit current flow; the microscopic gap, which represents the average gap value (d’) of metal fragments rather than the total electrode spacing (d), is very small. And a large number of sharp tips on gaps interface significantly reduce the critical breakdown voltage, making it easier for high voltage to break through the air between metal fragments, which is known as intrinsic breakdown^22–25^. When the voltage was low, such as at 55.6 V, a continuous weak short-circuit current flowed through the contact points of the conductive network, generating mild Joule heat. Simultaneously, sporadic intrinsic breakdown induced faint flashes on the ejecta surface. These combined effects resulted in a stable temperature profile, as shown in Fig. 7b. When the voltage increased to 91.2 V, Increased voltage intensified the intrinsic breakdown within the ejecta, causing the melting of the connected fragment tips. This melting expands the microscopic gaps, disrupting the conductive network and interrupting the circuit formation. Consequently, the generation of Joule heat and intrinsic breakdown heat was attenuated, leading to a subsequent temperature decrease, as shown in Fig. 7c. However, when the voltage was increased to 117.0 V, the heat accumulated from the short circuit and strong intrinsic breakdown caused a large amount of ejecta to melt, which significantly decreased the contact resistance of the conductive networks and generated a higher short-circuit current. This large current further triggered a sharp increase in temperature, arc light, and explosion in approximately 10 s, which is called thermal breakdown, as shown in Fig. 7d. Given the extremely high heat release rate and short duration of the thermal breakdown, this process is assumed to follow an adiabatic temperature rise model with no heat dissipation. Specifically, the powder material in the ejecta is assumed to consist of 8.1 g of NCM cathode material, while the metal fragments comprise 5 g of copper foil, both of which undergo temperature elevation during thermal breakdown. The overall temperature increase was calculated as follows:

1 $$V* I* {dt}=\left({C}_{{{{\rm{NCM}}}}}* {m}_{{{{\rm{NCM}}}}}+{C}_{{{{\rm{Cu}}}}}* {m}_{{{{\rm{Cu}}}}}\right)* {dT}$$

Therefore, the temperature increase (*dT)* of the ejecta at the short-circuit point is

2 $${dT}=\frac{V* I* {dt}}{({C}_{{{{\rm{NCM}}}}}* {m}_{{{{\rm{NCM}}}}}+{C}_{{{{\rm{Cu}}}}}* {m}_{{{{\rm{Cu}}}}})}$$

Where *V* is the short-circuit voltage, *I* is the average short-circuit current (approximately 124 A for 550 ms), *dt* is the short-circuit time, *C*_NCM_ and *C*_Cu_ are the specific heat capacities of the active material and copper, and *m*_NCM_ and *m*_Cu_ are the masses of the active material and copper at the short-circuit point. The temperature rises *dT* estimated under 117.0 V @ 7.2 mm was near 1000 °C. However, in practical scenarios, thermal breakdown is confined to a localized fraction of the ejecta, rather than the entire mass. The actual temperature at the short-circuit point was higher than 1000 °C. The temperature recorded at the 19^th^ second reached approximately 1400 °C under 117.0 V @ 7.2 mm (Fig. 7d). Therefore, the arc induced by the thermal breakdown acts as the primary triggering factor for safety hazards in adjacent cells and serves as a pivotal driver of thermal propagation.

**Fig. 8:**
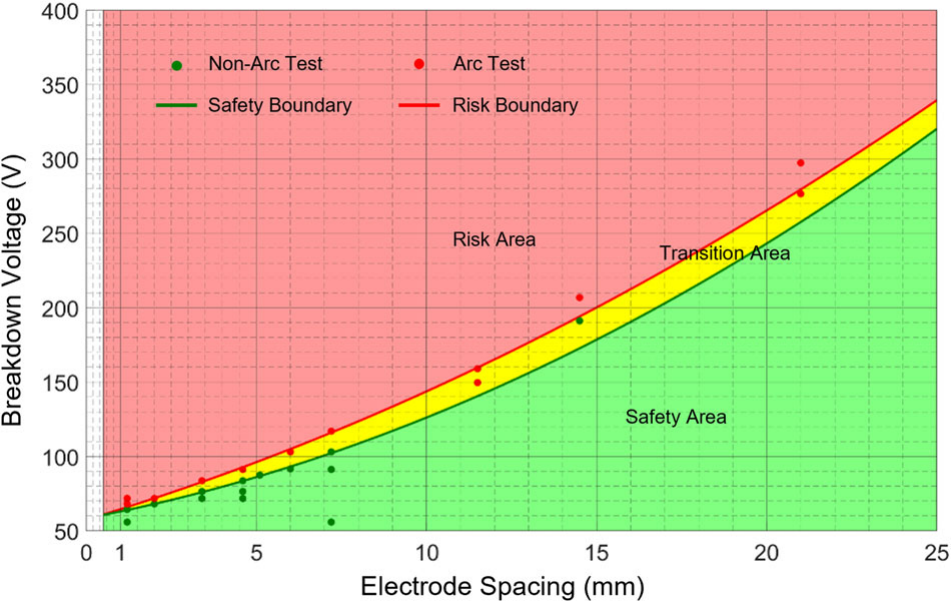
Risk map of thermal breakdown voltage under different electrode spacings in the case-to-case mode.

Finally, a systematic investigation was conducted to characterize the critical thermal breakdown voltages across a practical electrode spacing range of 1.2–21 mm, and the complete dataset is presented in Supplementary Table 1 (The raw data is provided in Supplementary Data 1). Supplementary Movies 10 and 11 demonstrate that cells thermal runaway were induced by short-circuit arc under 71.7 V @ 1.2 mm and 149 V @ 11.5 mm. The maximum non-arc and minimum arc-initiation voltages were plotted with the corresponding electrode spacing and fitted to obtain the risk map of the thermal breakdown arc, as shown in Fig. 8. The green line fitting from the maximum non-arc voltage represents the safety voltage boundary. The green area below the green curve is the Safe Area. The red line fitted from the minimum arc initiation voltages by a quadratic polynomial represents the risk voltage boundary^12,14^. It is expressed as Eq. (3):

3 $${V}_{{{{\rm{risk}}}}}=0.177{d}^{2}+6.858d+57.411$$

where *V*_risk_ is the thermal breakdown critical voltage (V), and *d* is the electrode spacing (mm). The red area above the risk boundary curve is the Risk Area. Once the ejecta falls into the gap with the voltage and corresponding electrode spacing in this area, it will cause a thermal breakdown arc, increasing the risk of thermal runaway of adjacent cells. The yellow area between the red and green curves is the Transition Area, in which there is a certain probability of thermal breakdown. The boundary of these areas are affected by the composition, size, mass and temperature of the ejecta fallen on the short-circuit point^26,27^. To date, no precise physical model has been established to describe the short-circuit thermal breakdown mechanism induced by a complex multi-phase medium. The critical breakdown voltages at different electrode spacings were primarily derived from experiments, which underscores the significance of this study.

## Conclusion

This study clarifies the short-circuit arc failure mechanism on the cell blocks or modules, which is a critical yet insufficiently explored hazard in thermal propagation-related EV safety research. Three dominant short-circuit arc modes, namely case-to-case (C-C), case-to-busbar (C-B), and busbar-to-busbar (B-B), and their corresponding failure paths were identified, with the C-C mode emerging as the primary driver of cascading thermal propagation owing to its proximity to cell JR, smaller electrode spacing, and higher voltage exposure. This study reveals three sequential phenomena in the C-C mode: weak Joule heating, intrinsic breakdown in microscopic gaps of the conductive ejecta, and thermal breakdown caused by the rapid accumulation of Joule and intrinsic breakdown heat. The failure mechanism of thermal breakdown, which triggers short-circuit arc and subsequently lead to the thermal propagation of the battery system, was proposed. The temperature of the thermal breakdown point reached approximately 1400 °C under 117.0 V with an electrode spacing of 7.2 mm.

A key insight lies in the quantified quadratic correlation between the critical thermal breakdown voltage and electrode spacing, derived from experiments on practical battery systems and cell TR ejecta, and the proposed short-circuit arc risk map. This correlation and map provide a quantitative tool for the design of electrical clearance in highly integrated/ultrafast-charging battery systems. The failure mechanism establishes a theoretical foundation for formulating mitigation strategies against cell TR-induced short-circuit arc. Specific recommendations include: rational optimization of the battery system high-voltage connection topology to minimize voltage differences between adjacent electrical components, adoption of battery pack designs that physically isolate TR venting channels from electrical compartments, application of high-temperature-resistant insulating layers to electrical components adjacent to blocks or modules (e.g., cell terminals and busbars), and employment of safety-optimized cells with electrically insulated cases.

At present, research on short-circuit arc in complex multiphase media still faces substantial challenges, including the accurate acquisition of voltage, current, and temperature signals during arcing tests, as well as the theoretical underpinnings required to elucidate thermal breakdown phenomena. These challenges necessitate sustained efforts to be resolved.

## Methods

### Experimental Materials

The commercial prismatic NCM (Ni > 60 at. %) cell with electrically positive-connected case have been tested. According to the manufacturer’s specifications, the cell-rated capacity and voltage were 117 Ah @ 1/3 C and 3.75 V, respectively. It should be particularly noted that the case of this cell is connected to the positive terminal through Polyphenylene sulfide (PPS). This design is a common method for protecting the cell case from electrochemical corrosion under extreme conditions. Further information is provided in Supplementary Table 2.

A CTP battery system with the same 117 Ah prismatic NCM cells was also used for high-voltage short-circuit arc testing. Its rated energy and voltage are 42.8 kWh and 367.5 V @ 1/3 C. The 1P98S cell block of this system is composed of two 1P19S and three 1P20S modules.

### Preparation of ejecta from cell TR

The ejecta was obtained through cell nail penetration experiments. The cells were charged to 100 % State of Charge (SOC) with 1/3 C at 25 °C. A steel needle with a diameter of 3 mm was used to puncture the large-surface center of the cell at a speed of 1 mm·s^−1^ until thermal runaway occurred. All ejecta, including metal fragments (mainly copper current collector fragments and a small amount of molten aluminum) and powder material, were collected after the experiment. They were separated using a 100-mesh sieve and weighed. The results of the three parallel experiments are presented in Supplementary Table 3. The average weight-loss rate was 50 wt. %, and the mass ratio of powder material and metal fragments in ejecta is 1.62. As observed from the images and Energy Dispersive Spectroscopy (EDS) results in Supplementary Fig. 4, the powder materials mainly contain Ni, Co, Mn, Al, C, and F. These elements originated from the NCM cathode, the molten Al current collector, graphite of anode, and lithium salts in the electrolyte. In contrast, the Cu current collector from the anode, which has a high melting point, remained as metal fragments with sizes ranging from several to tens of millimeters. Therefore, the Cu element was not detected by EDS. To simulate the worst-case scenario, an excessive amount of mixture (13.1 g) was added to the gap between the two cells. The mass ratio of the powder to metal fragments in the ejecta was also 1.62, which was the same as that in the actual situation.

### Short-circuit arc tests in battery system

**Fig. 9:**
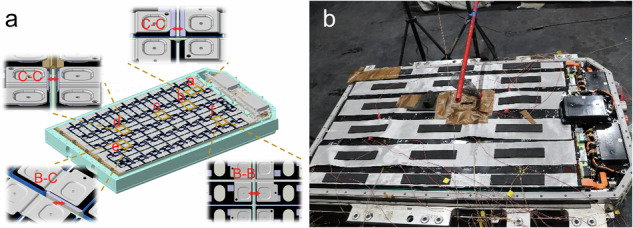
Custom-built short-circuit arc test device for the battery system. **a** Experimental positions; (**b**) photo of the experimental battery system.

To trigger the short-circuit arc in the battery system, the original protective designs must be removed. For instance, the insulating layers and rubbers on the shoulders of adjacent cells should be dismantled. Meanwhile, to ensure absolute experimental safety, the SOC of battery system was adjusted to 15 %, in which the OCV of the cell was 3.5 V. The adjacent cells with target voltages in the system were selected for the test, as shown in Fig. 9. To ensure that only one short-circuit mode occurred per experiment, the test positions of the battery system were protected using Teflon insulating tape. For the C-C mode, the insulating layers on the shoulders of two adjacent cells (with a target voltage difference) were damaged to expose the underlying metal cases, which served as electrodes for the high-voltage short-circuit arc. All other nearby electrical components, especially the busbars, were fully covered with Teflon insulating tape for insulation protection. Using the same insulation method, the C-B mode test positions were configured by damaging only the shoulder insulation layer of one cell and exposing the adjacent busbar of another cell. For the B-B mode, the test positions were designed to fully protect the cell cases while exposing only the busbars.

The ejecta was placed in the gap of the short-circuit point using the inverted container method. A container loaded with a preset amount of ejecta was positioned above the short-circuit point, and a rope fastened to the bottom of the container was routed outside the laboratory. Upon test initiation, the rope was pulled to rotate the container, causing the ejecta to fall onto the short-circuit point and triggering an short-circuit arc. The total voltage and temperature of the busbars close to the short-circuit point were recorded using high-speed data acquisition with a sampling frequency of 100 Hz. The experimental phenomena and temperature distributions were also recorded.

### Short-circuit arc tests on cells

**Fig. 10:**
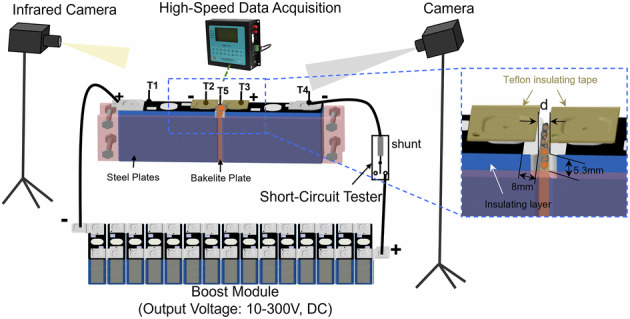
Schematic diagram and parameters of the custom-built short-circuit arc test device for the cells. The inset shows electrode dimensions and insulation protection schemes at short-circuit points.

A schematic of the of custom-built short-circuit arc test device is shown in Fig. 10. Two 100 % SOC cells were arranged side by side with the required spacing (d). Bakelite was filled in the gap between the two cells, spanning from 5.3 mm below the cell shoulder to the cell bottom, and the assembly was finally secured using steel plates. To simulate the insulation layer degradation of actual cells during TR, the insulation layer of two cells with a width of 8 mm at the top and a height of 5.3 mm on the side were removed. A boost module was employed to supply a high voltage to the circuit, and the output voltage was regulated by adjusting the number of series-connected cells in the circuit. A Short-Circuit tester (SC-DL204PC-MADJ (1-5mR)) with a 0.015 mΩ shunt was used as a switch to control the on/off of the circuit and collect the current within the range of 0–5000 A with a sampling frequency of 100 Hz. Meanwhile, a high-speed data acquisition device (Ganter, voltage sampling range: −1200 V–+1200 V, error: ± 0.03% FS; Temperature sampling range of K-type thermocouple: −100 °C–+ 1500 °C, error: ± 2 °C) was used to collect the short-circuit voltages and the temperatures close to the short-circuit point with a sampling frequency of 100 Hz.

The ejecta was placed in the gap between the two cells prior to testing. Then, the high-voltage circuit is connected by controlling the short-circuit tester to start. The test was terminated when the short-circuit point temperature returned to room temperature or remained stable, and the time reached 10 min. Throughout the test, the experimental phenomena and temperature distribution were recorded using cameras and infrared cameras (TI480PRO, temperature sampling range: −20– + 500 °C).

## Supplementary information

Supplementary Information

Description of Additional Supplementary Files

Supplementary Movie 1

Supplementary Movie 2

Supplementary Movie 3

Supplementary Movie 4

Supplementary Movie 5

Supplementary Movie 6

Supplementary Movie 7

Supplementary Movie 8

Supplementary Movie 9

Supplementary Movie 10

Supplementary Movie 11

Supplementary Data 1

## Data Availability

The data that support the findings of this study are available from the corresponding author upon reasonable request.
