# Supplementary material for: Thermal runaway-induced short-circuit arc in highly integrated lithium-ion battery systems: mechanisms, thresholds, and mitigation strategies
Source: Commun Eng. 2026 Apr 6;5:101. doi: 10.1038/s44172-026-00657-w (PMC13234359; doi:10.1038/s44172-026-00657-w)
Supplement: Supplementary file 1 — Supplementary Information [file 44172_2026_657_MOESM1_ESM.pdf]

Supplementary Information

Thermal Runaway-induced Short-Circuit Arc in Highly Integrated lithium-ion Battery Systems: Mechanisms, Thresholds, and Mitigation Strategies

Zhenxing Yu, Chuan Chen, Pengfei Zhao, Yantao Qiao, Yu Cao, Binwei He, Zhimin Liu*

Department of Power Batteries, Intelligent Vehicle Group, Li Auto Inc. Beijing, China. 101399

* Corresponding author

E-mail: liuzhimin@lixiang.com

Supplementary Notes 1

The disassembly results of the cell experienced thermal breakdown show that the Mylar and separator of jelly roll have significantly melted due to heat from thermal breakdown, posing a high thermal runaway risk.

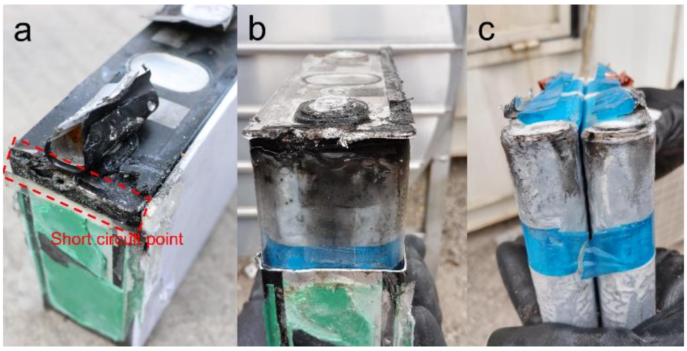

Supplementary Figure 1. The disassembly pictures of cells after case-to-case short-circuit arc test in the battery system with 116.9 V voltage and 7.2 mm electrode spacing. a Cell case; b Mylar layer; c Jelly roll.

Supplementary Notes 2

The cells experienced thermal runaway triggered by high voltage short-circuit arc under case-to-case mode with 71.7 V voltage and 1.2 mm electrode spacing (Supplementary Figure 2) and 149.6 V voltage and 11.5 mm electrode spacing (Supplementary Figure 3).

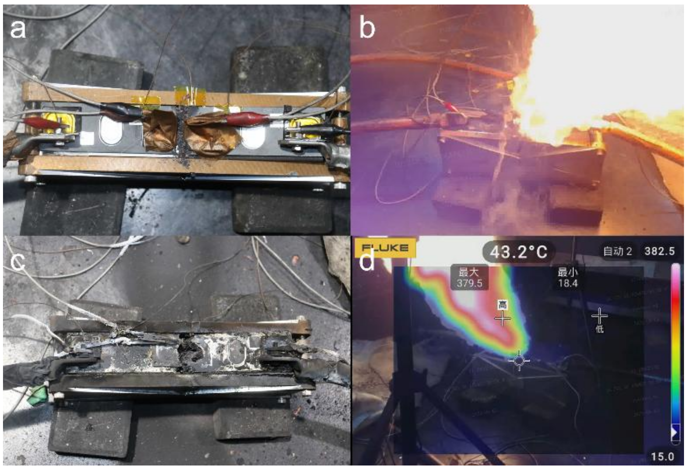

Supplementary Figure 2. The images of cells case-to-case short-circuit mode arc test with 71.7 V voltage and 1.2 mm electrode spacing. a and c are the sample pictures before and after the test respectively; b and d are the screenshot of the video and infrared video.

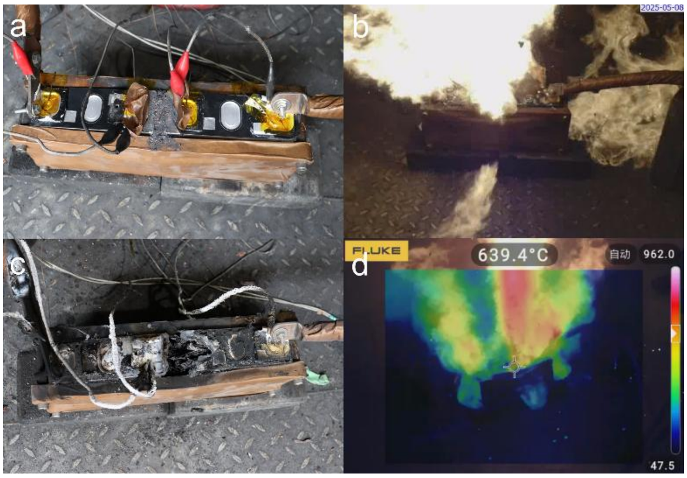

Supplementary Figure 3. The images of cells case-to-case mode short-circuit arc test with 149.6 V voltage and 11.5 mm electrode spacing. a and c are the sample pictures before and after the test respectively; b and d are the screenshot of the video and infrared video.

Supplementary Table 1. The results of case-to-case mode short-circuit arc test under different voltages and electrode spacings.

No.	Electrode spacing /mm	Voltage /V	V, T and I Curves	Samples after test	Infrared Image	Result
1	1.2	55.8	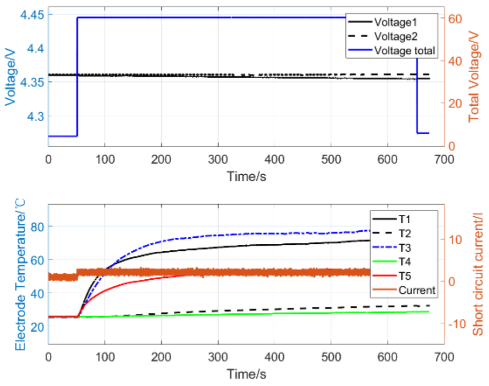	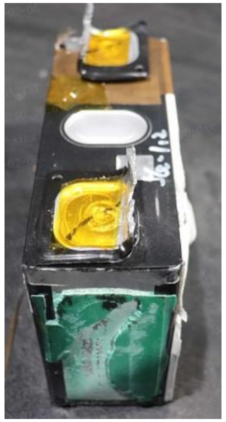	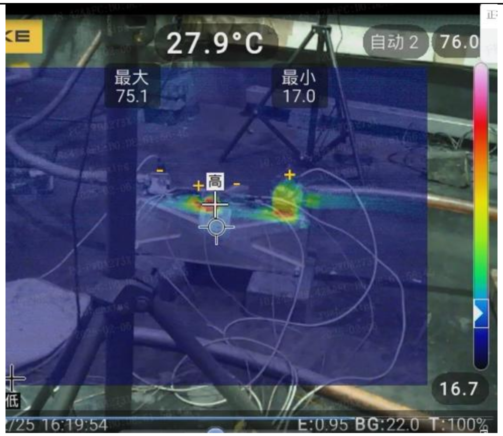	Flashes

2	1.2	63.7	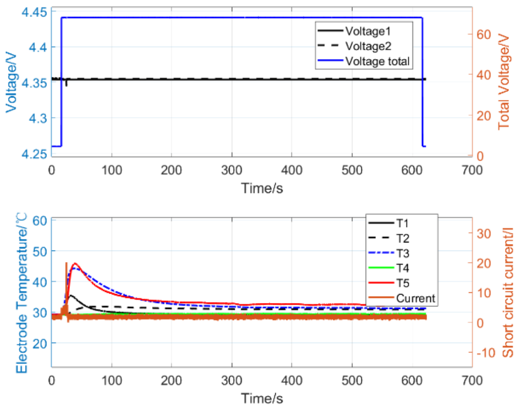	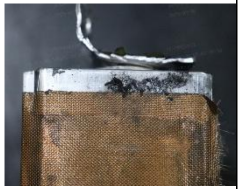	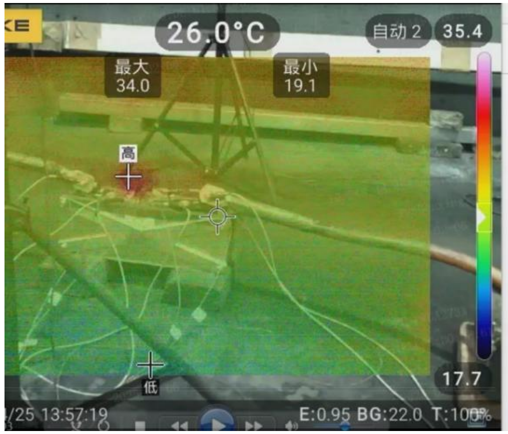	Flashes
3	1.2	68	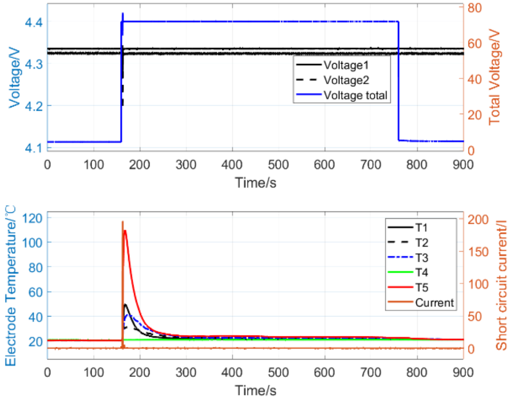	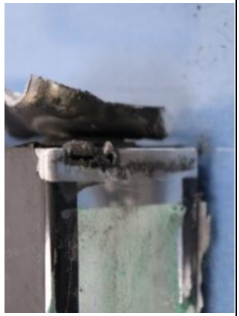	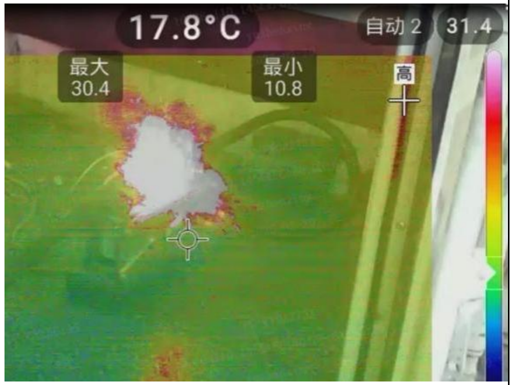	Arc

<table>
<tr>
<td></td><td></td><td></td><td></td><td></td><td></td><td></td>
</tr>
<tr>
<td>4</td>
<td>1.2</td>
<td>71.7</td>
<td>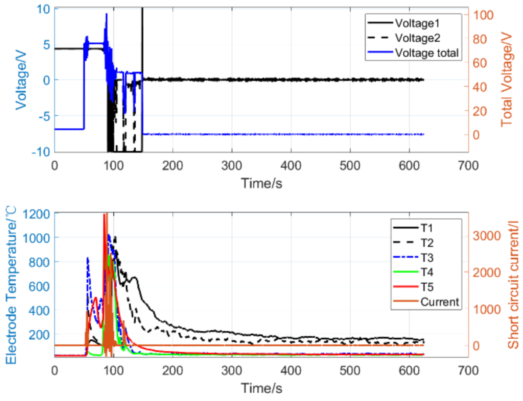
</td>
<td>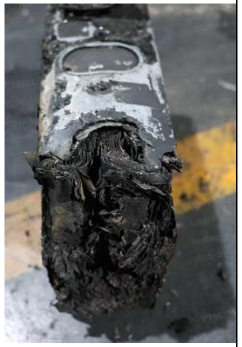</td>
<td>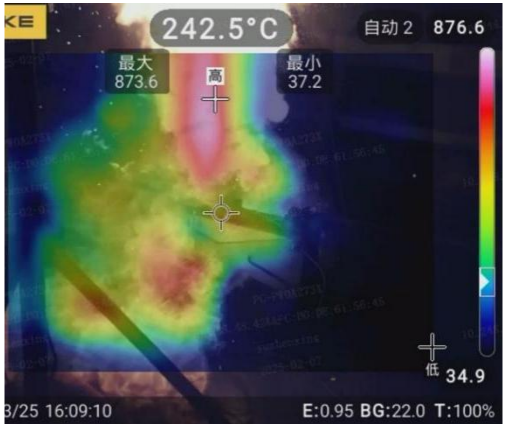
</td>
<td>Arc
&TR</td>
</tr>
<tr>
<td>5</td>
<td>2.0</td>
<td>68</td>
<td>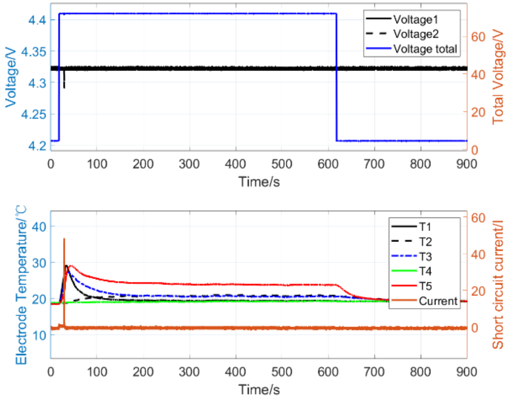
</td>
<td>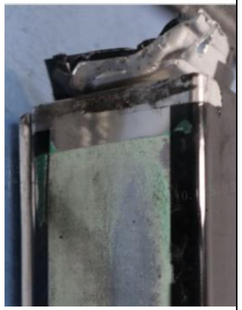</td>
<td>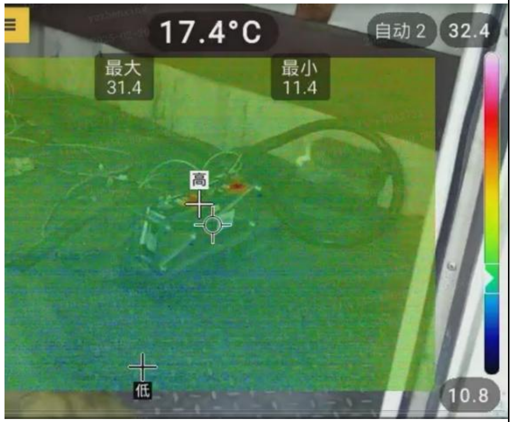
</td>
<td>Flashes</td>
</tr>
</table>

6	2.0	71.7	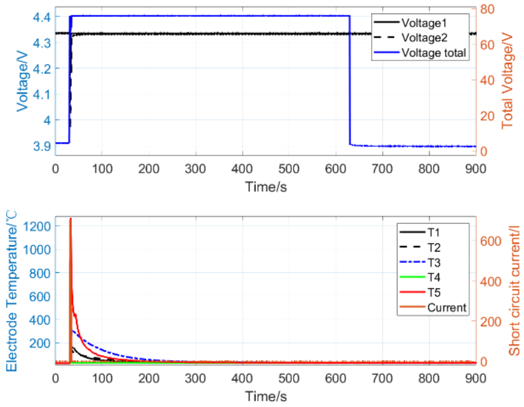	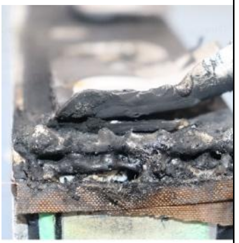	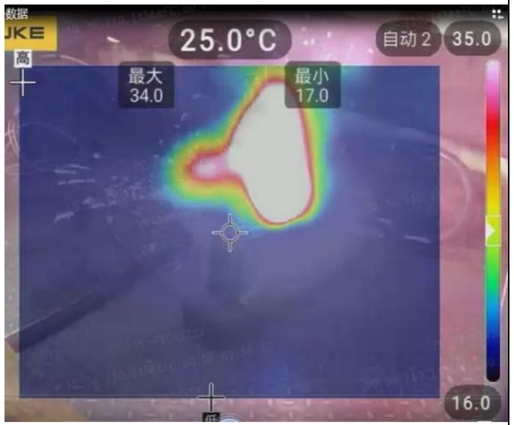	Arc
7	3.4	71.7	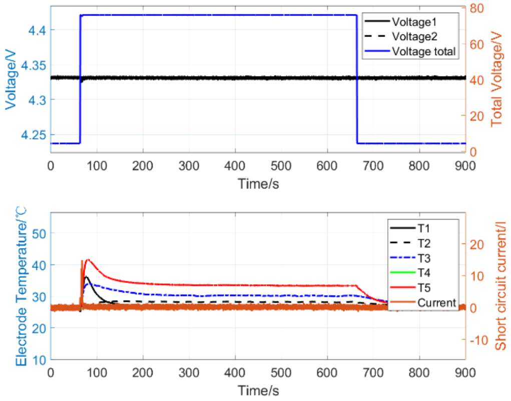	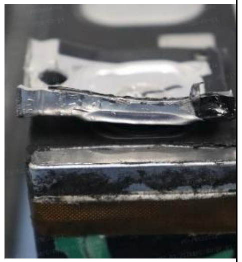	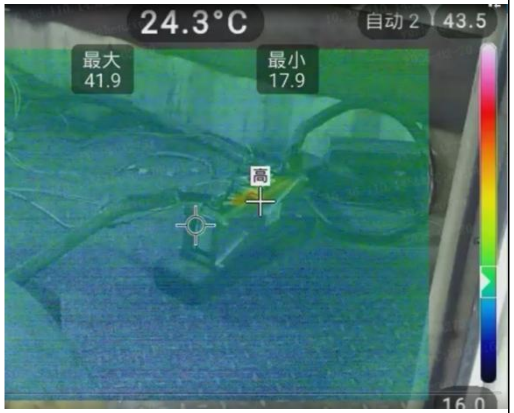	Flashes

8	3.4	76.4	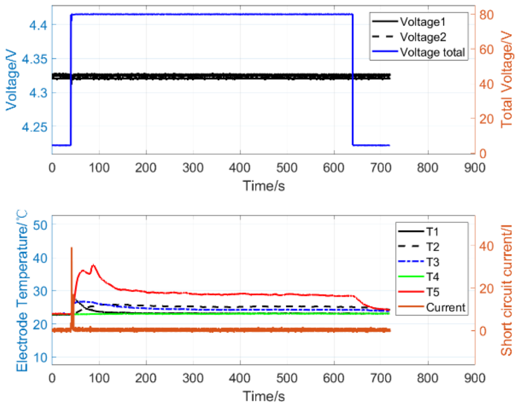	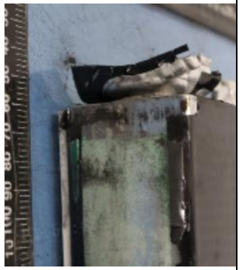	/	Flashes
9	3.4	83.6	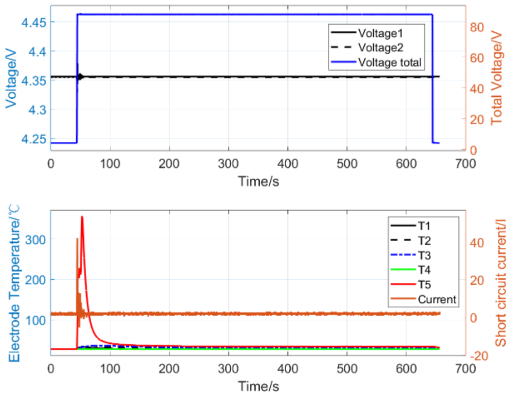	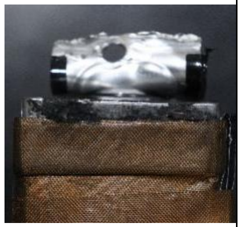	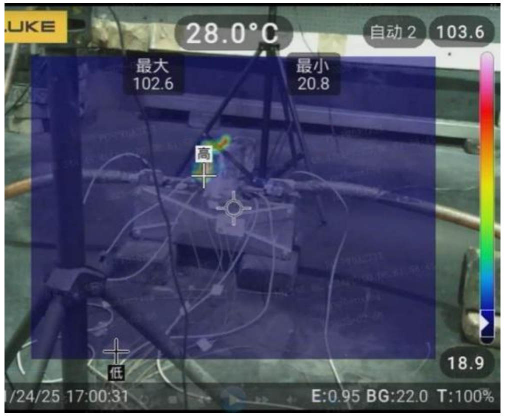	Flashes

<table>
<tr><td></td><td></td><td></td><td></td><td></td><td></td><td></td></tr>
<tr><td>10</td><td>4.6</td><td>71.7</td><td>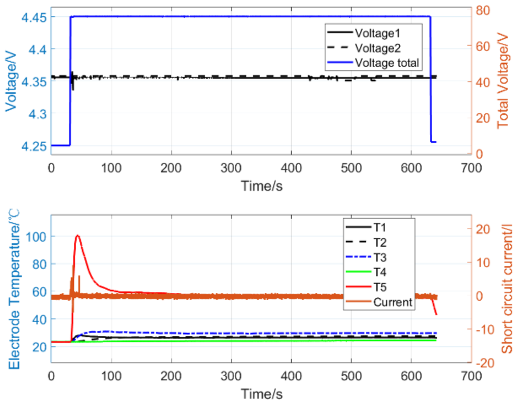
</td><td>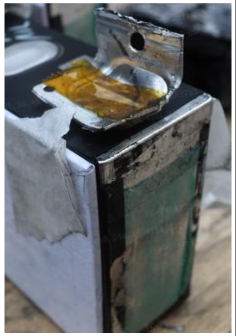</td><td>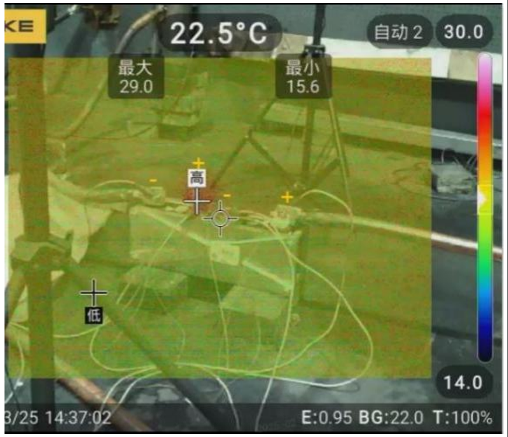
</td><td>Flashes</td></tr>
<tr><td>11</td><td>4.6</td><td>76.4</td><td>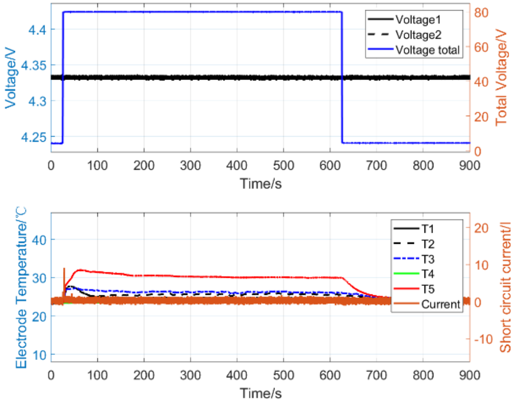
</td><td>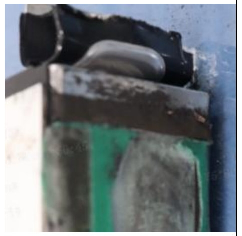</td><td>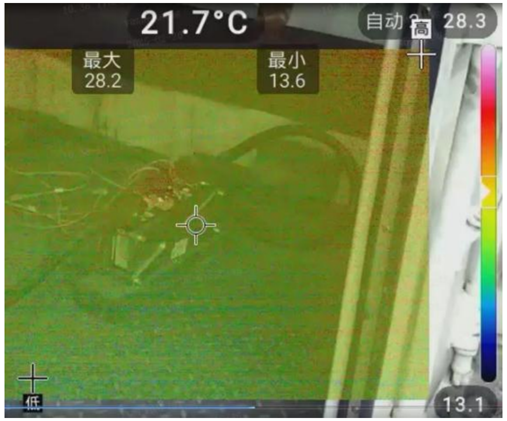
</td><td>Flashes</td></tr>
</table>

12	4.6	83.6	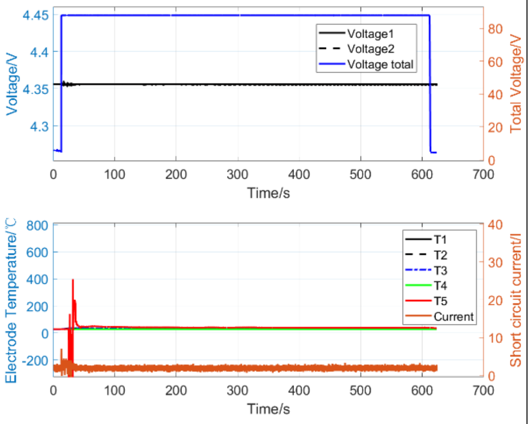	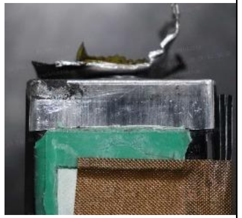	/	Flashes
13	4.6	91.2	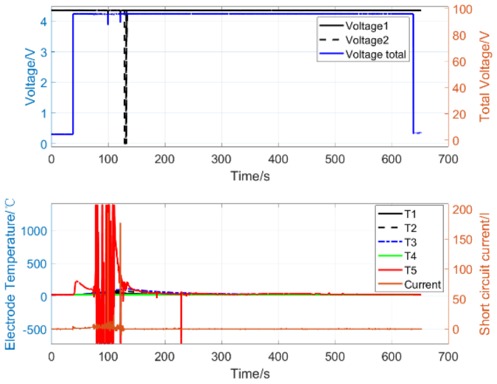	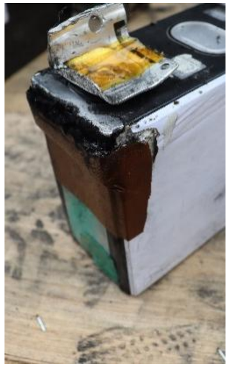	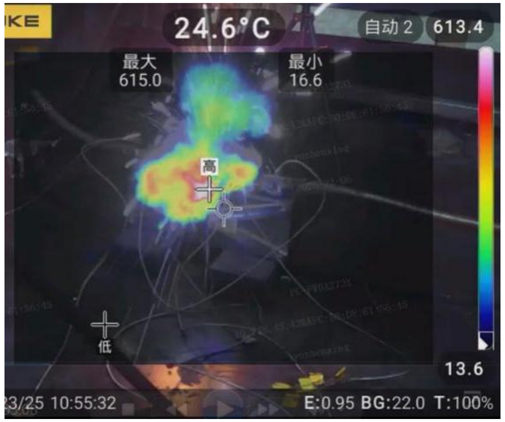	Arc

14	5.1	87.4	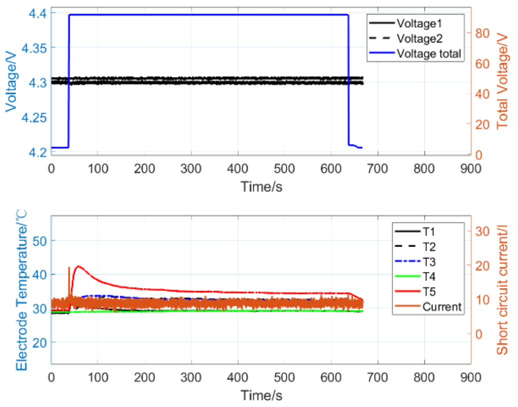	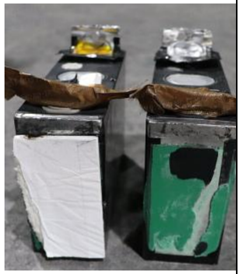	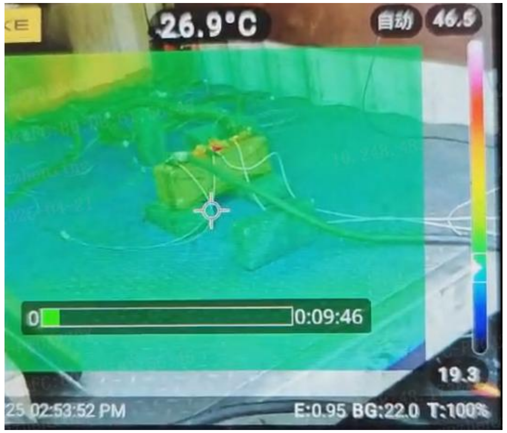	Flashes
15	6.0	91.2	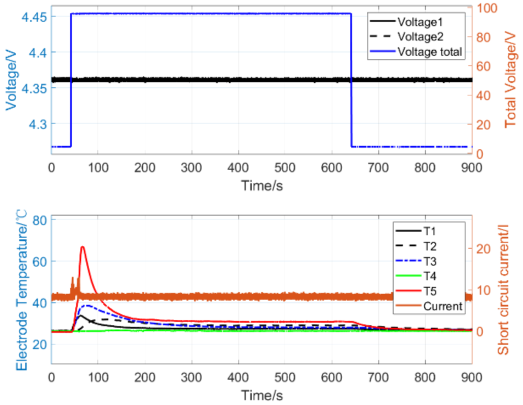	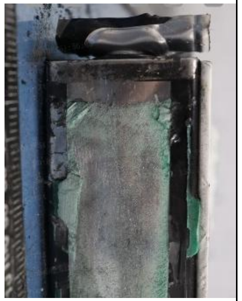	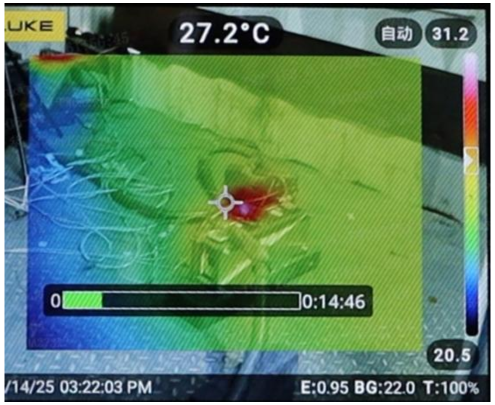	Flashes

<table>
<tr><td>16</td><td>6.0</td><td>103</td><td>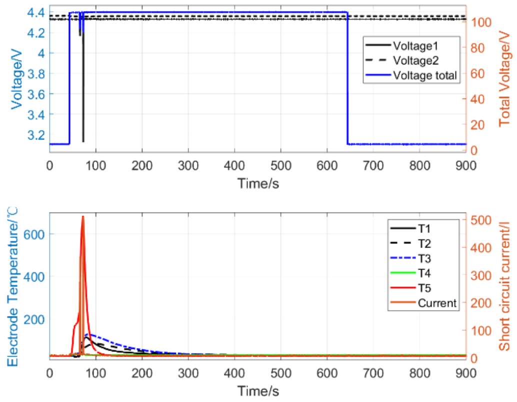
</td><td>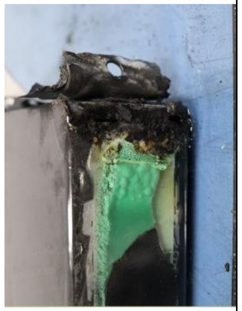</td><td>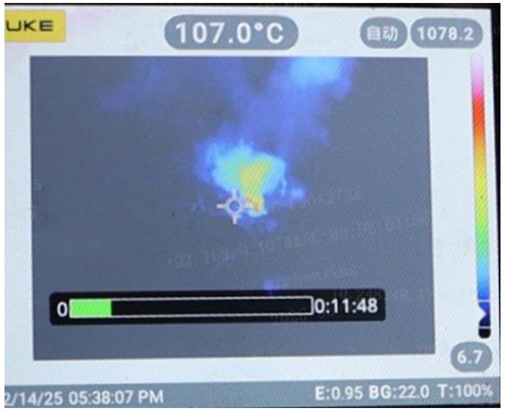
</td><td>Arc</td></tr>
<tr><td>17</td><td>7.2</td><td>103</td><td>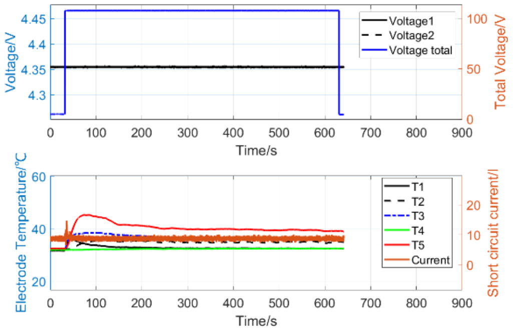
</td><td>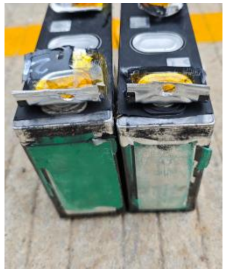</td><td>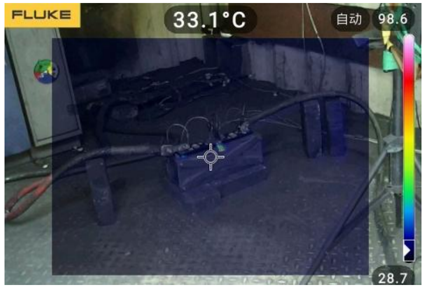
</td><td>Flashes</td></tr>
</table>

18	11.5	149.6	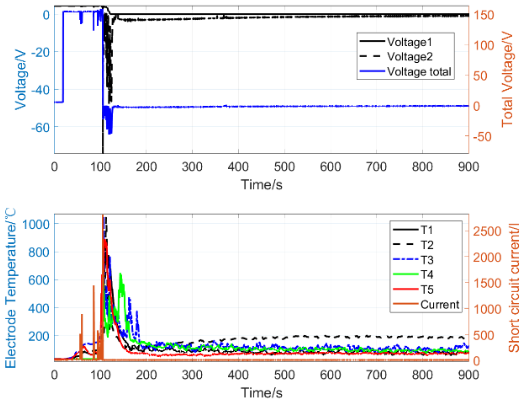	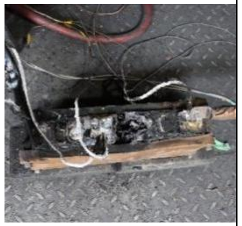	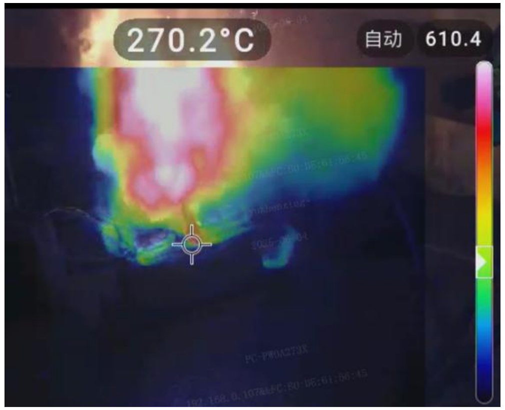	Arc &TR
19	11.5	158.9	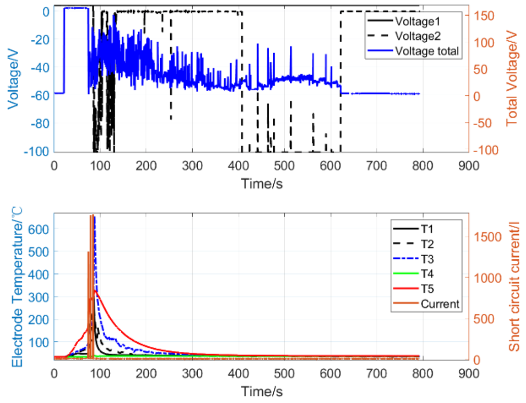	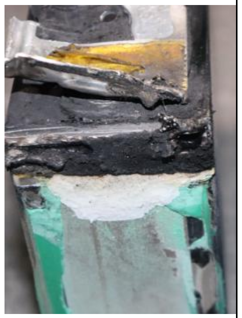	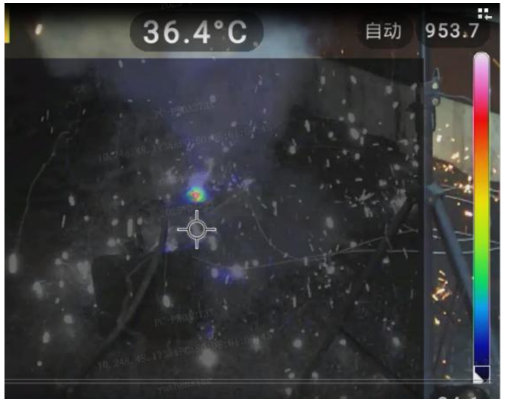	Arc

20	14.5	191.1	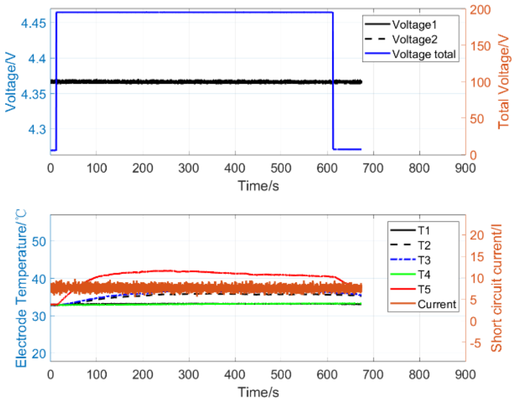	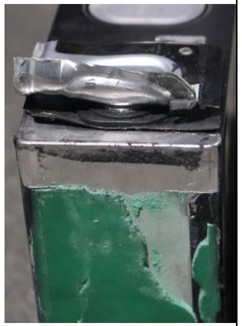	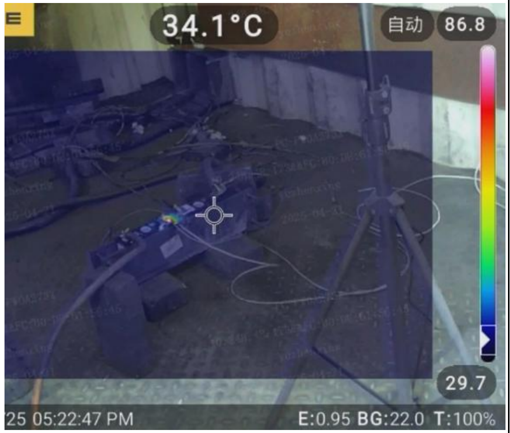	Flashes
21	14.5	206.8	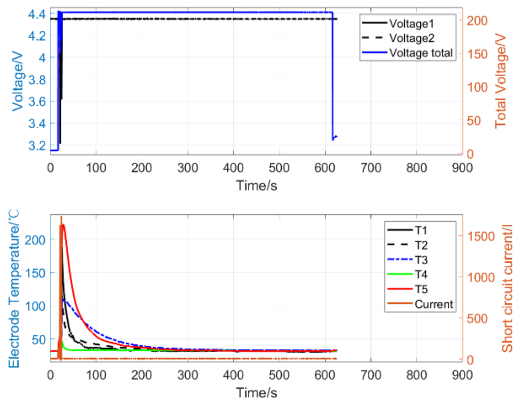	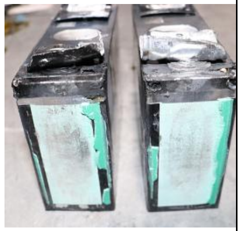	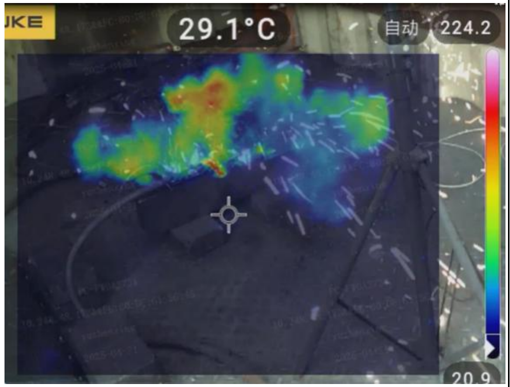	Arc

<table>
<tr><td></td><td></td><td></td><td></td><td></td><td></td><td></td></tr>
<tr><td>22</td><td>21</td><td>276.5</td><td>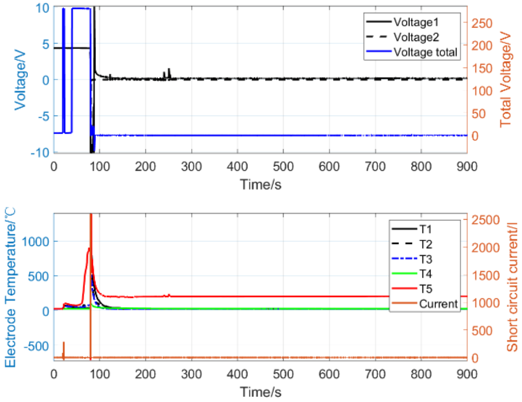
</td><td>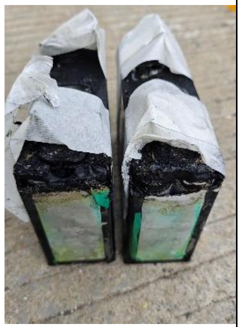</td><td>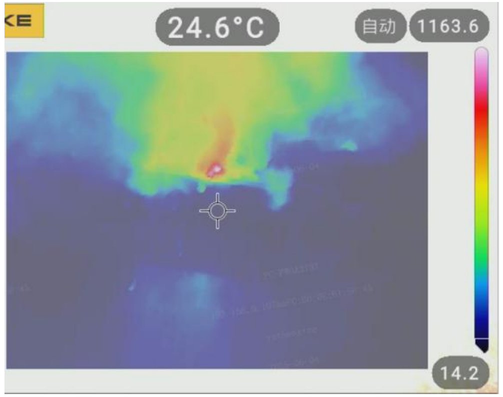
</td><td>Arc</td></tr>
<tr><td>23</td><td>21</td><td>297.3</td><td>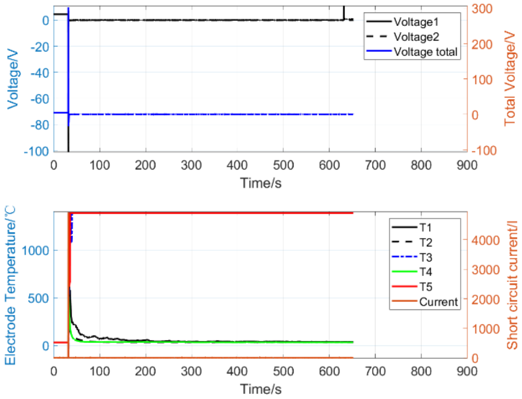
</td><td>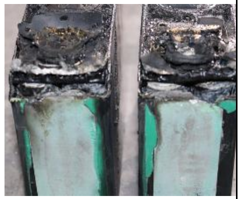</td><td>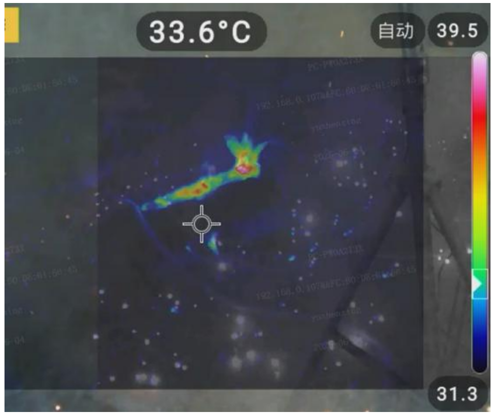
</td><td>Arc</td></tr>
</table>

Supplementary Table 2. The parameters of commercial prismatic Lithium-Nickel-Manganese-Cobalt-Oxide cells

Parameters	Unit	Specifications
Cell mass	g	1783
Size (w*l*h)	mm	52.2*148.7*103.2
Cathode active material	/	$LiNi_xCo_yMn_{1-x-y}O_2$ (x > 0.6)
Anode active material	/	Graphite
Rated capacity	Ah	≥117 @ 1/3 C
Rated voltage	V	3.75 @ 1/3 C
Minimum voltage	V	2.8
Maximum voltage	V	4.4
PPS Resistance	Ω	80-900

Supplementary Table 3. The composition and weight of the ejecta from cell thermal runaway

Parameters	Unit	Test1	Test2	Test3
Powder material mass	g	526.6	580.3	561.3
Metal fragments mass	g	311	373.4	346.5
Weight loss rate	%	46.5	53.0	50.4
Mass ratio Powder/Metal	/	1.69	1.55	1.62

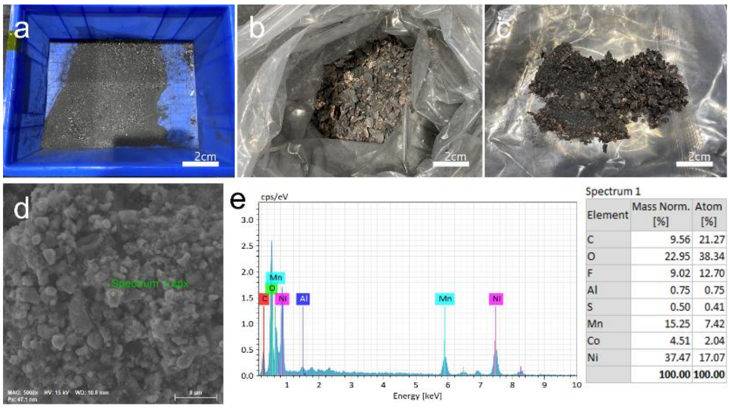

Element	Mass Norm. [%]	Atom [%]
C	9.56	21.27
O	22.95	38.34
F	9.02	12.70
Al	0.75	0.75
S	0.50	0.41
Mn	15.25	7.42
Co	4.51	2.04
Ni	37.47	17.07
	**100.00**	**100.00**

Supplementary Figure 4. The characterization results of the ejecta. a Image of the powder cathode material; b image of metal fragments; c image of mixture ejecta with a 1.62 mass ratio; d Scanning Electron Microscope image, and e Energy Dispersive Spectroscopy result of the powder material in the ejecta.
