# Supplementary material for: Thermal runaway-induced short-circuit arc in highly integrated lithium-ion battery systems: mechanisms, thresholds, and mitigation strategies
Source: Commun Eng. 2026 Apr 6;5:101. doi: 10.1038/s44172-026-00657-w (PMC13234359; doi:10.1038/s44172-026-00657-w)
Supplement: Supplementary file 2 — Description of Additional Supplementary Files [file 44172_2026_657_MOESM2_ESM.pdf]

Description of Additional Supplementary Files:

File: Supplementary Data 1

Description: The results of case-case mode short-circuit arc test under different voltages and electrode spacings.

File: Supplementary Movie 1

Description: The battery system experiment video of case-case short circuit mode with 7.2 mm electrode spacing and voltage of 53.1 V.

File: Supplementary Movie 2

Description: The battery system experiment video of case-case short circuit mode with 7.2 mm electrode spacing and voltage of 116.9 V.

File: Supplementary Movie 3

Description: The battery system experiment video of case-case short circuit mode with 7.2 mm electrode spacing and voltage of 159.4 V.

File: Supplementary Movie 4

Description: The battery system experiment video of case-busbar short circuit mode with 11 mm electrode spacing and voltage of 113.0 V.

File: Supplementary Movie 5

Description: The battery system experiment video of busbar-busbar short circuit mode with 11.5 mm electrode spacing and voltage of 120.5 V.

File: Supplementary Movie 6

Description: The cells experiment video of case-case short circuit mode with 7.2 mm electrode spacing and voltage of 55.8 V.

File: Supplementary Movie 7

Description: The cells experiment video of case-case short circuit mode with 7.2 mm electrode spacing and voltage of 91.2 V.

File: Supplementary Movie 8

Description: The cells experiment video of case-case short circuit mode with 7.2 mm electrode spacing and voltage of 103 V.

File: Supplementary Movie 9

Description: The cells experiment video of case-case short circuit mode with 7.2 mm electrode spacing and voltage of 117 V.

File: Supplementary Movie 10

Description: The cells experiment video of case-case short circuit mode with 1.2 mm electrode spacing and voltage of 71.7 V.

File: Supplementary Movie 11

Description: The cells experiment video of case-case short circuit mode with 11.5 mm electrode spacing and voltage of 149.6 V.
