# Supplementary figures and images for: Thermal runaway-induced short-circuit arc in highly integrated lithium-ion battery systems: mechanisms, thresholds, and mitigation strategies
Source: Commun Eng. 2026 Apr 6;5:101. doi: 10.1038/s44172-026-00657-w (PMC13234359; doi:10.1038/s44172-026-00657-w)

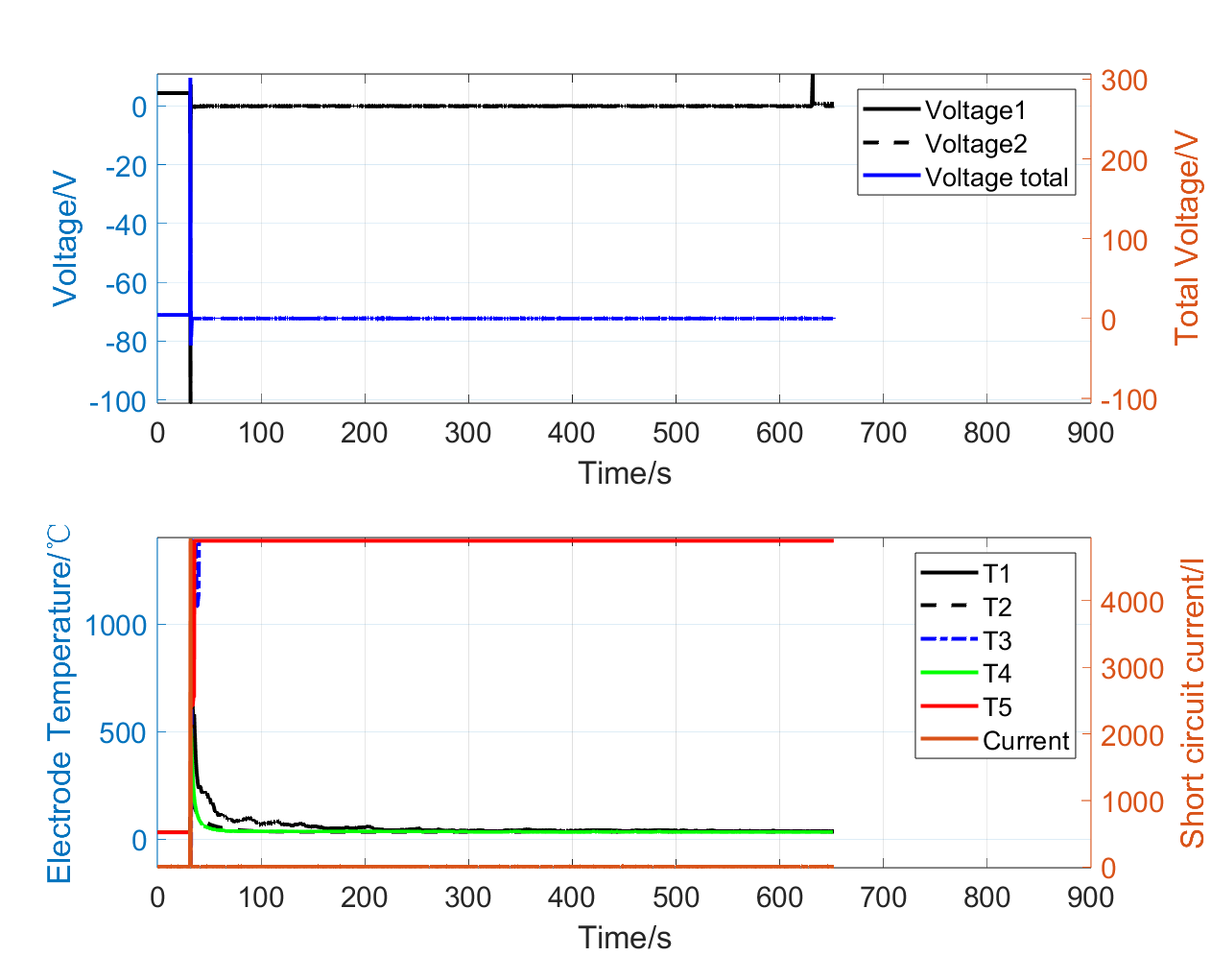

Supplement: Supplementary file 14 — Supplementary Data 1 [file 44172_2026_657_MOESM14_ESM.zip › 21mm-297.3V-Voltage-Temperature-Current Curves.tif]

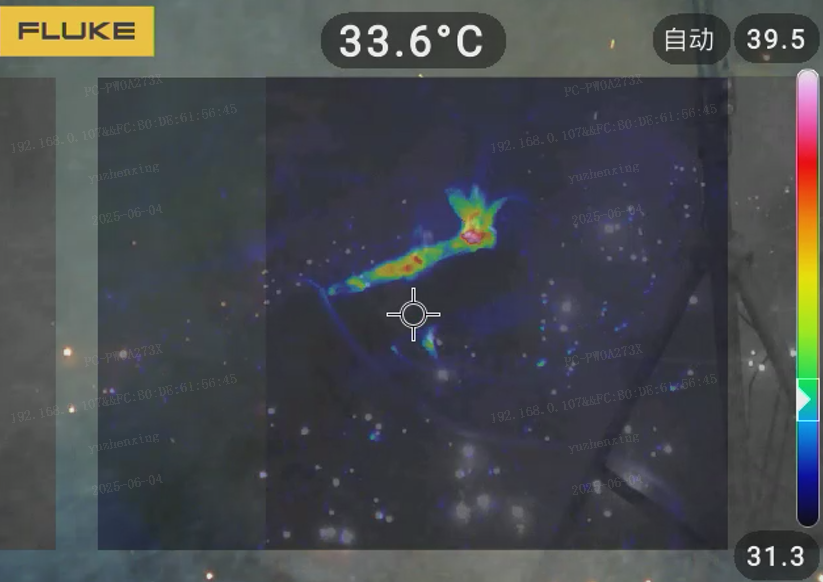

Supplement: Supplementary file 14 — Supplementary Data 1 [file 44172_2026_657_MOESM14_ESM.zip › 21mm-297.3V-Infrared Image.tif]

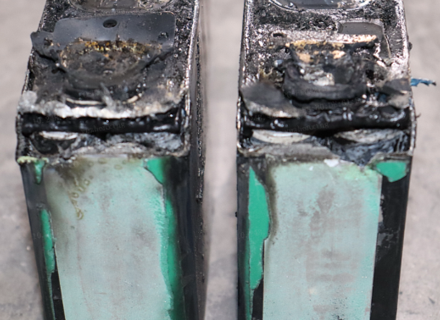

Supplement: Supplementary file 14 — Supplementary Data 1 [file 44172_2026_657_MOESM14_ESM.zip › 21mm-297.3V-Cells photo after test.tif]

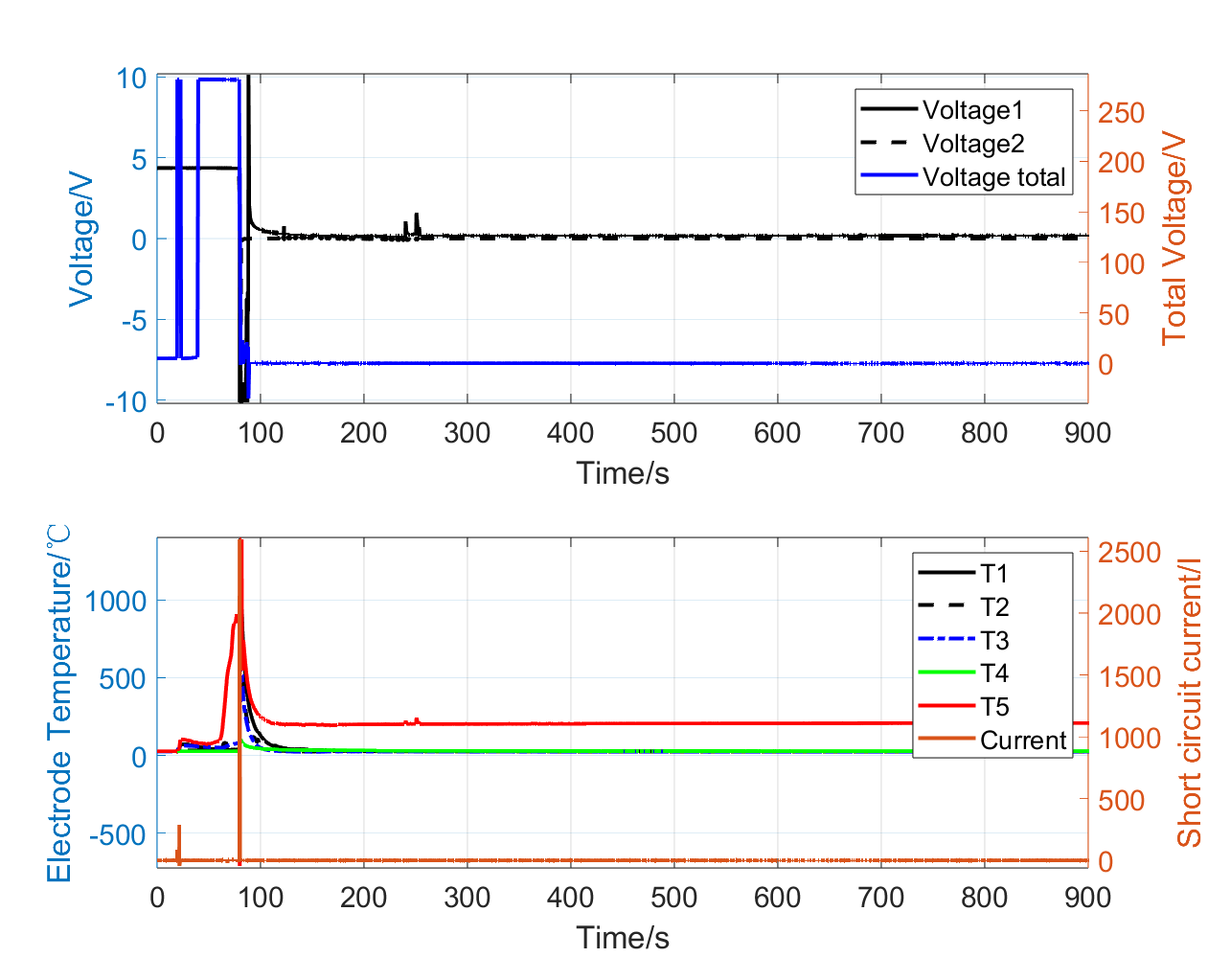

Supplement: Supplementary file 14 — Supplementary Data 1 [file 44172_2026_657_MOESM14_ESM.zip › 21mm-276.5V-Voltage-Temperature-Current Curves.tif]

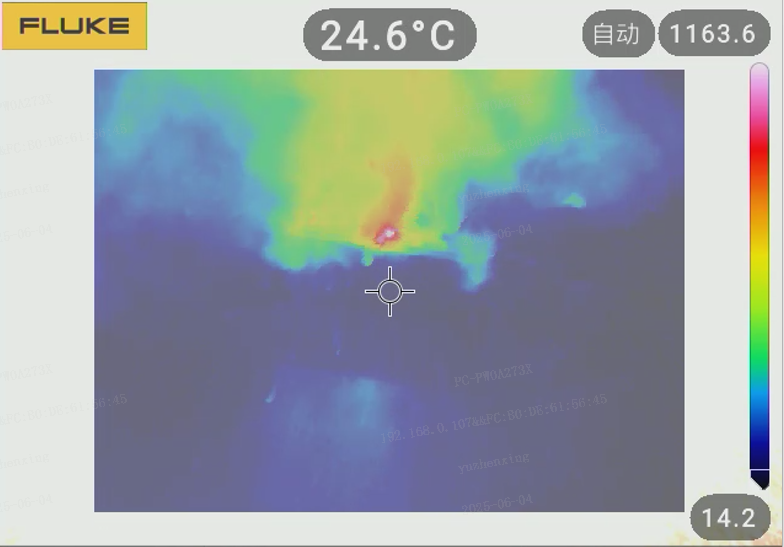

Supplement: Supplementary file 14 — Supplementary Data 1 [file 44172_2026_657_MOESM14_ESM.zip › 21mm-276.5V-Infrared Image.tif]

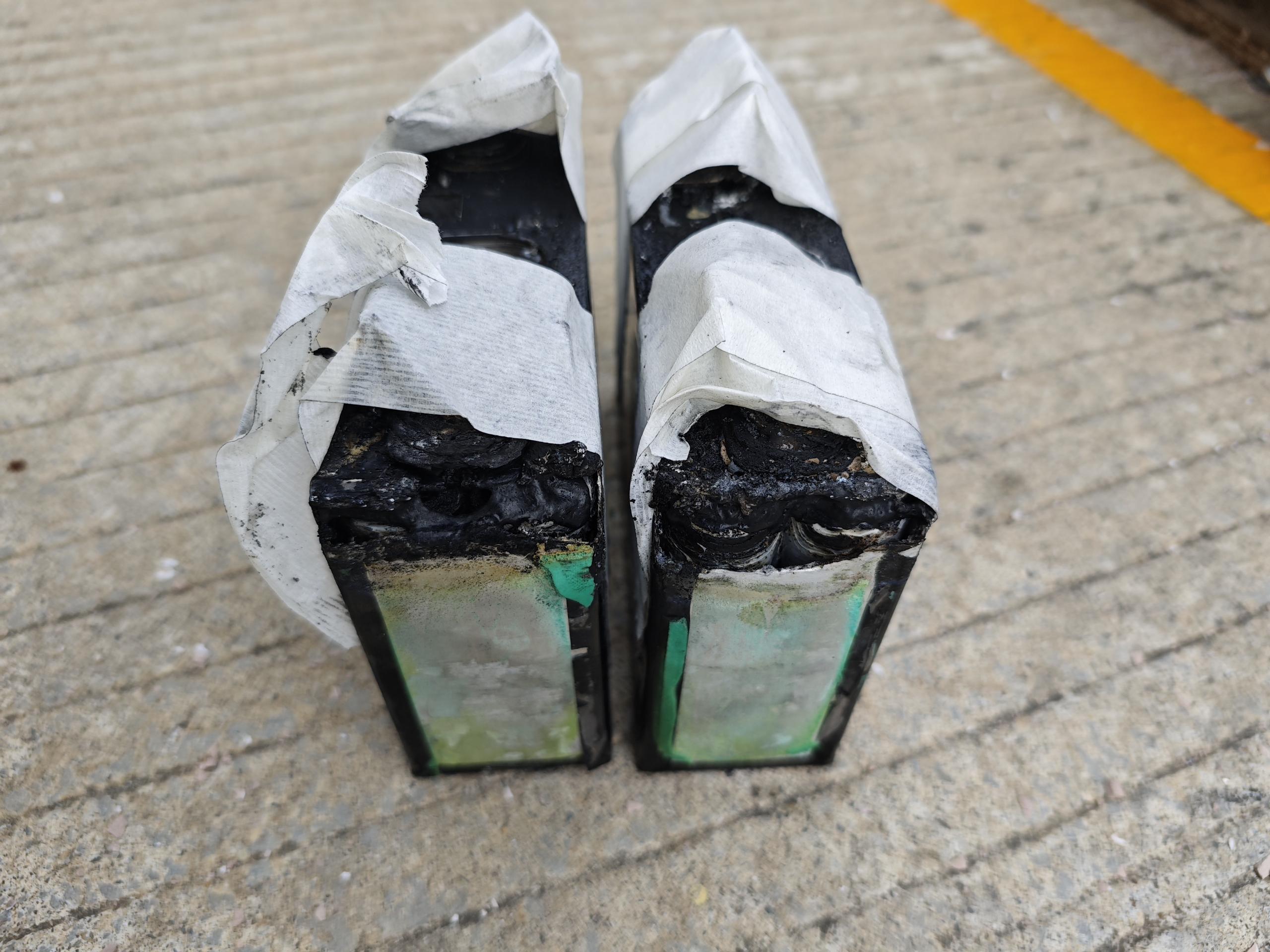

Supplement: Supplementary file 14 — Supplementary Data 1 [file 44172_2026_657_MOESM14_ESM.zip › 21mm-276.5V-Cells photo after test.tif]

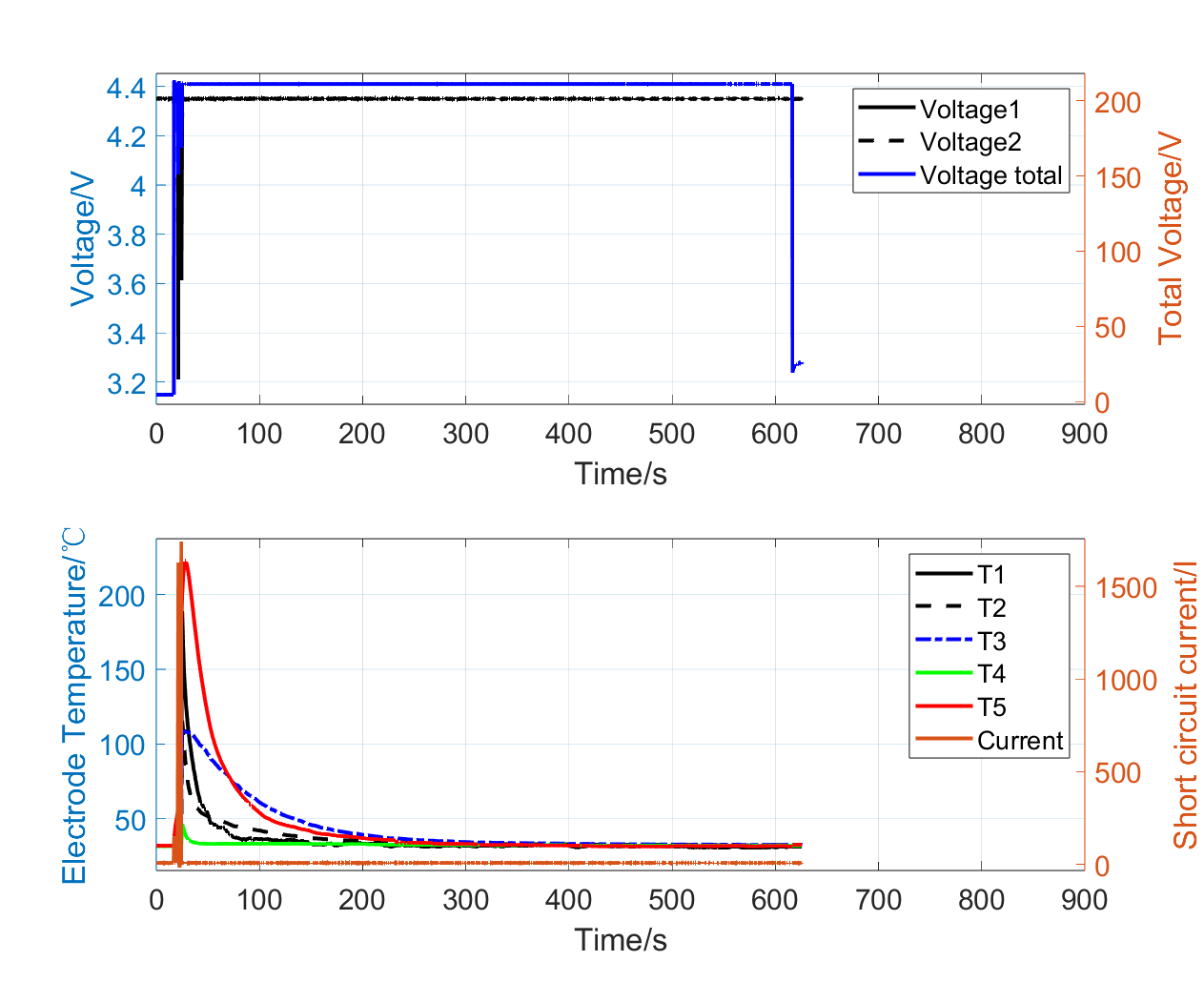

Supplement: Supplementary file 14 — Supplementary Data 1 [file 44172_2026_657_MOESM14_ESM.zip › 14.5mm-206.8V-Voltage-Temperature-Current Curves.tif]

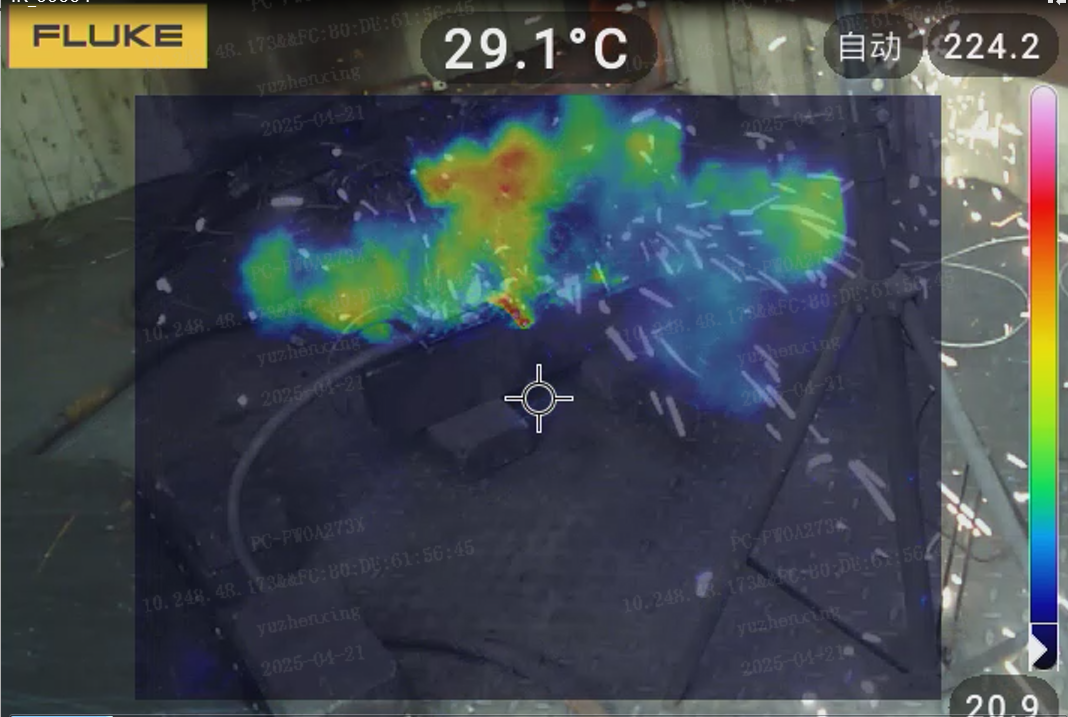

Supplement: Supplementary file 14 — Supplementary Data 1 [file 44172_2026_657_MOESM14_ESM.zip › 14.5mm-206.8V-Infrared Image.tif]

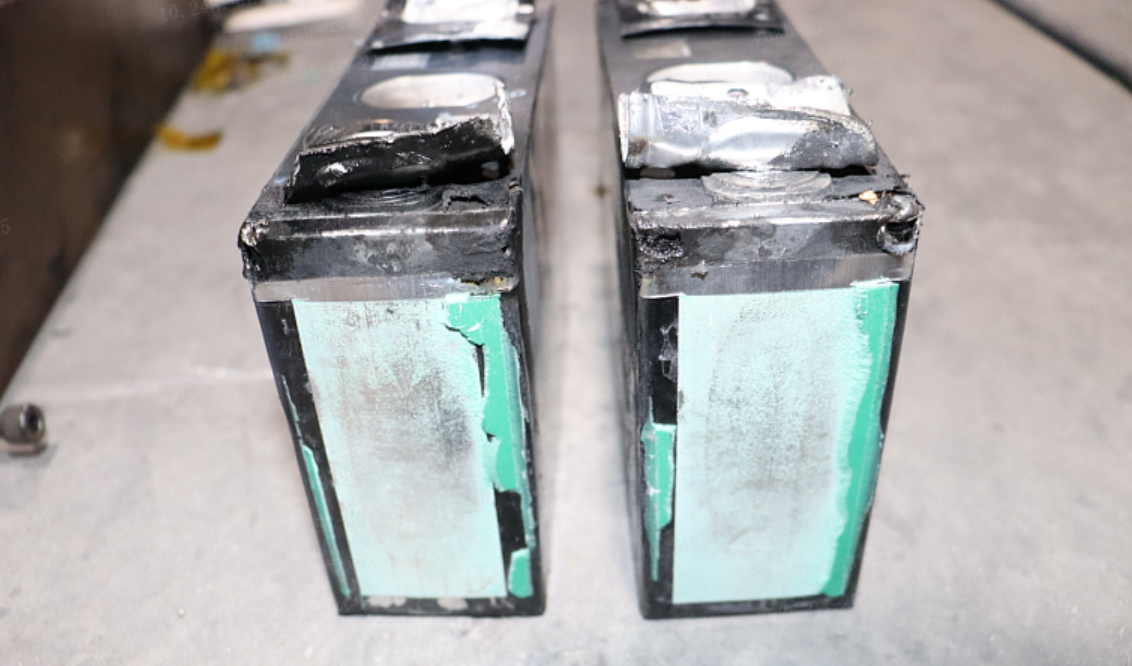

Supplement: Supplementary file 14 — Supplementary Data 1 [file 44172_2026_657_MOESM14_ESM.zip › 14.5mm-206.8V-Cells photo after test.tif]

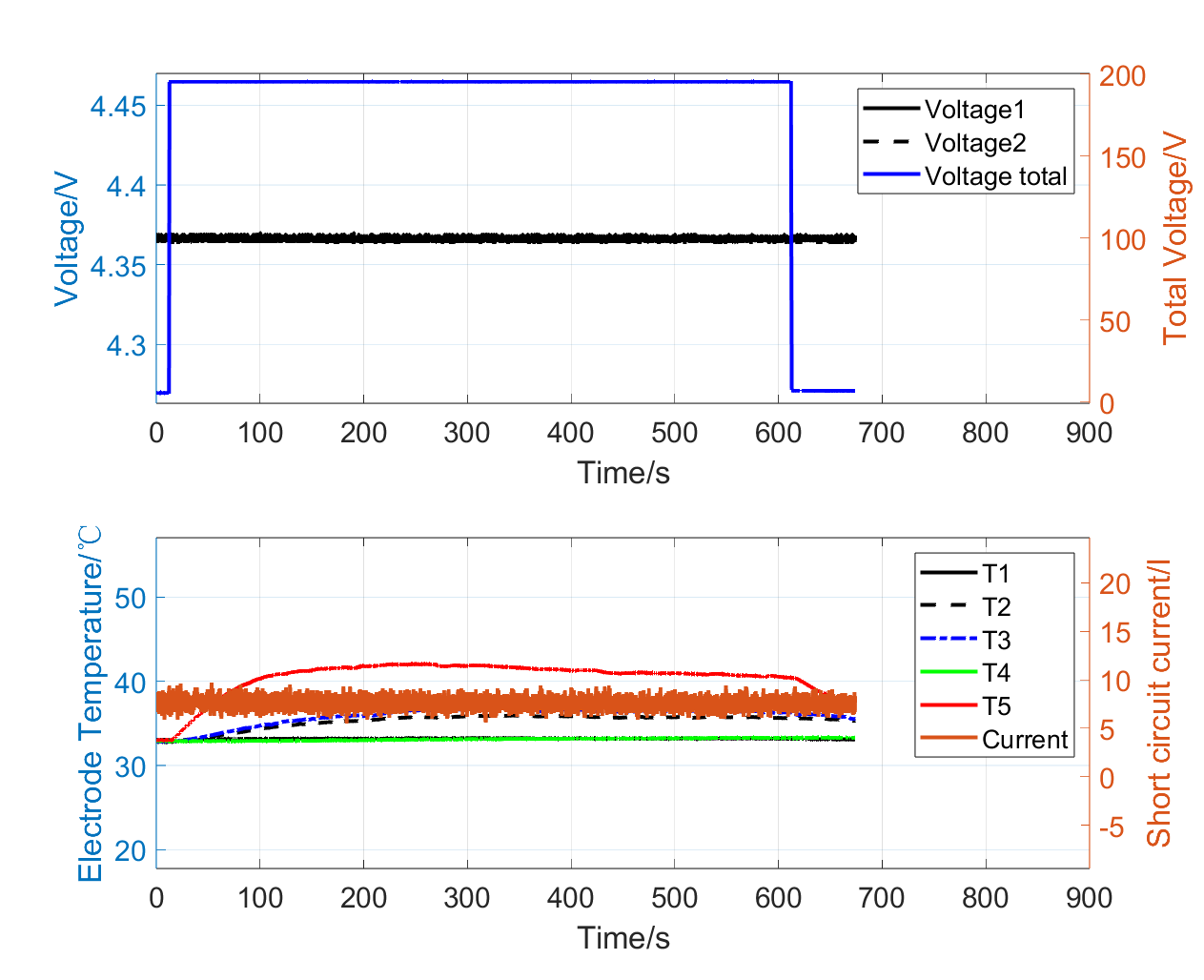

Supplement: Supplementary file 14 — Supplementary Data 1 [file 44172_2026_657_MOESM14_ESM.zip › 14.5mm-191.1V-Voltage-Temperature-Current Curves.tif]

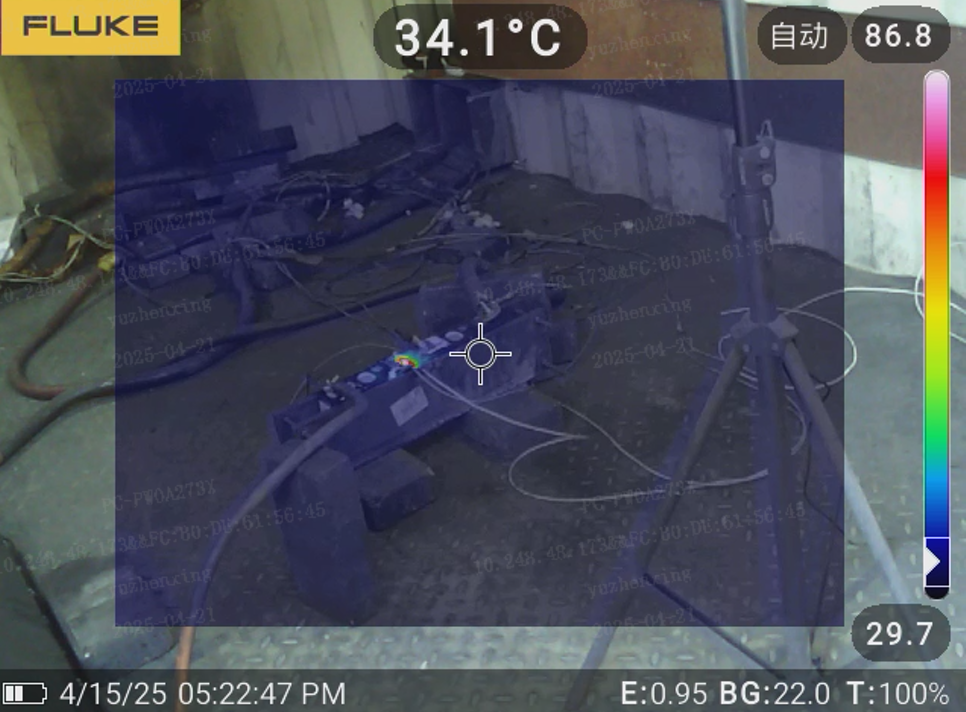

Supplement: Supplementary file 14 — Supplementary Data 1 [file 44172_2026_657_MOESM14_ESM.zip › 14.5mm-191.1V-Infrared Image.tif]

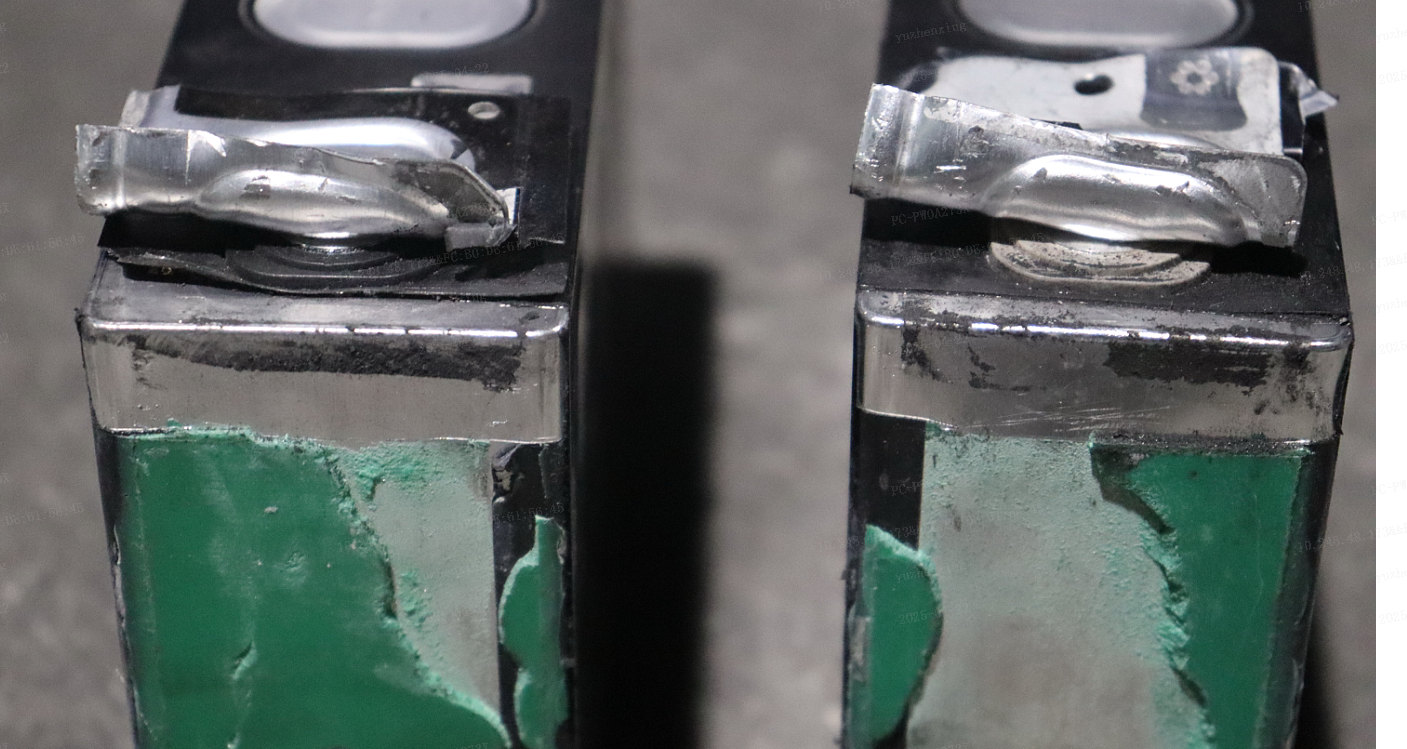

Supplement: Supplementary file 14 — Supplementary Data 1 [file 44172_2026_657_MOESM14_ESM.zip › 14.5mm-191.1V-Cells photo after test.tif]

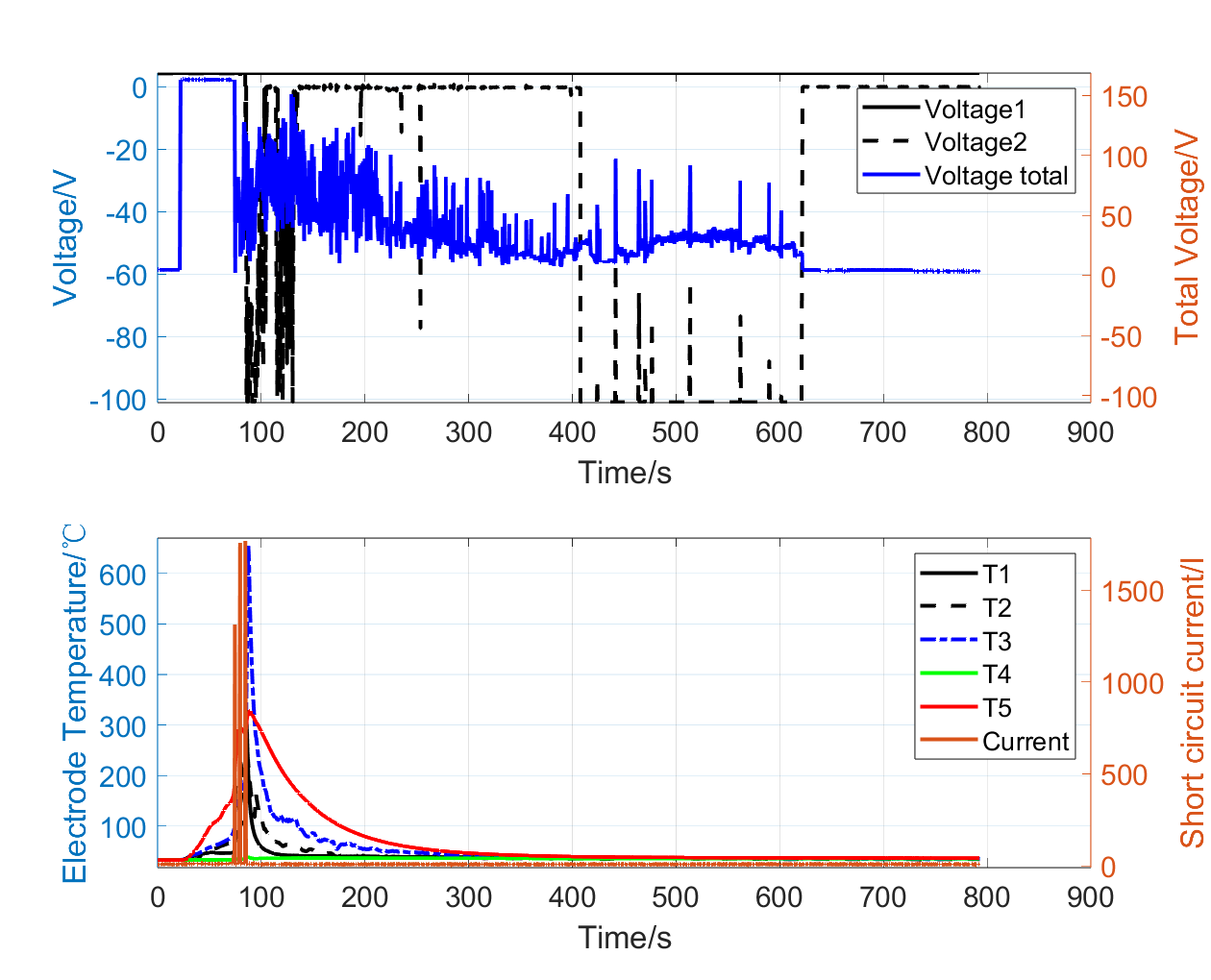

Supplement: Supplementary file 14 — Supplementary Data 1 [file 44172_2026_657_MOESM14_ESM.zip › 11.5mm-158.9V-Voltage-Temperature-Current Curves.tif]

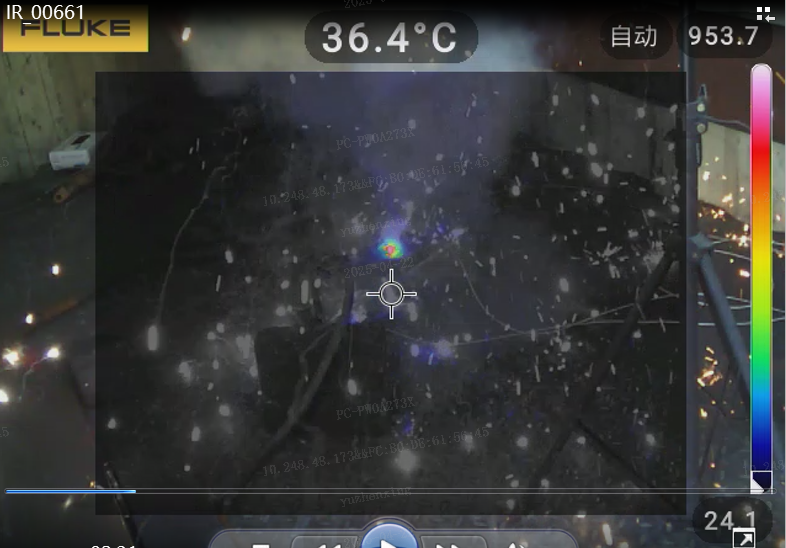

Supplement: Supplementary file 14 — Supplementary Data 1 [file 44172_2026_657_MOESM14_ESM.zip › 11.5mm-158.9V-Infrared Image.tif]

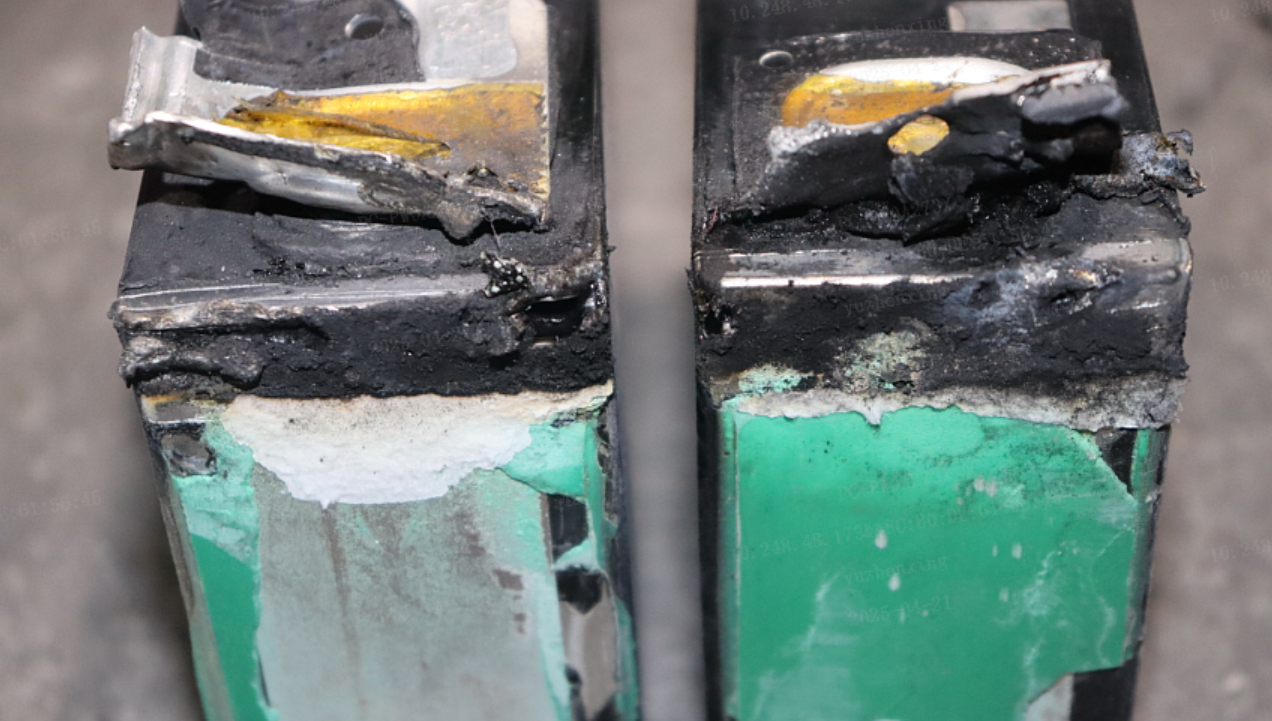

Supplement: Supplementary file 14 — Supplementary Data 1 [file 44172_2026_657_MOESM14_ESM.zip › 11.5mm-158.9V-Cells photo after test.tif]

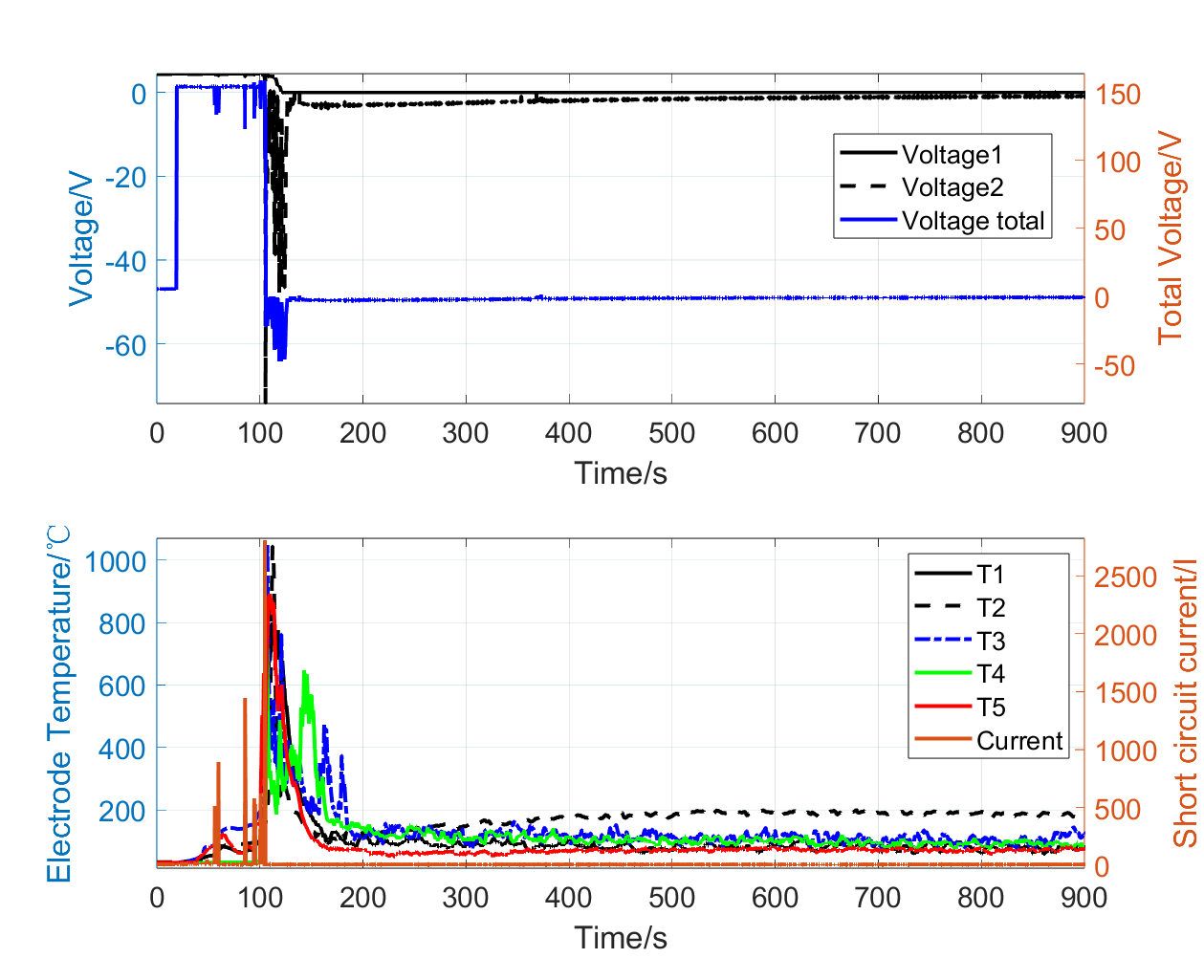

Supplement: Supplementary file 14 — Supplementary Data 1 [file 44172_2026_657_MOESM14_ESM.zip › 11.5mm-149.6V-Voltage-Temperature-Current Curves.tif]

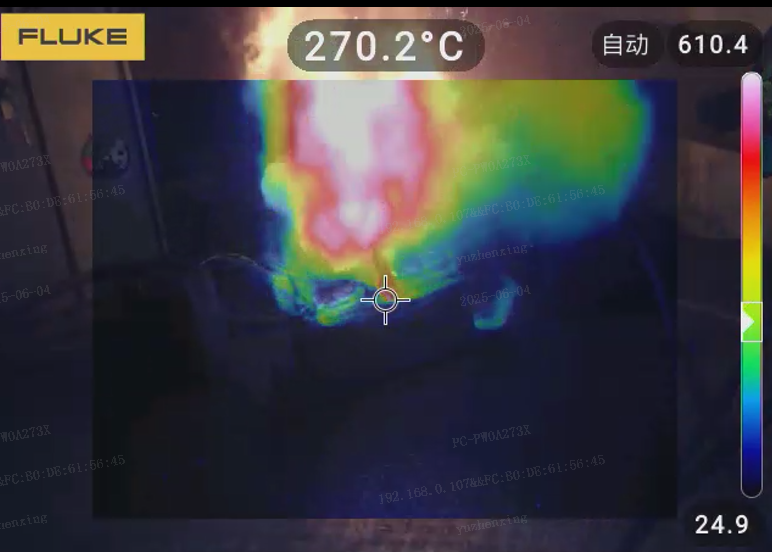

Supplement: Supplementary file 14 — Supplementary Data 1 [file 44172_2026_657_MOESM14_ESM.zip › 11.5mm-149.6V-Infrared Image.tif]

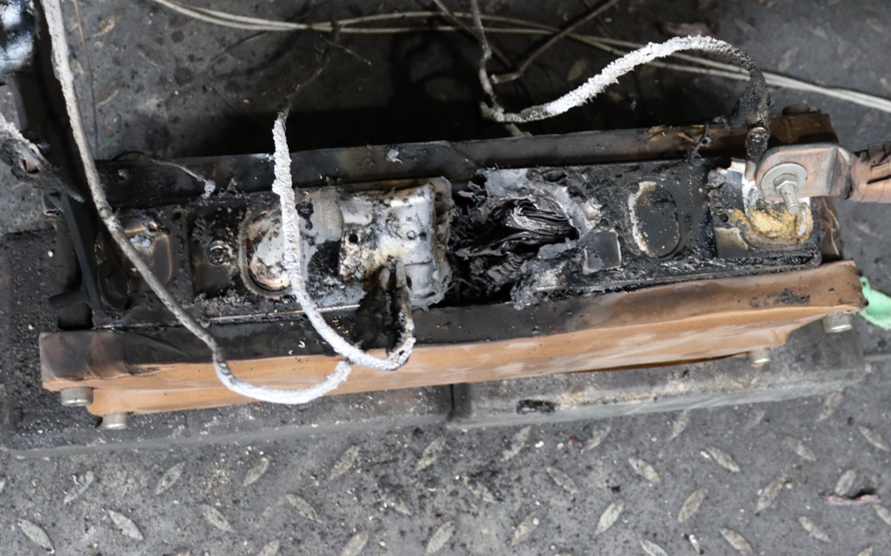

Supplement: Supplementary file 14 — Supplementary Data 1 [file 44172_2026_657_MOESM14_ESM.zip › 11.5mm-149.6V-Cells photo after test.tif]

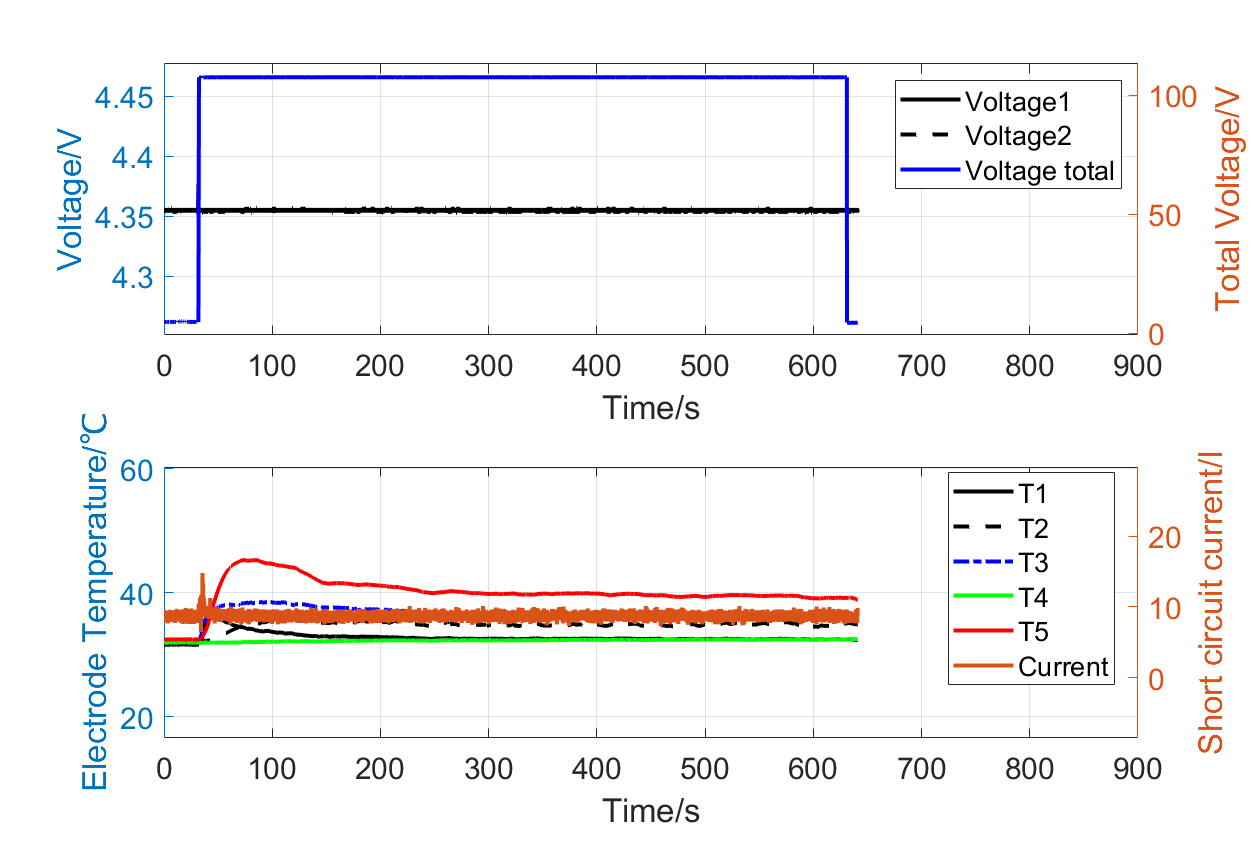

Supplement: Supplementary file 14 — Supplementary Data 1 [file 44172_2026_657_MOESM14_ESM.zip › 7.2mm-103V-Voltage-Temperature-Current Curves.tif]

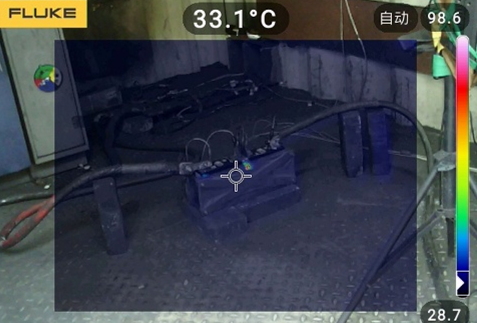

Supplement: Supplementary file 14 — Supplementary Data 1 [file 44172_2026_657_MOESM14_ESM.zip › 7.2mm-103V-Infrared Image.tif]

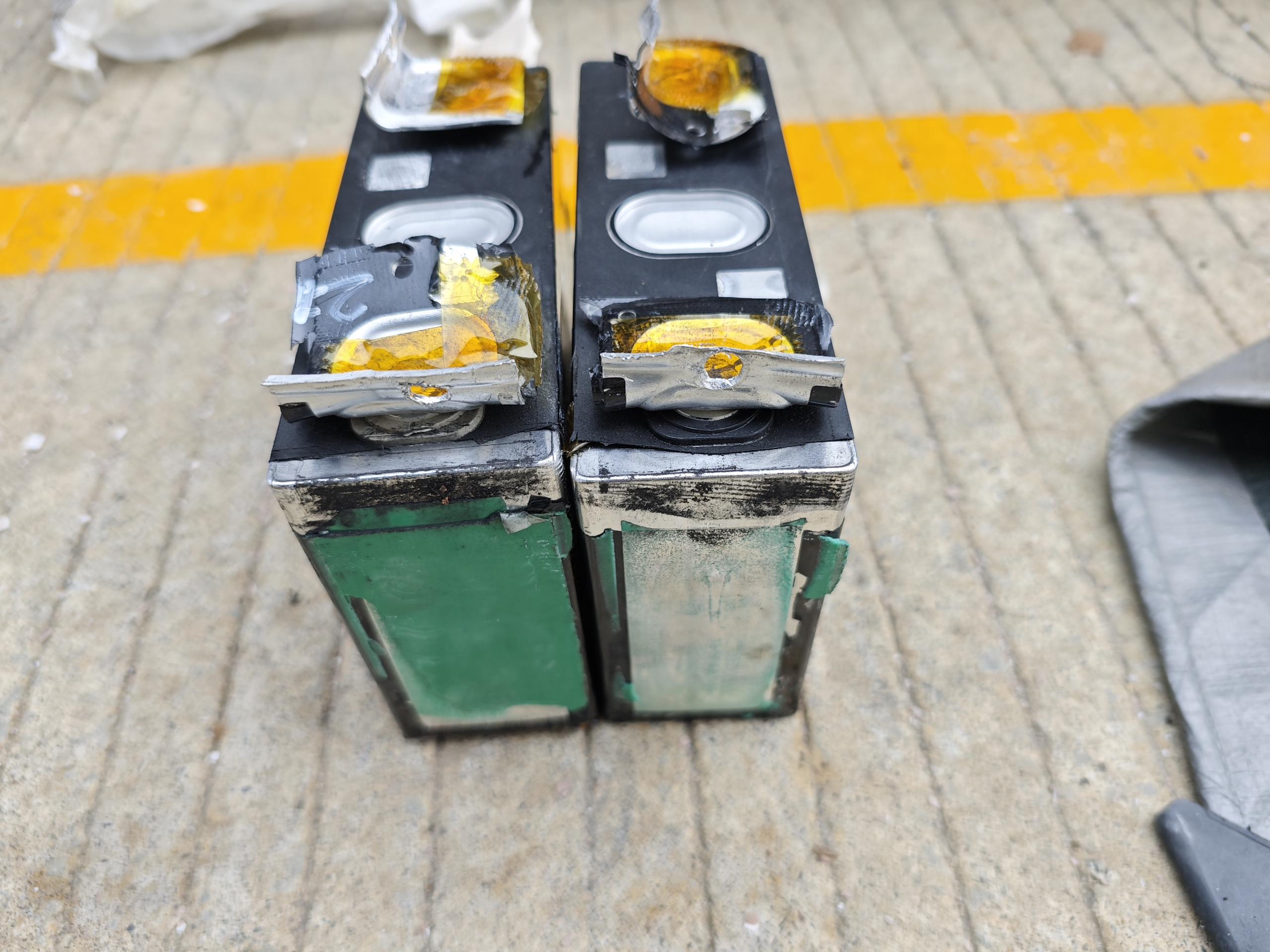

Supplement: Supplementary file 14 — Supplementary Data 1 [file 44172_2026_657_MOESM14_ESM.zip › 7.2mm-103V-Cells photo after test.tif]

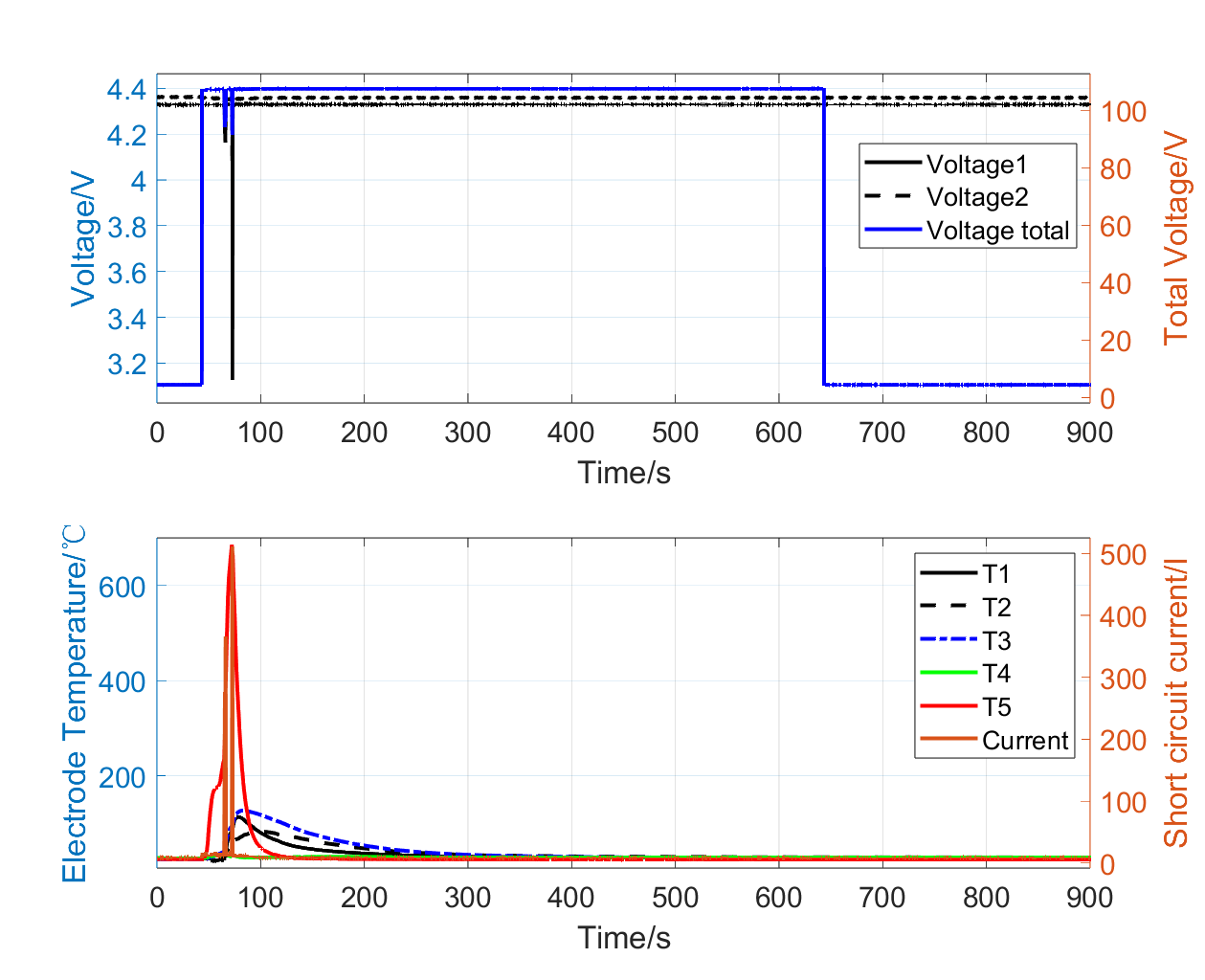

Supplement: Supplementary file 14 — Supplementary Data 1 [file 44172_2026_657_MOESM14_ESM.zip › 6.0mm-103V-Voltage-Temperature-Current Curves.tif]

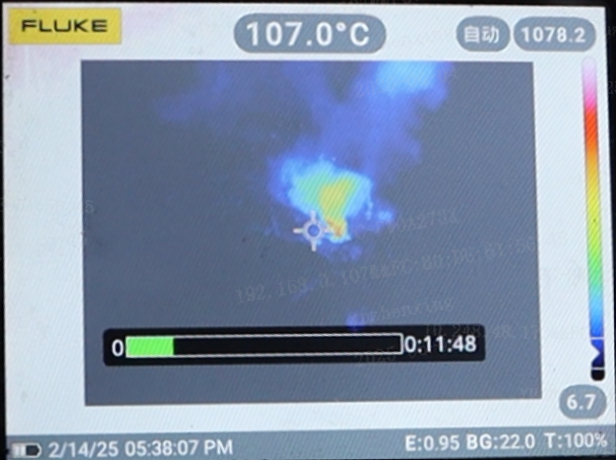

Supplement: Supplementary file 14 — Supplementary Data 1 [file 44172_2026_657_MOESM14_ESM.zip › 6.0mm-103V-Infrared Image.tif]

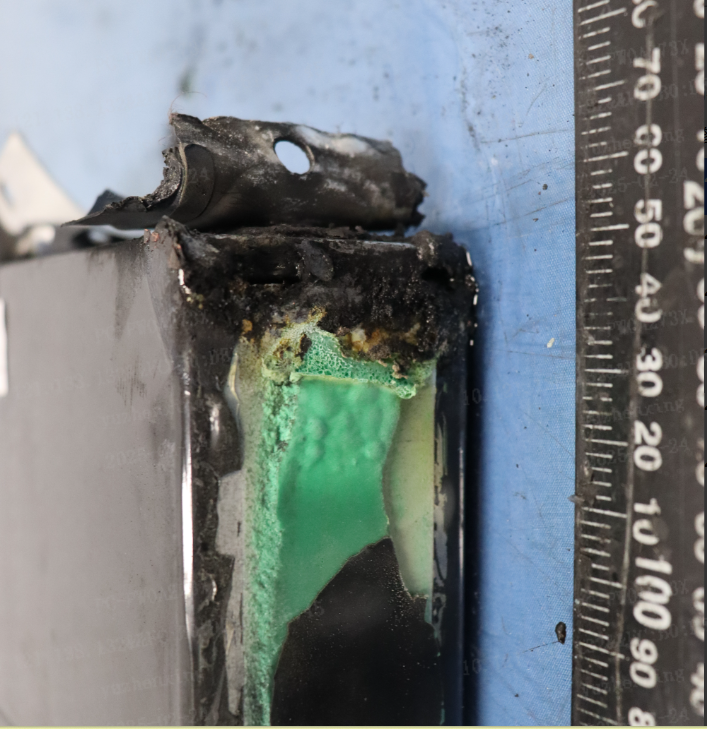

Supplement: Supplementary file 14 — Supplementary Data 1 [file 44172_2026_657_MOESM14_ESM.zip › 6.0mm-103V-Cells photo after test.tif]

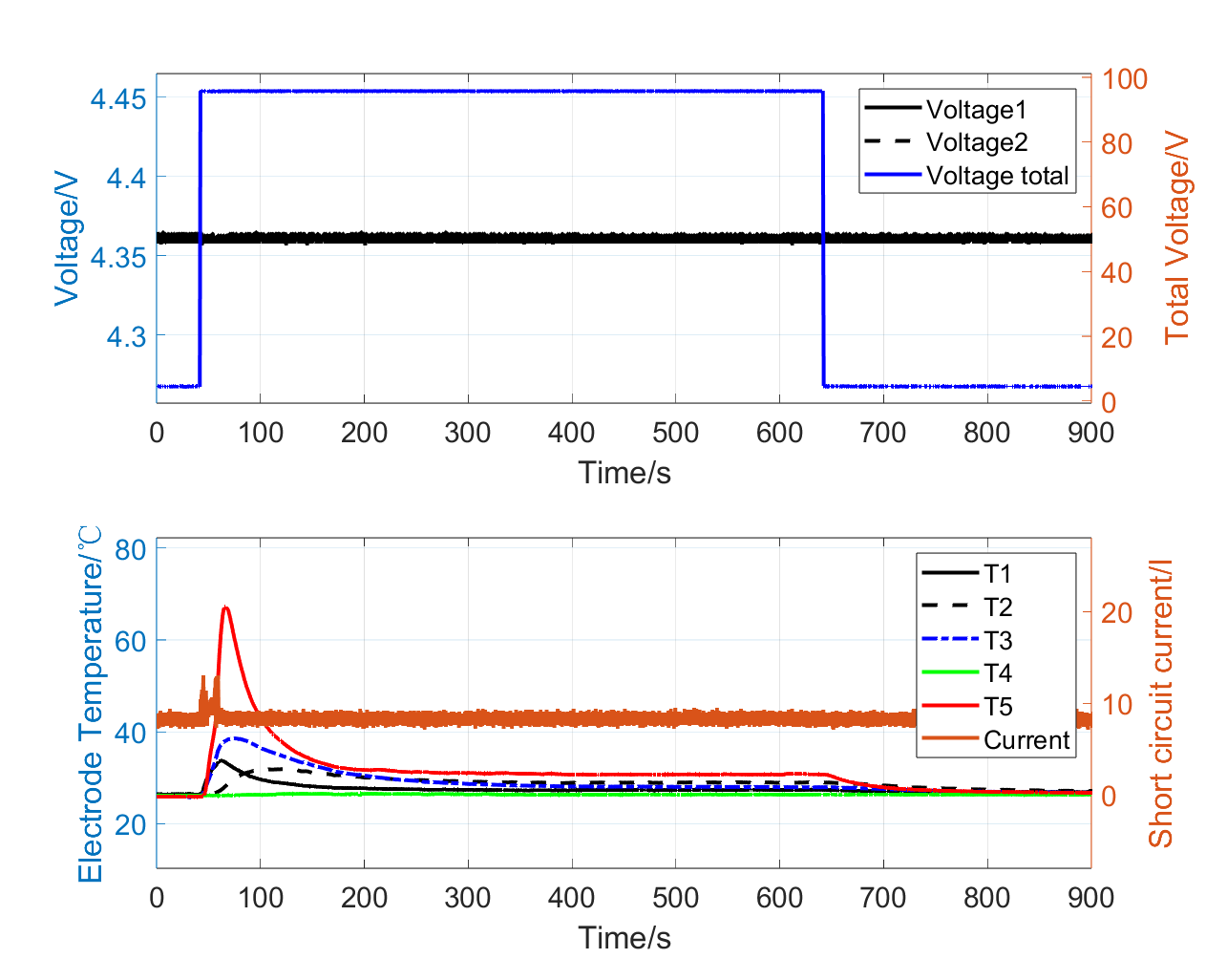

Supplement: Supplementary file 14 — Supplementary Data 1 [file 44172_2026_657_MOESM14_ESM.zip › 6.0mm-91.2V-Voltage-Temperature-Current Curves.tif]

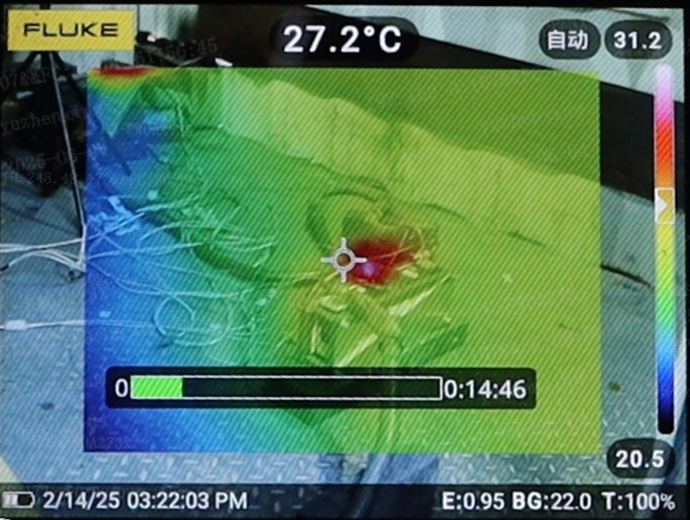

Supplement: Supplementary file 14 — Supplementary Data 1 [file 44172_2026_657_MOESM14_ESM.zip › 6.0mm-91.2V-Infrared Image.tif]

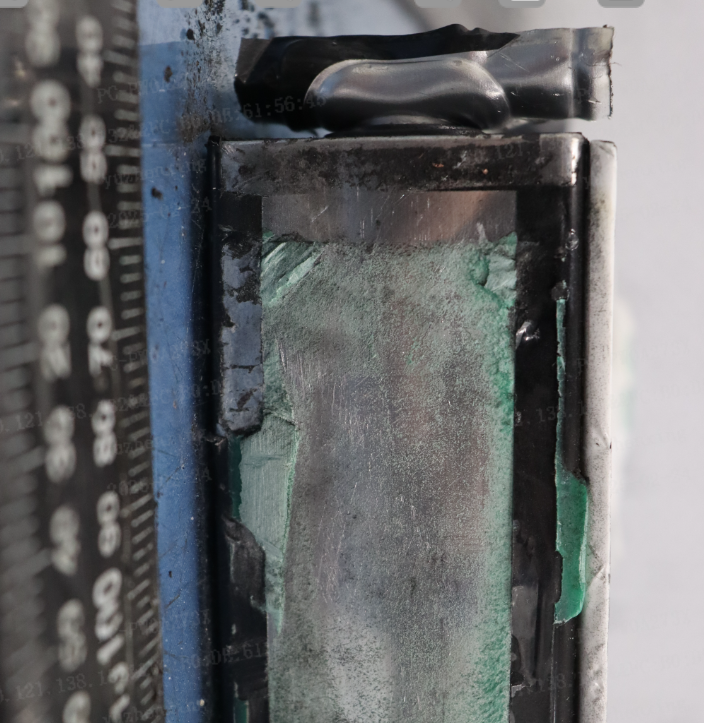

Supplement: Supplementary file 14 — Supplementary Data 1 [file 44172_2026_657_MOESM14_ESM.zip › 6.0mm-91.2V-Cells photo after test.tif]

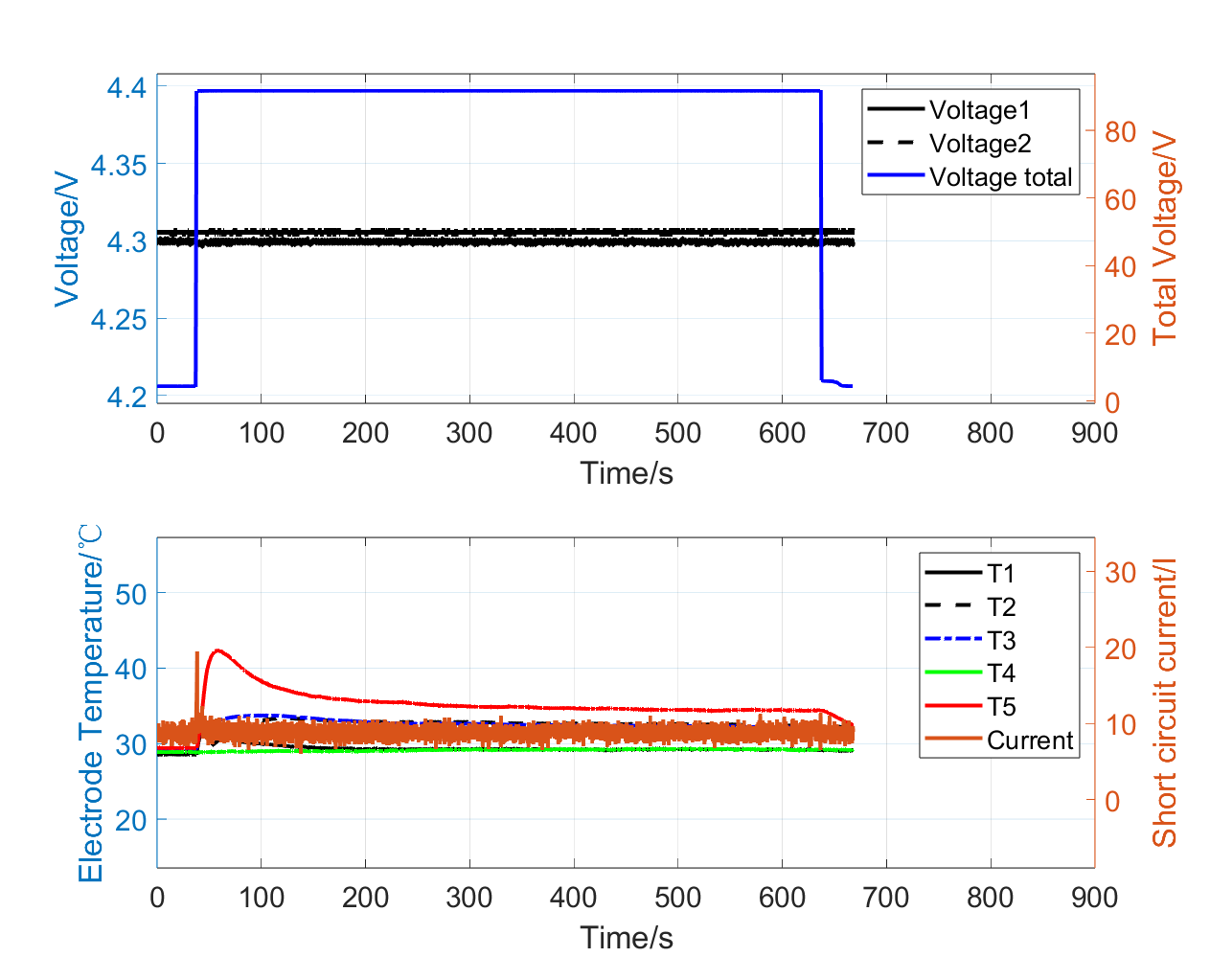

Supplement: Supplementary file 14 — Supplementary Data 1 [file 44172_2026_657_MOESM14_ESM.zip › 5.1mm-87.4V-Voltage-Temperature-Current Curves.tif]

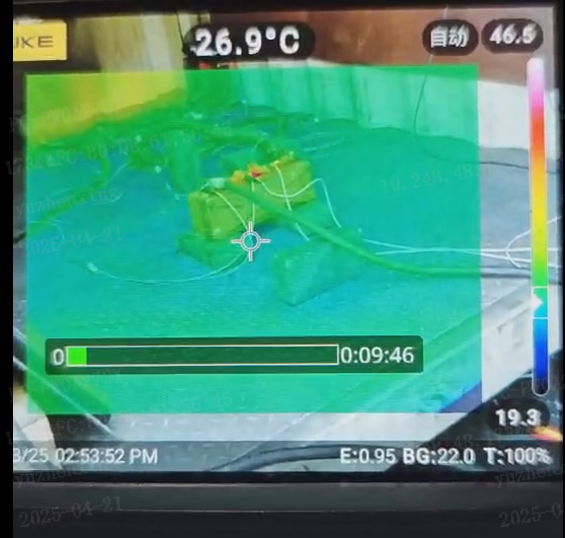

Supplement: Supplementary file 14 — Supplementary Data 1 [file 44172_2026_657_MOESM14_ESM.zip › 5.1mm-87.4V-Infrared Image.tif]

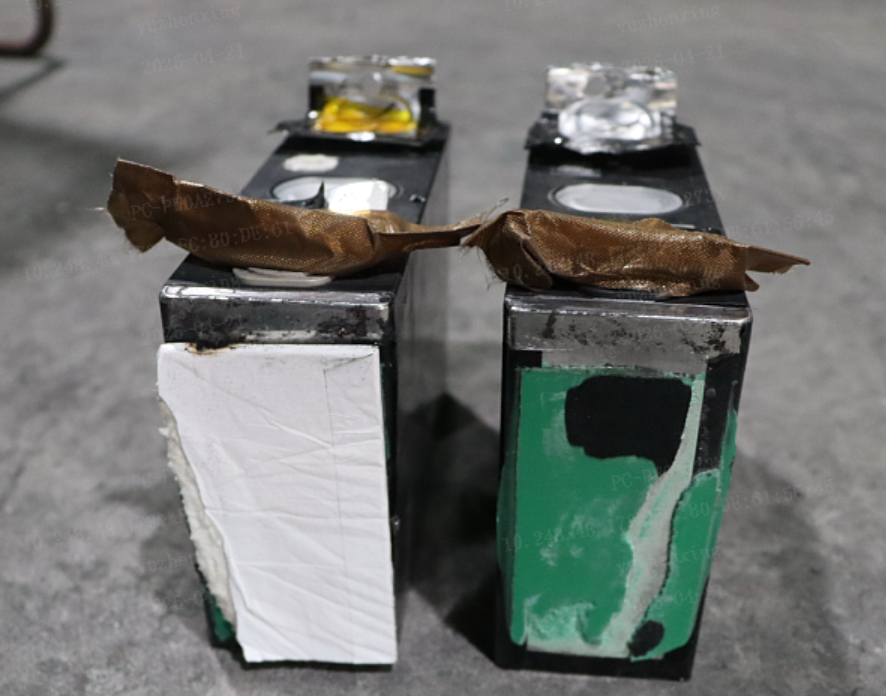

Supplement: Supplementary file 14 — Supplementary Data 1 [file 44172_2026_657_MOESM14_ESM.zip › 5.1mm-87.4V-Cells photo after test.tif]

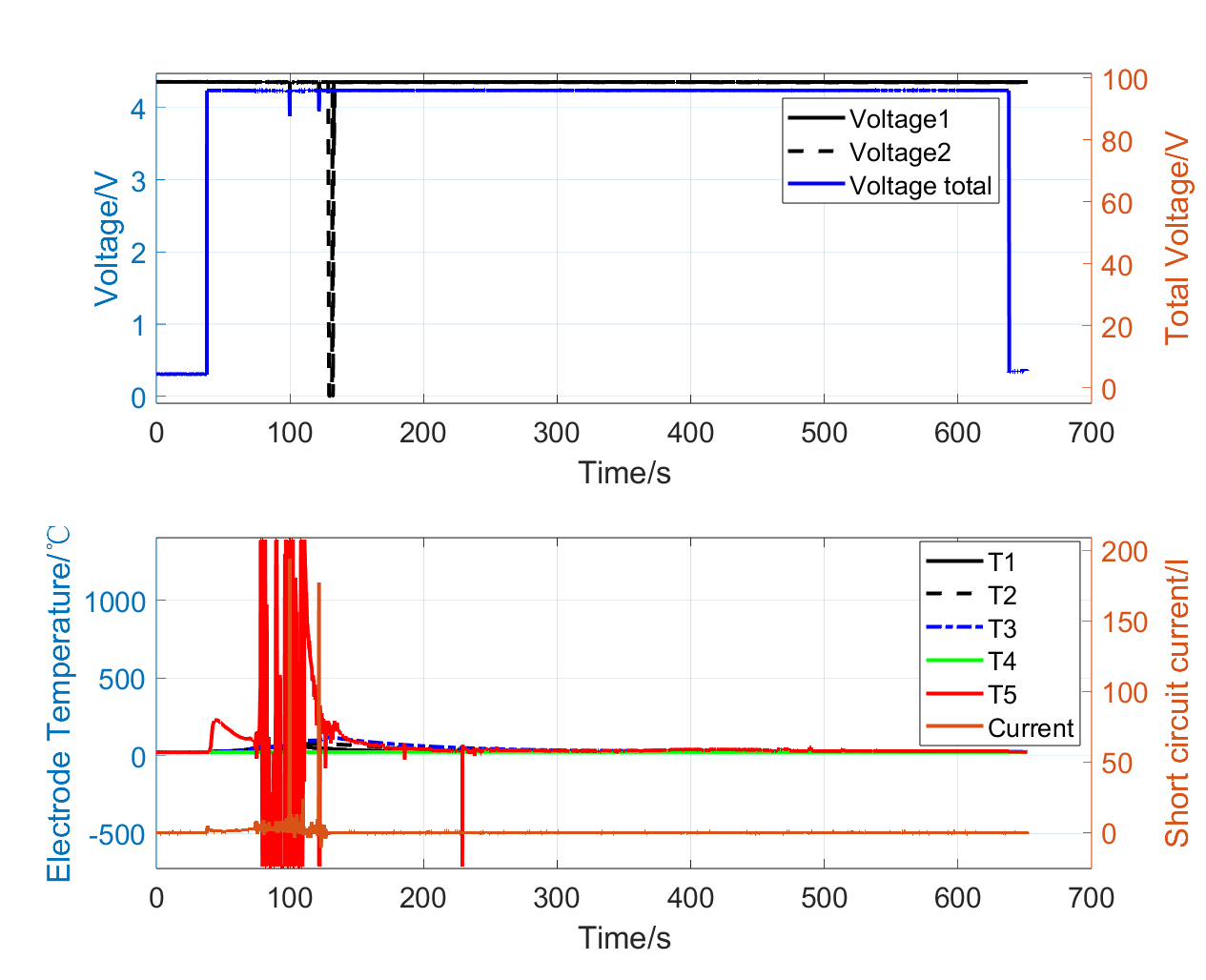

Supplement: Supplementary file 14 — Supplementary Data 1 [file 44172_2026_657_MOESM14_ESM.zip › 4.6mm-91.2V-Voltage-Temperature-Current Curves.tif]

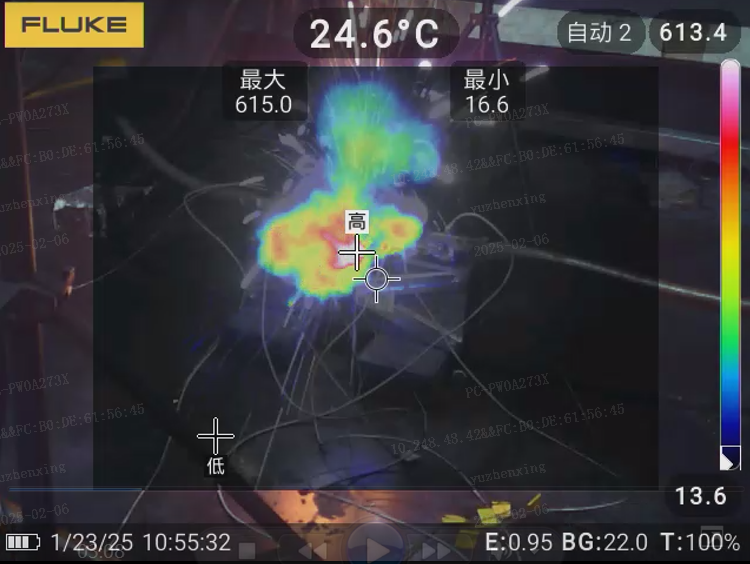

Supplement: Supplementary file 14 — Supplementary Data 1 [file 44172_2026_657_MOESM14_ESM.zip › 4.6mm-91.2V-Infrared Image.tif]

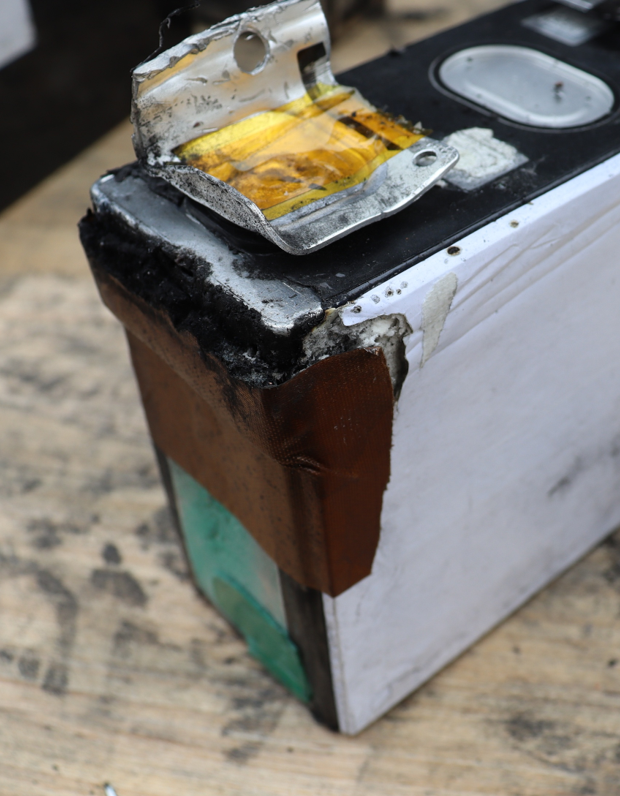

Supplement: Supplementary file 14 — Supplementary Data 1 [file 44172_2026_657_MOESM14_ESM.zip › 4.6mm-91.2V-Cells photo after test.tif]

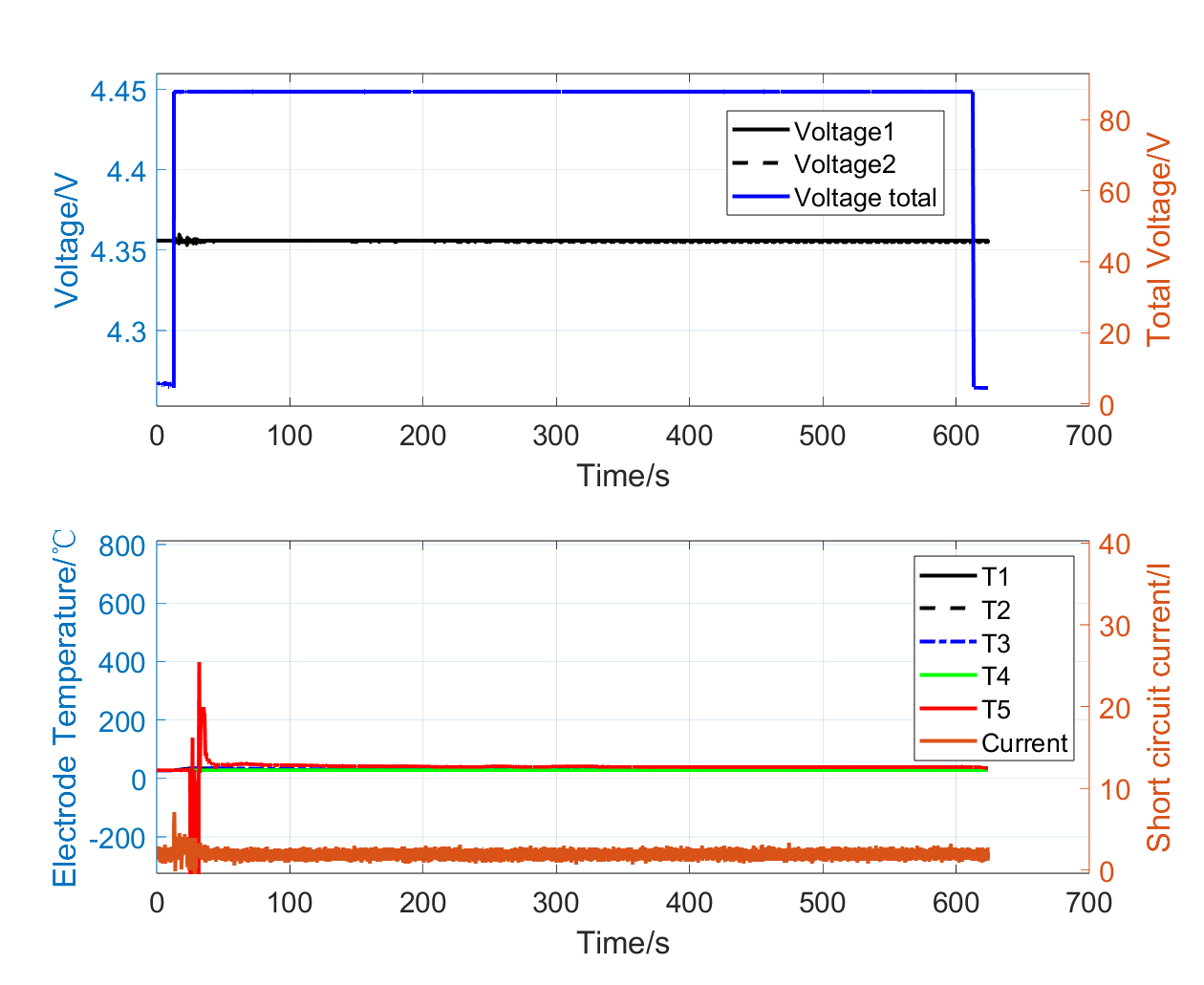

Supplement: Supplementary file 14 — Supplementary Data 1 [file 44172_2026_657_MOESM14_ESM.zip › 4.6mm-83.6V-Voltage-Temperature-Current Curves.tif]

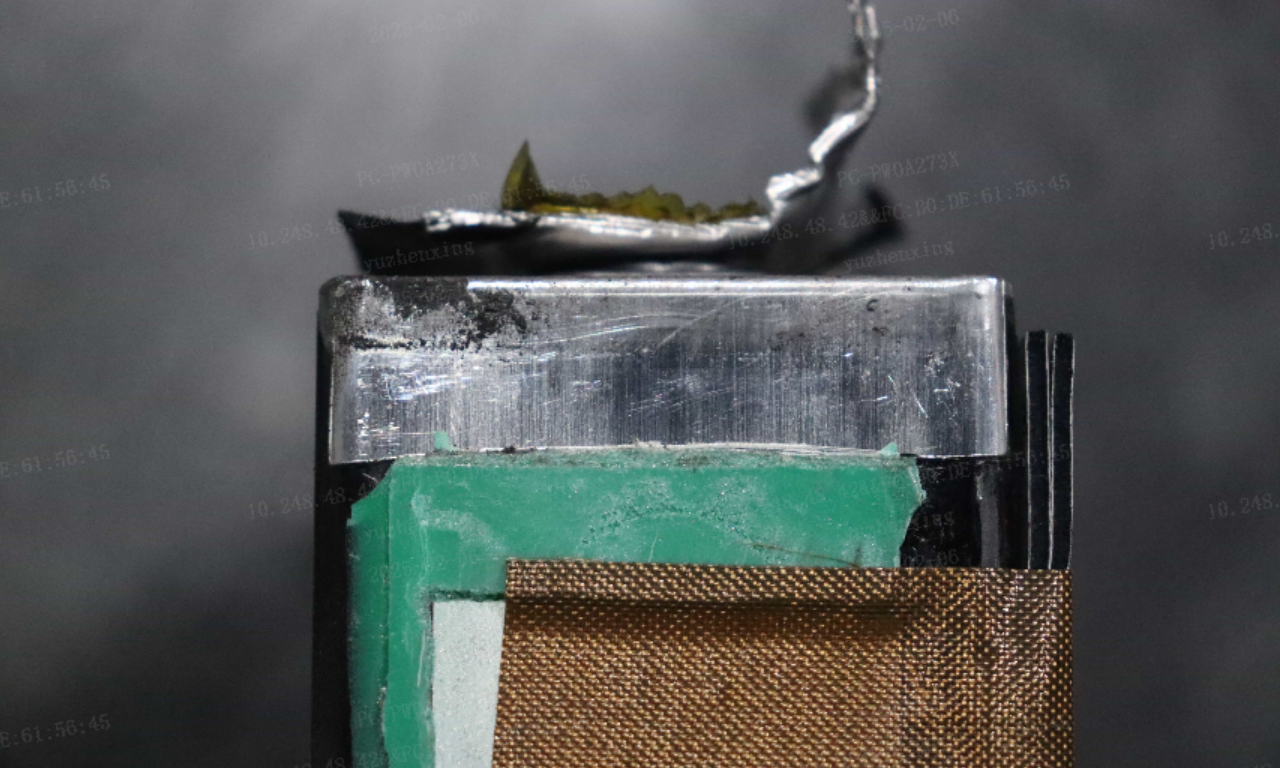

Supplement: Supplementary file 14 — Supplementary Data 1 [file 44172_2026_657_MOESM14_ESM.zip › 4.6mm-83.6V-Cells photo after test.tif]

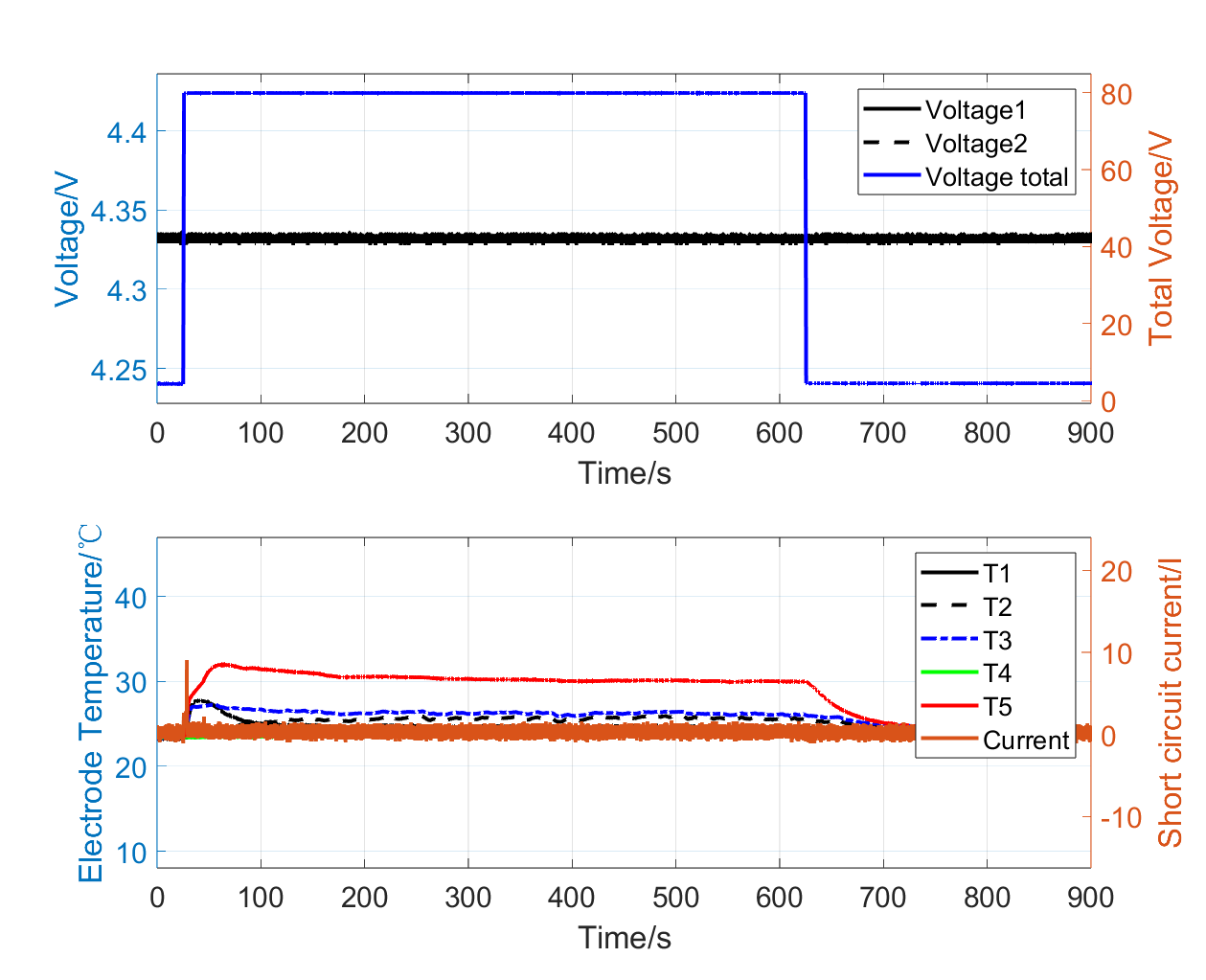

Supplement: Supplementary file 14 — Supplementary Data 1 [file 44172_2026_657_MOESM14_ESM.zip › 4.6mm-76.4V-Voltage-Temperature-Current Curves.tif]

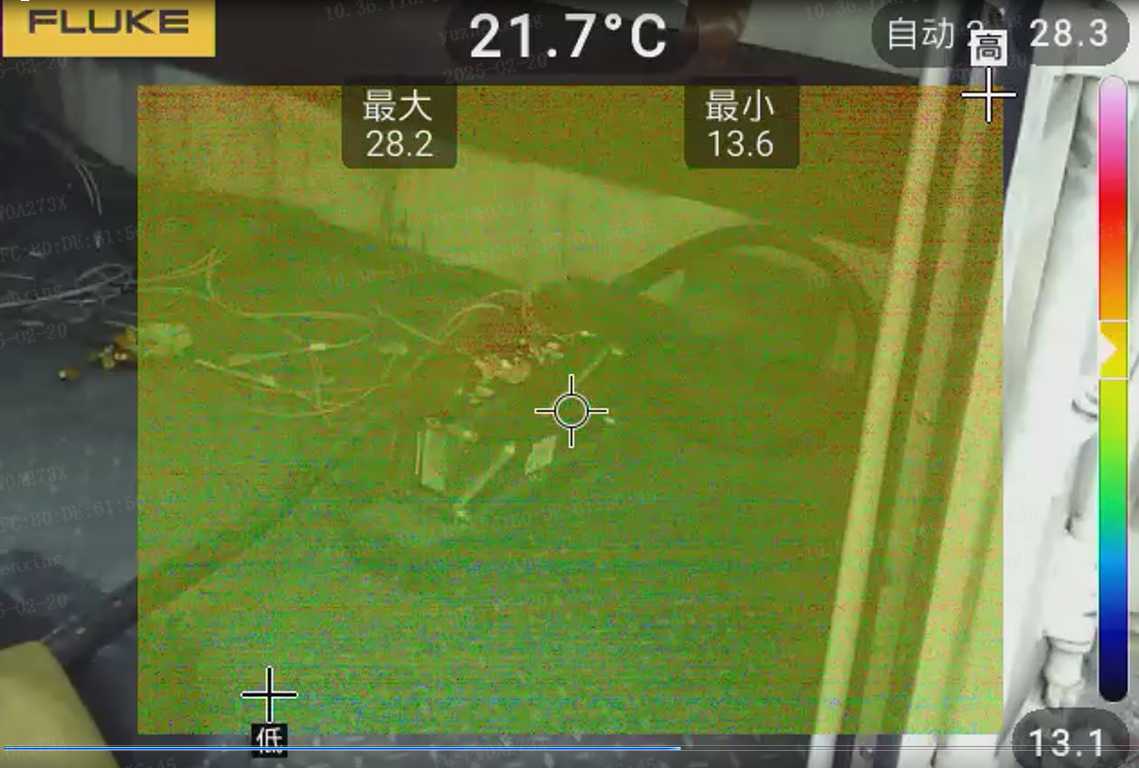

Supplement: Supplementary file 14 — Supplementary Data 1 [file 44172_2026_657_MOESM14_ESM.zip › 4.6mm-76.4V-Infrared Image.tif]

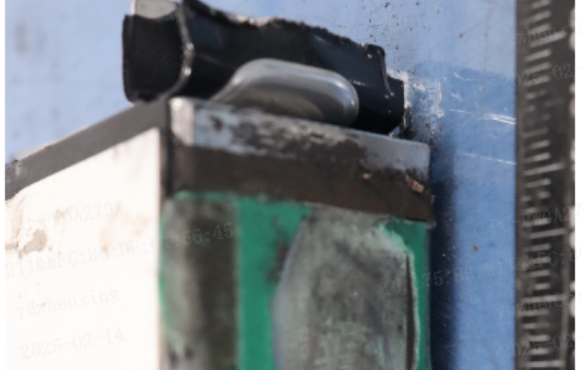

Supplement: Supplementary file 14 — Supplementary Data 1 [file 44172_2026_657_MOESM14_ESM.zip › 4.6mm-76.4V-Cells photo after test.tif]

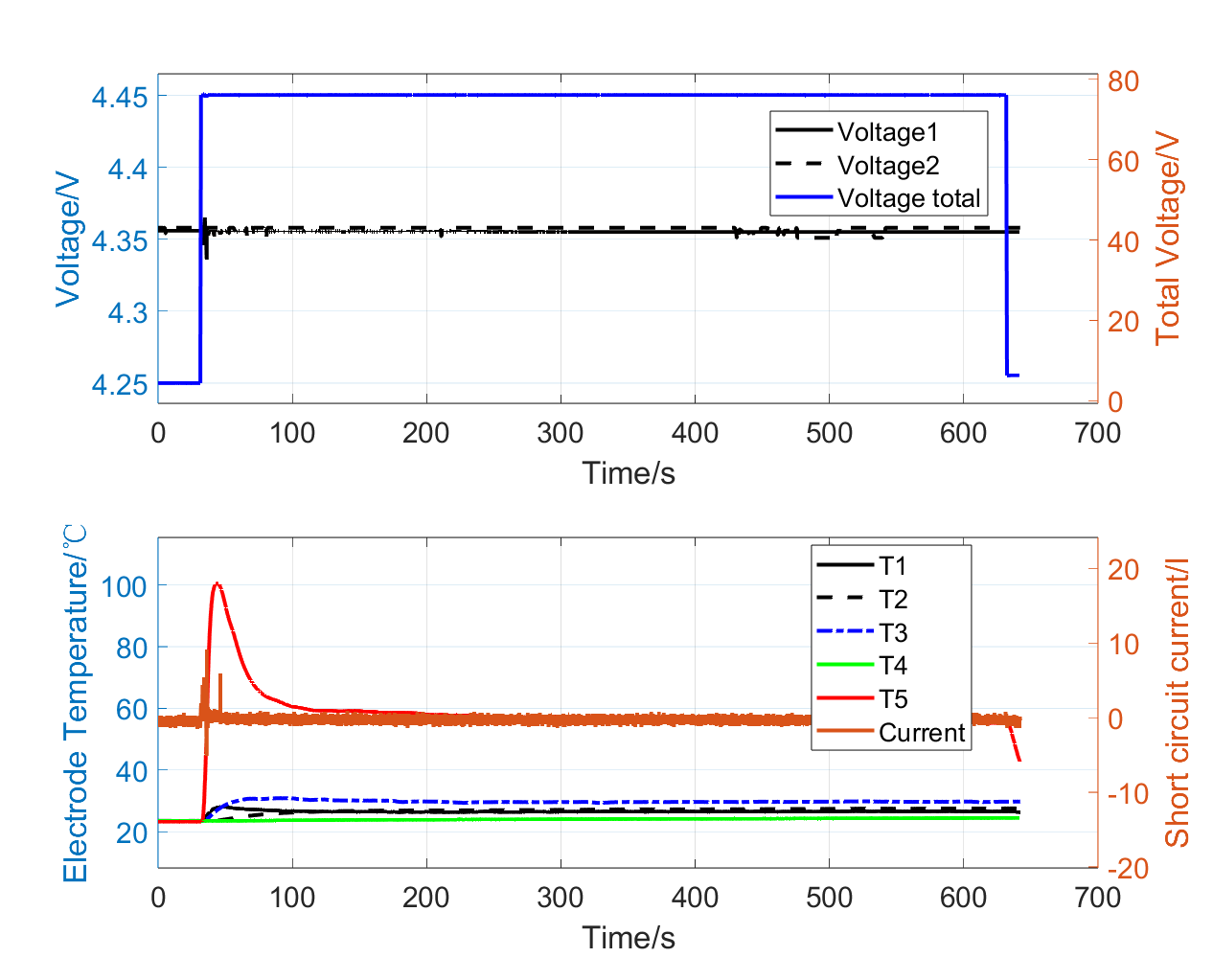

Supplement: Supplementary file 14 — Supplementary Data 1 [file 44172_2026_657_MOESM14_ESM.zip › 4.6mm-71.7V-Voltage-Temperature-Current Curves.tif]

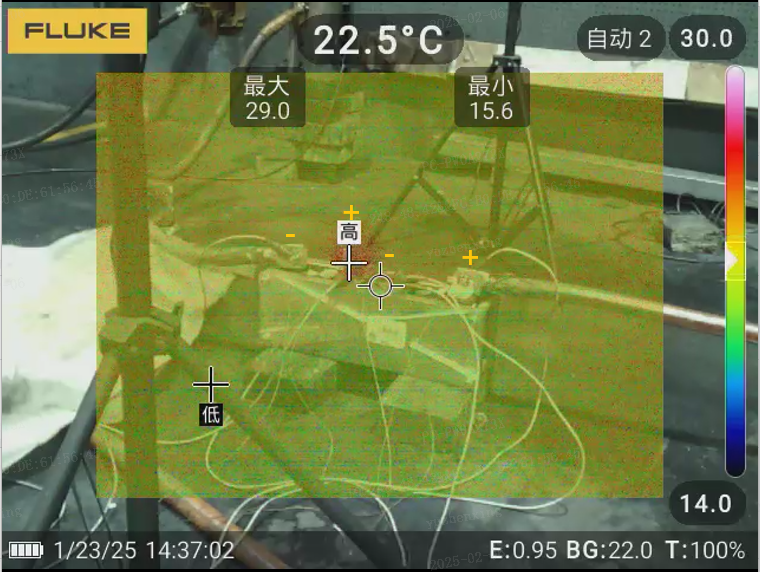

Supplement: Supplementary file 14 — Supplementary Data 1 [file 44172_2026_657_MOESM14_ESM.zip › 4.6mm-71.7V-Infrared Image.tif]

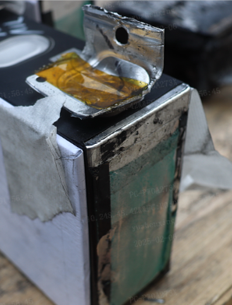

Supplement: Supplementary file 14 — Supplementary Data 1 [file 44172_2026_657_MOESM14_ESM.zip › 4.6mm-71.7V-Cells photo after test.tif]

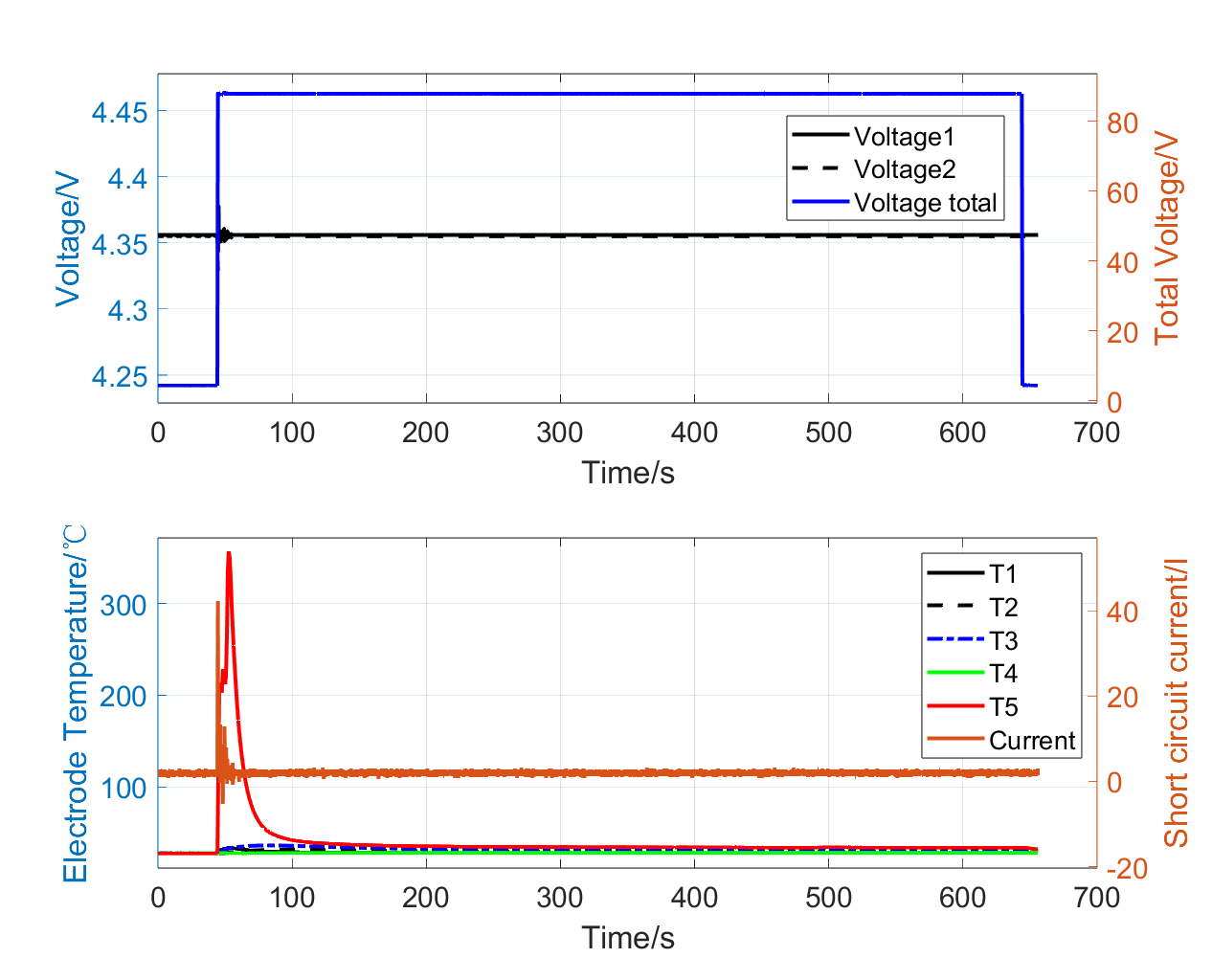

Supplement: Supplementary file 14 — Supplementary Data 1 [file 44172_2026_657_MOESM14_ESM.zip › 3.4mm-83.6V-Voltage-Temperature-Current Curves.tif]

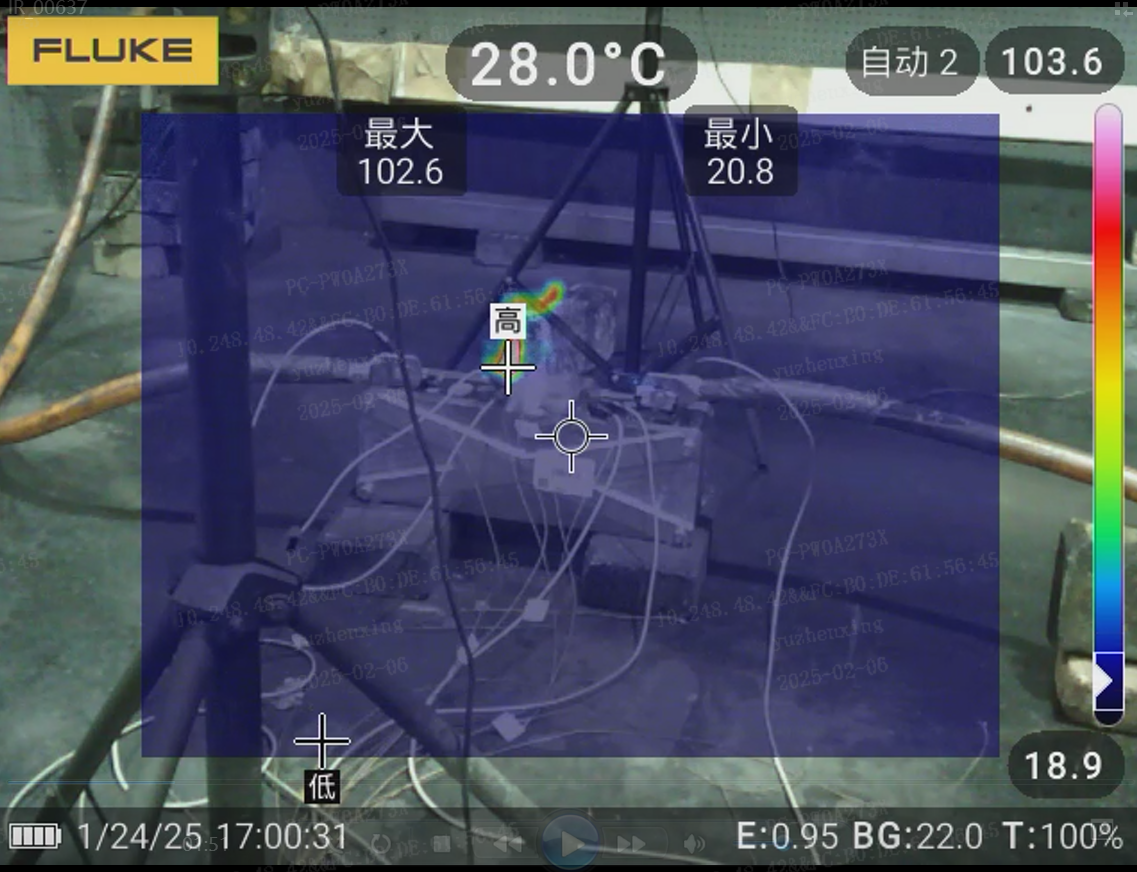

Supplement: Supplementary file 14 — Supplementary Data 1 [file 44172_2026_657_MOESM14_ESM.zip › 3.4mm-83.6V-Infrared Image.tif]

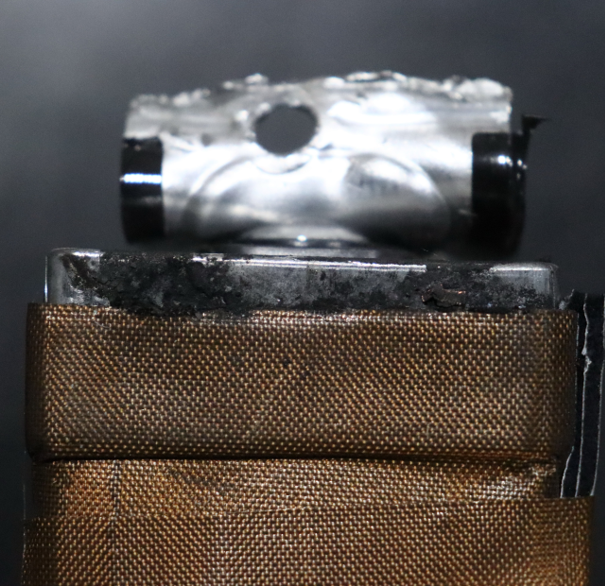

Supplement: Supplementary file 14 — Supplementary Data 1 [file 44172_2026_657_MOESM14_ESM.zip › 3.4mm-83.6V-Cells photo after test.tif]

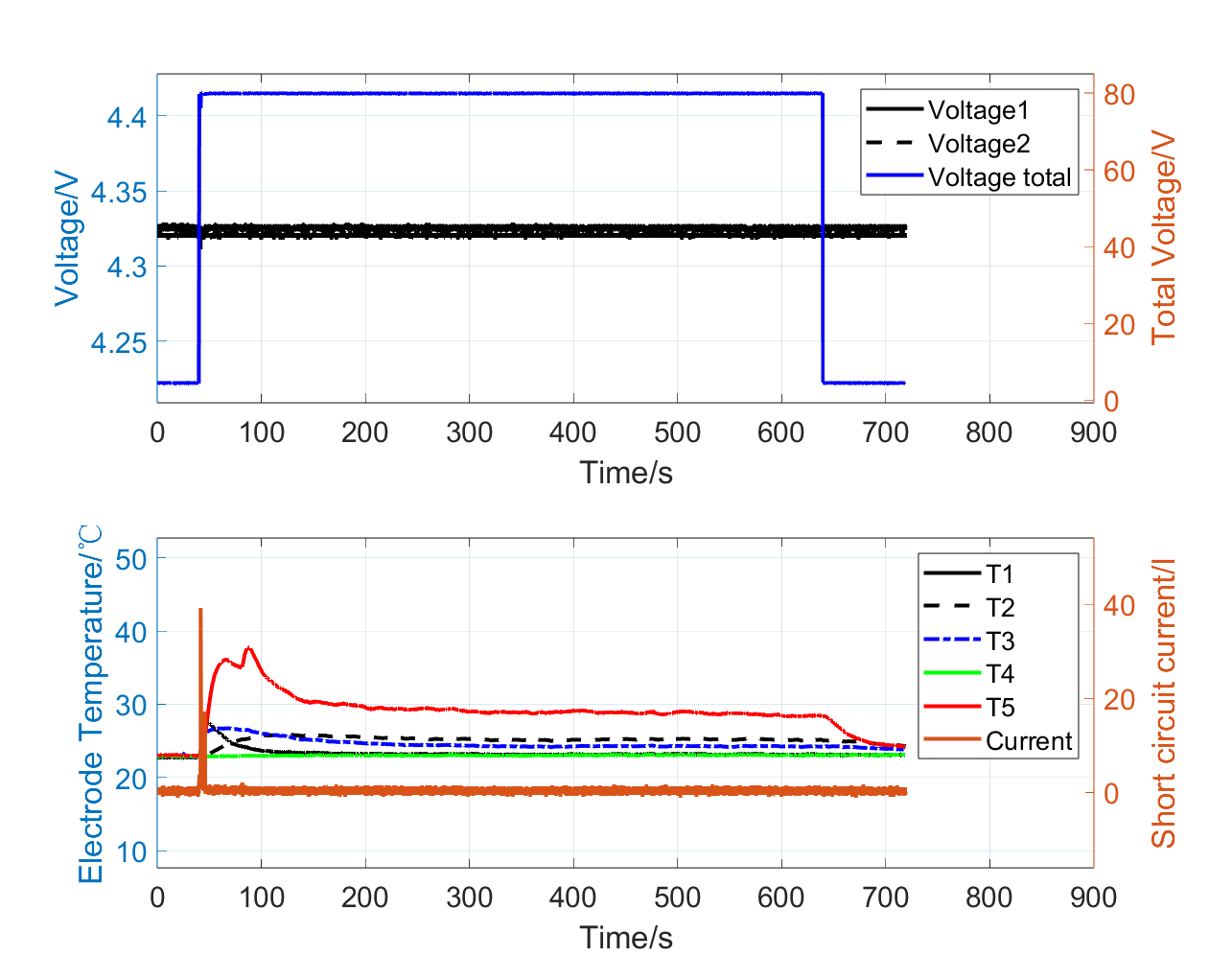

Supplement: Supplementary file 14 — Supplementary Data 1 [file 44172_2026_657_MOESM14_ESM.zip › 3.4mm-76.4V-Voltage-Temperature-Current Curves.tif]

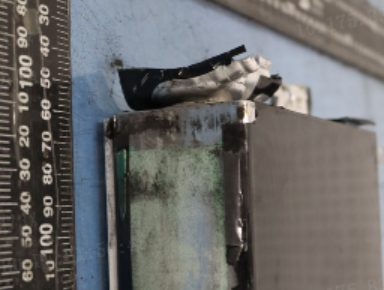

Supplement: Supplementary file 14 — Supplementary Data 1 [file 44172_2026_657_MOESM14_ESM.zip › 3.4mm-76.4V-Cells photo after test.tif]

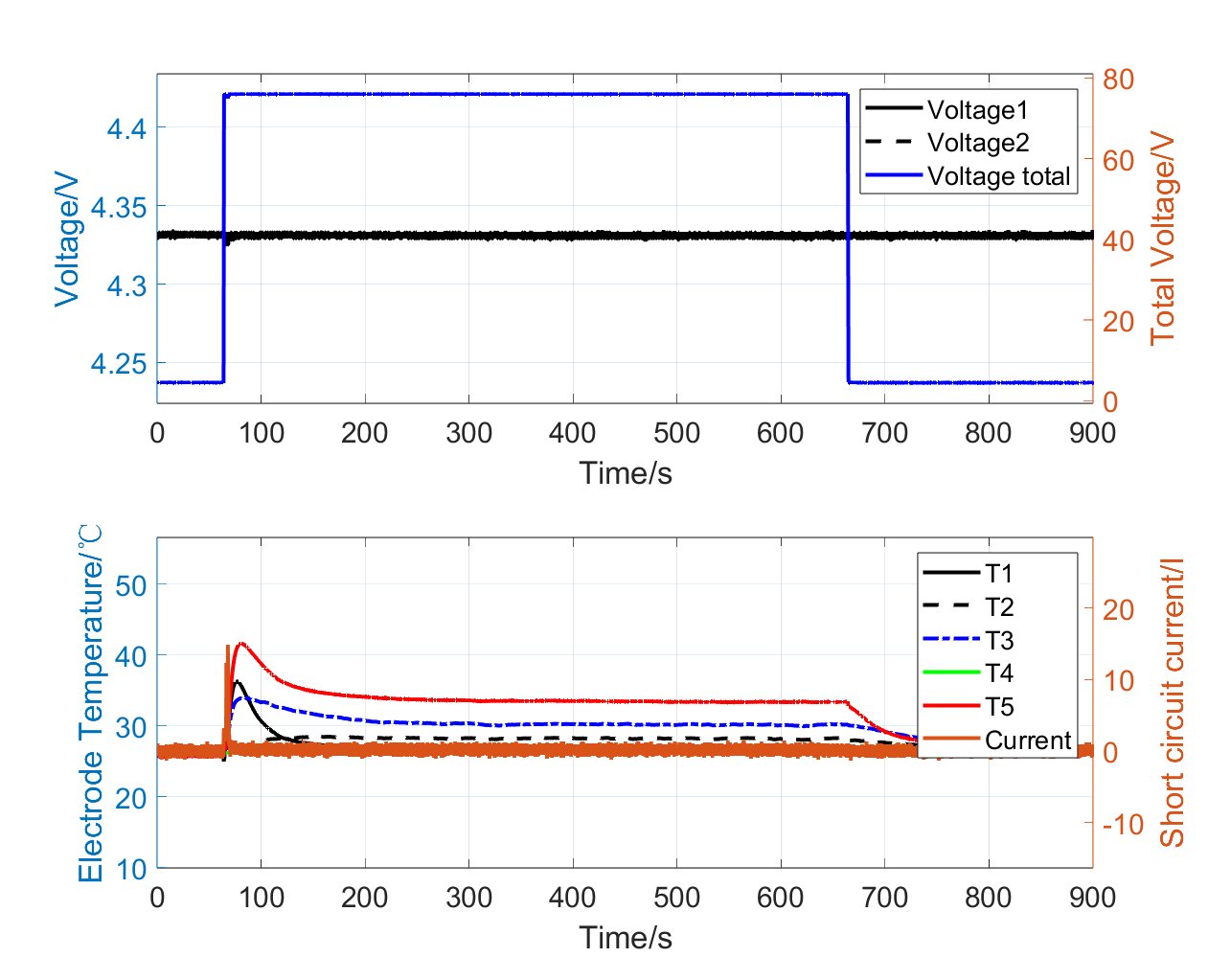

Supplement: Supplementary file 14 — Supplementary Data 1 [file 44172_2026_657_MOESM14_ESM.zip › 3.4mm-71.7V-Voltage-Temperature-Current Curves.tif]

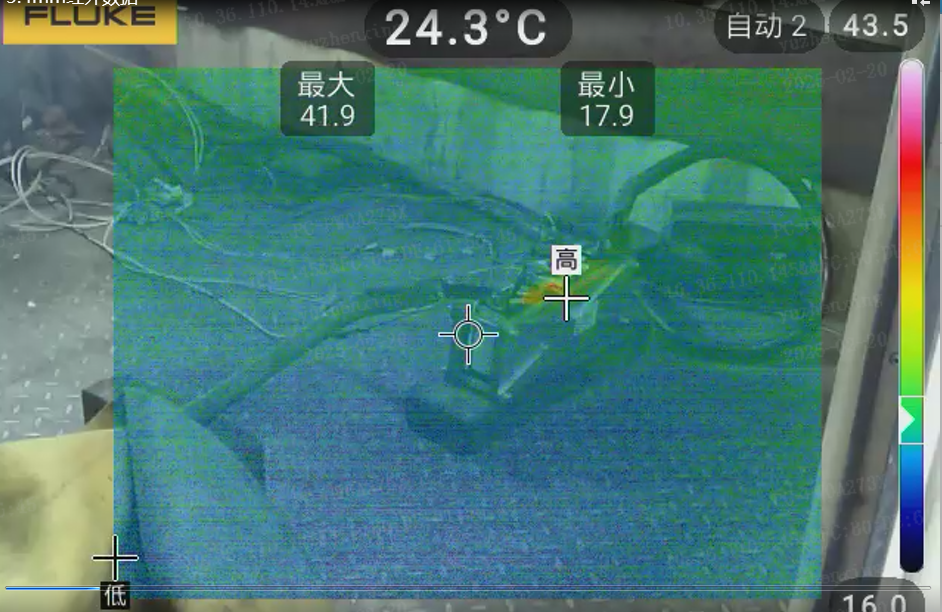

Supplement: Supplementary file 14 — Supplementary Data 1 [file 44172_2026_657_MOESM14_ESM.zip › 3.4mm-71.7V-Infrared Image.tif]

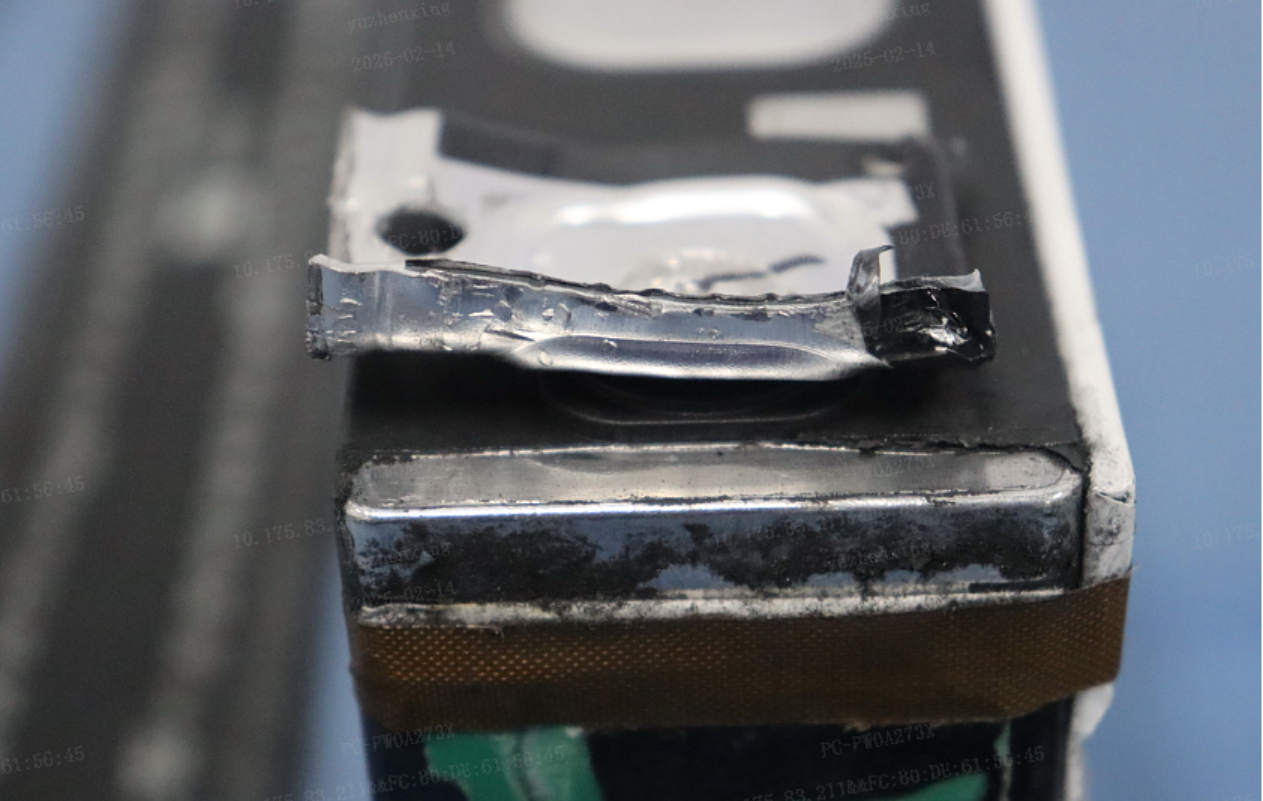

Supplement: Supplementary file 14 — Supplementary Data 1 [file 44172_2026_657_MOESM14_ESM.zip › 3.4mm-71.7V-Cells photo after test.tif]

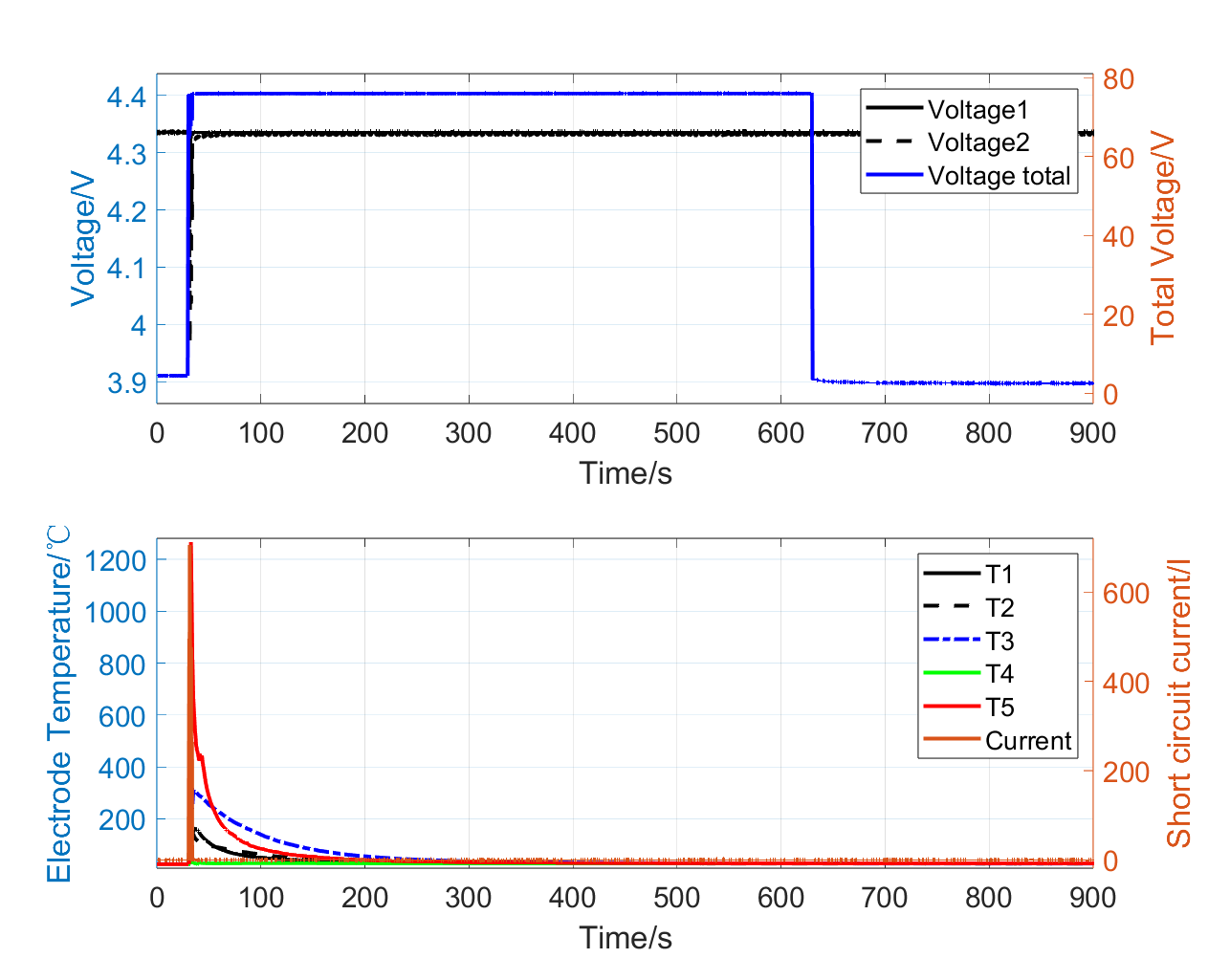

Supplement: Supplementary file 14 — Supplementary Data 1 [file 44172_2026_657_MOESM14_ESM.zip › 2.0mm-71.7V-Voltage-Temperature-Current Curves.tif]

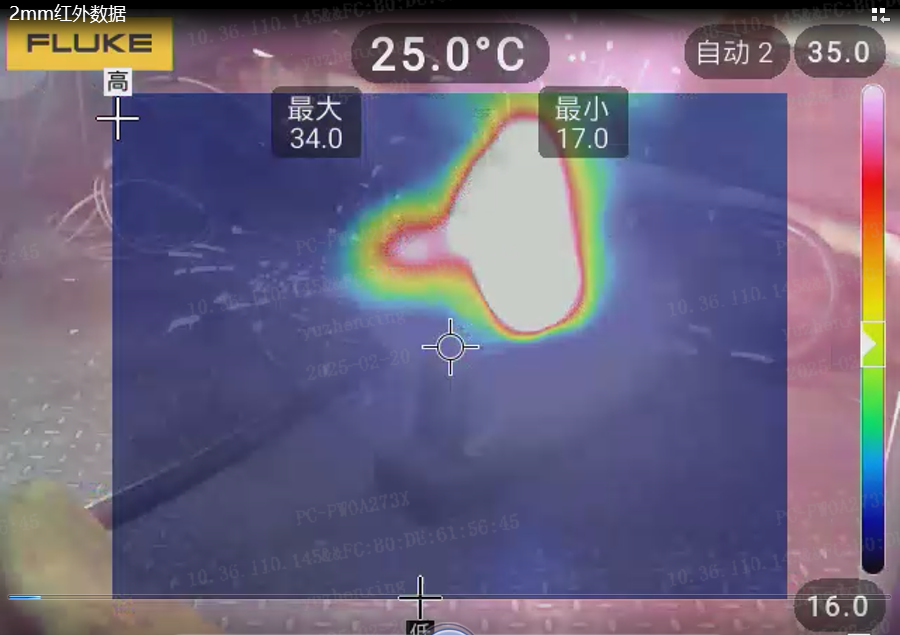

Supplement: Supplementary file 14 — Supplementary Data 1 [file 44172_2026_657_MOESM14_ESM.zip › 2.0mm-71.7V-Infrared Image.tif]

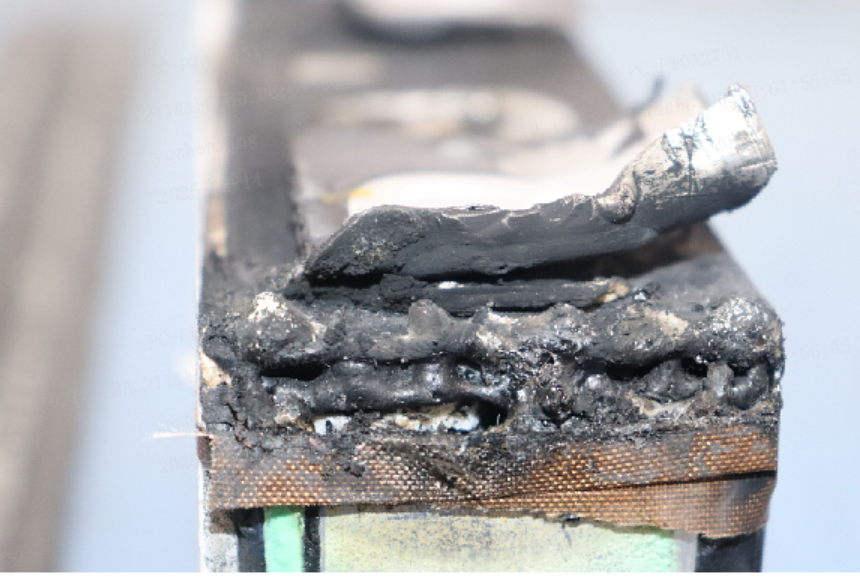

Supplement: Supplementary file 14 — Supplementary Data 1 [file 44172_2026_657_MOESM14_ESM.zip › 2.0mm-71.7V-Cells photo after test.tif]

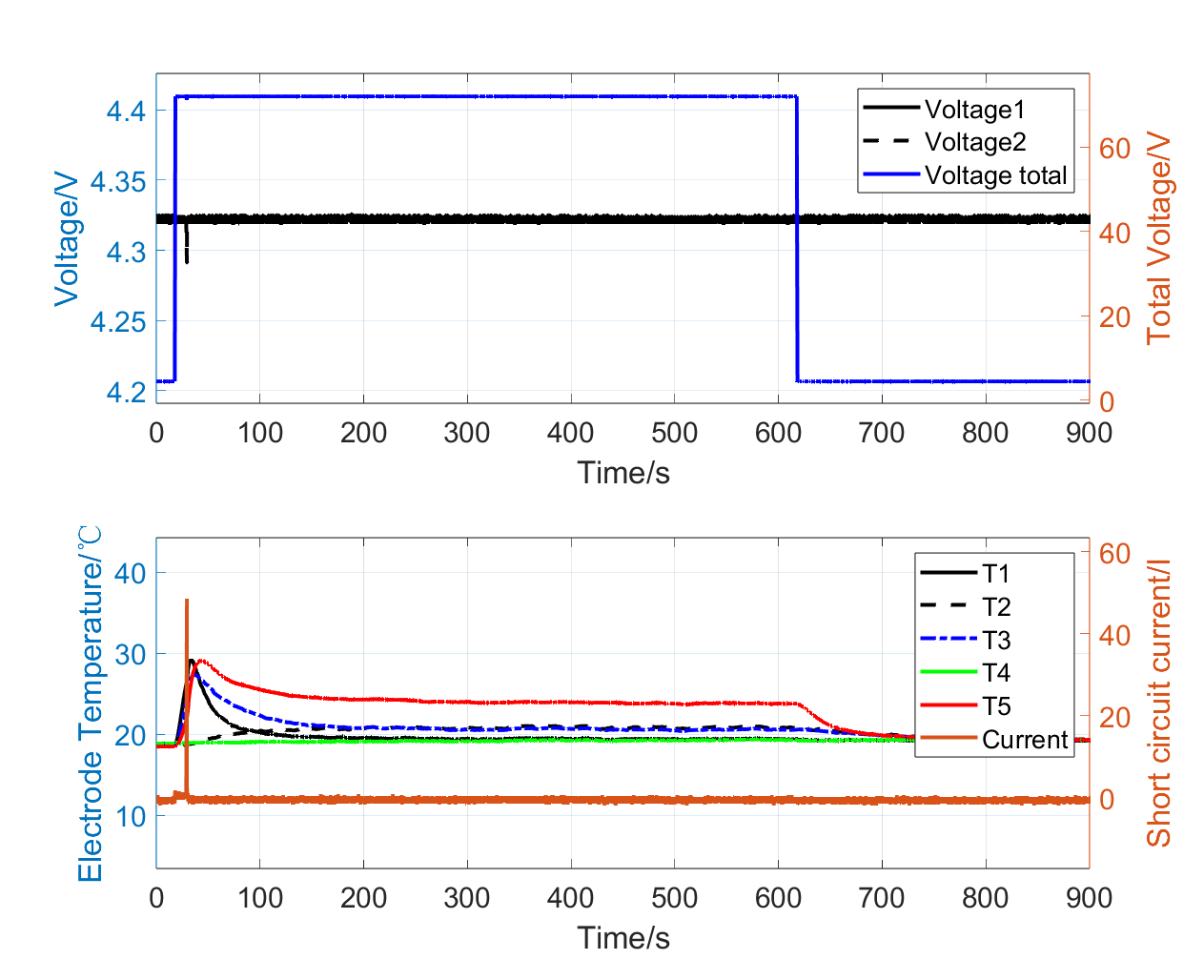

Supplement: Supplementary file 14 — Supplementary Data 1 [file 44172_2026_657_MOESM14_ESM.zip › 2.0mm-68V-Voltage-Temperature-Current Curves.tif]

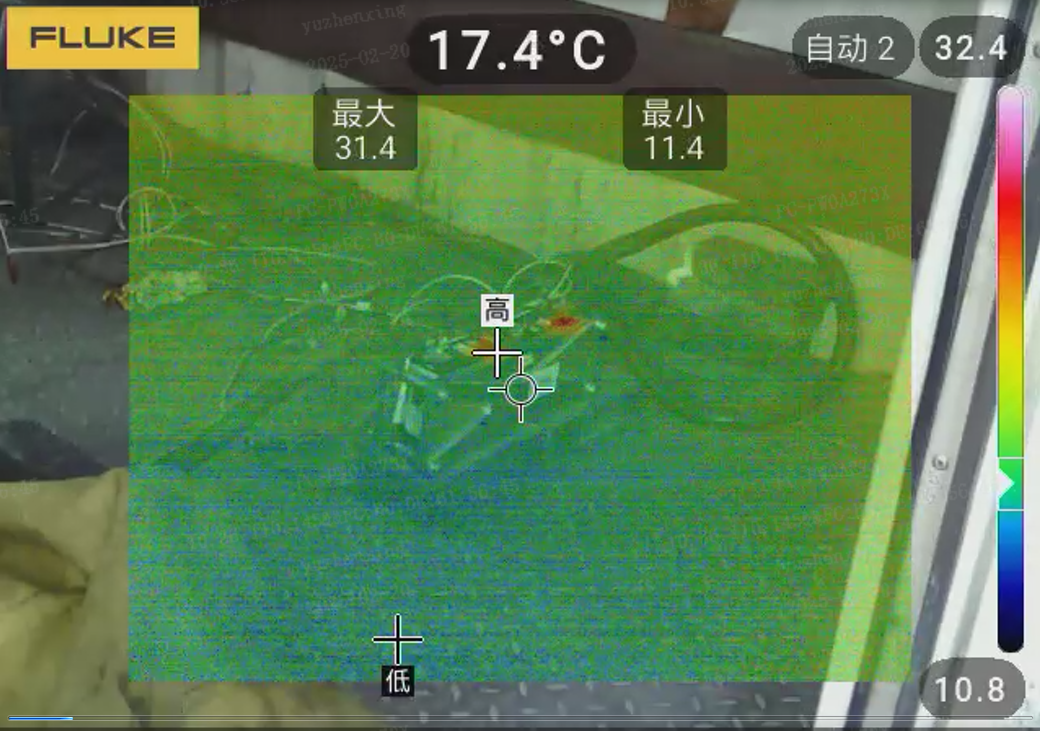

Supplement: Supplementary file 14 — Supplementary Data 1 [file 44172_2026_657_MOESM14_ESM.zip › 2.0mm-68V-Infrared Image.tif]

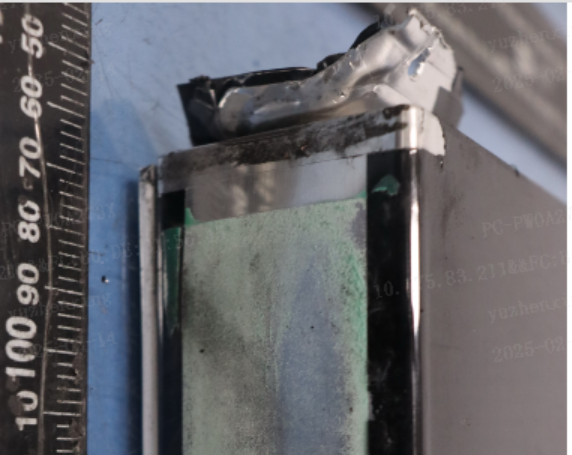

Supplement: Supplementary file 14 — Supplementary Data 1 [file 44172_2026_657_MOESM14_ESM.zip › 2.0mm-68V-Cells photo after test.tif]

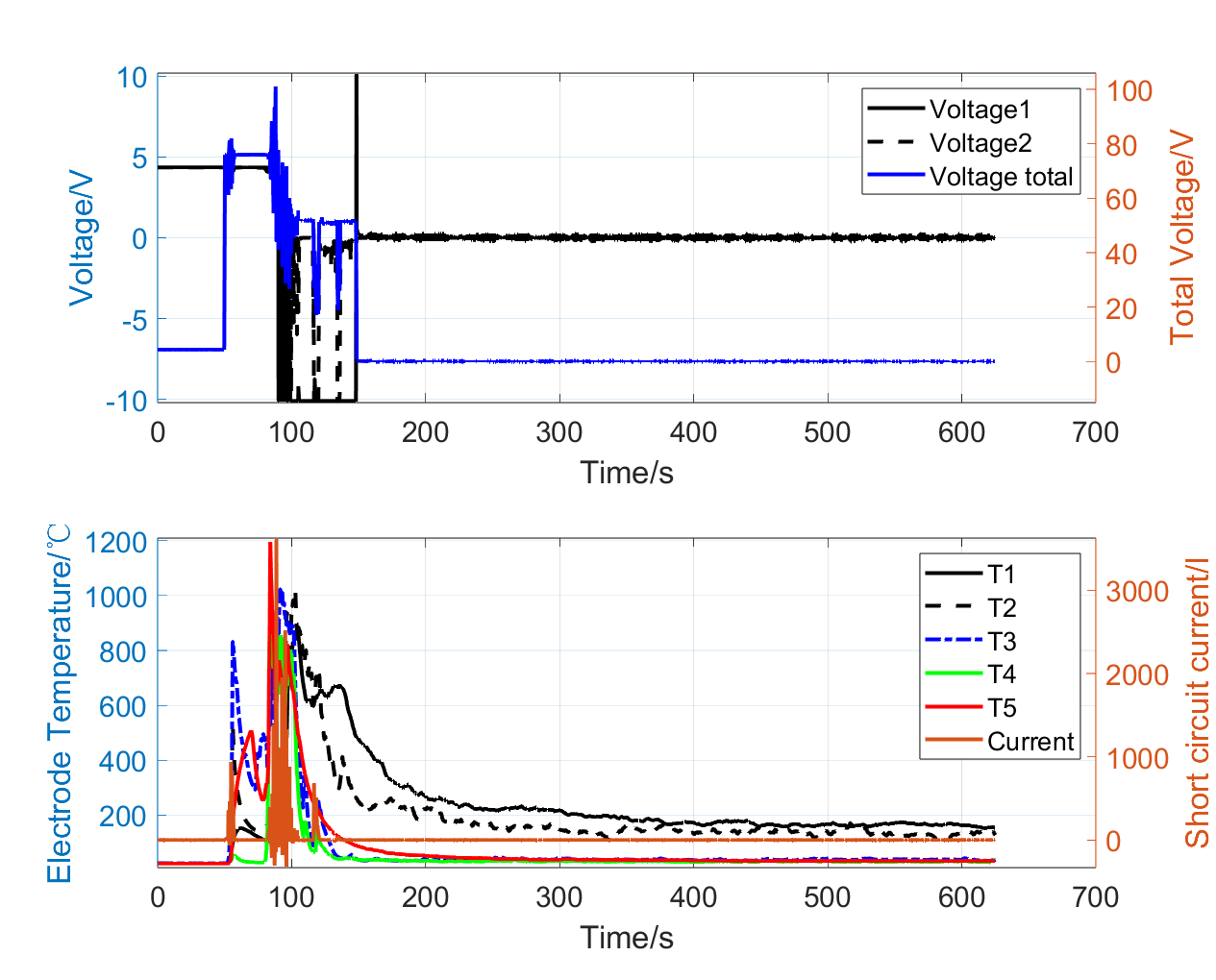

Supplement: Supplementary file 14 — Supplementary Data 1 [file 44172_2026_657_MOESM14_ESM.zip › 1.2mm-71.7V-Voltage-Temperature-Current Curves.tif]

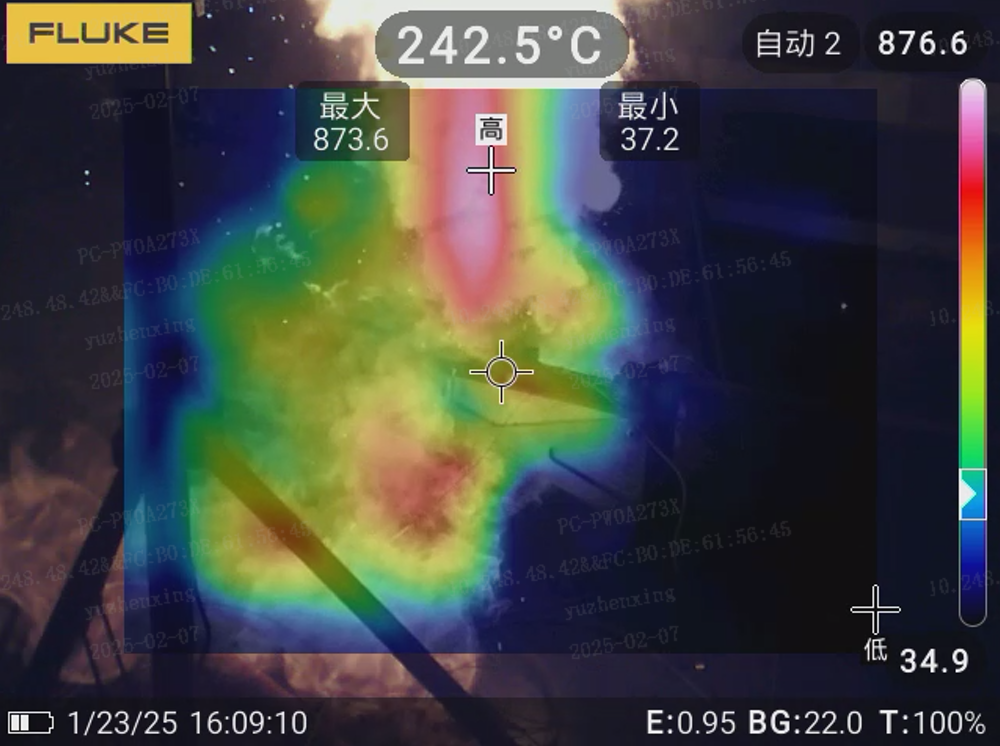

Supplement: Supplementary file 14 — Supplementary Data 1 [file 44172_2026_657_MOESM14_ESM.zip › 1.2mm-71.7V-Infrared Image.tif]

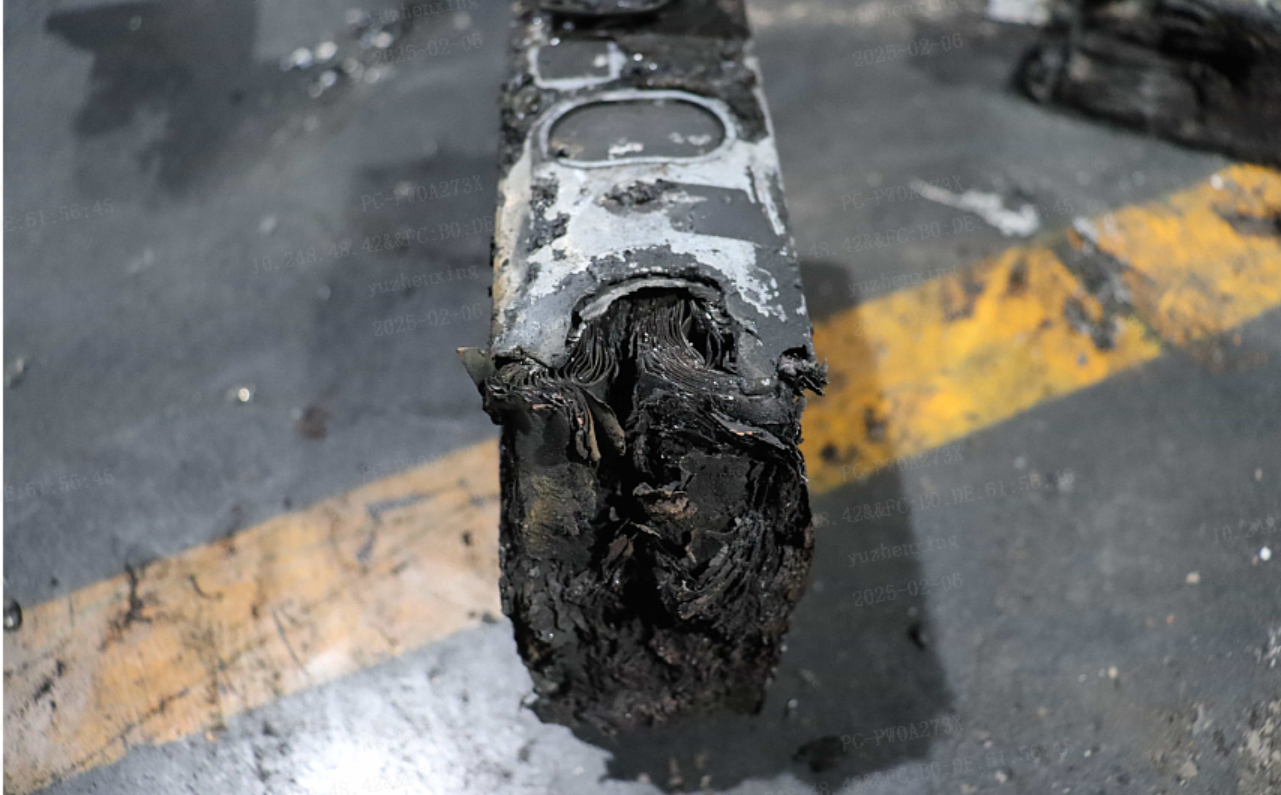

Supplement: Supplementary file 14 — Supplementary Data 1 [file 44172_2026_657_MOESM14_ESM.zip › 1.2mm-71.7V-Cells photo after test.tif]

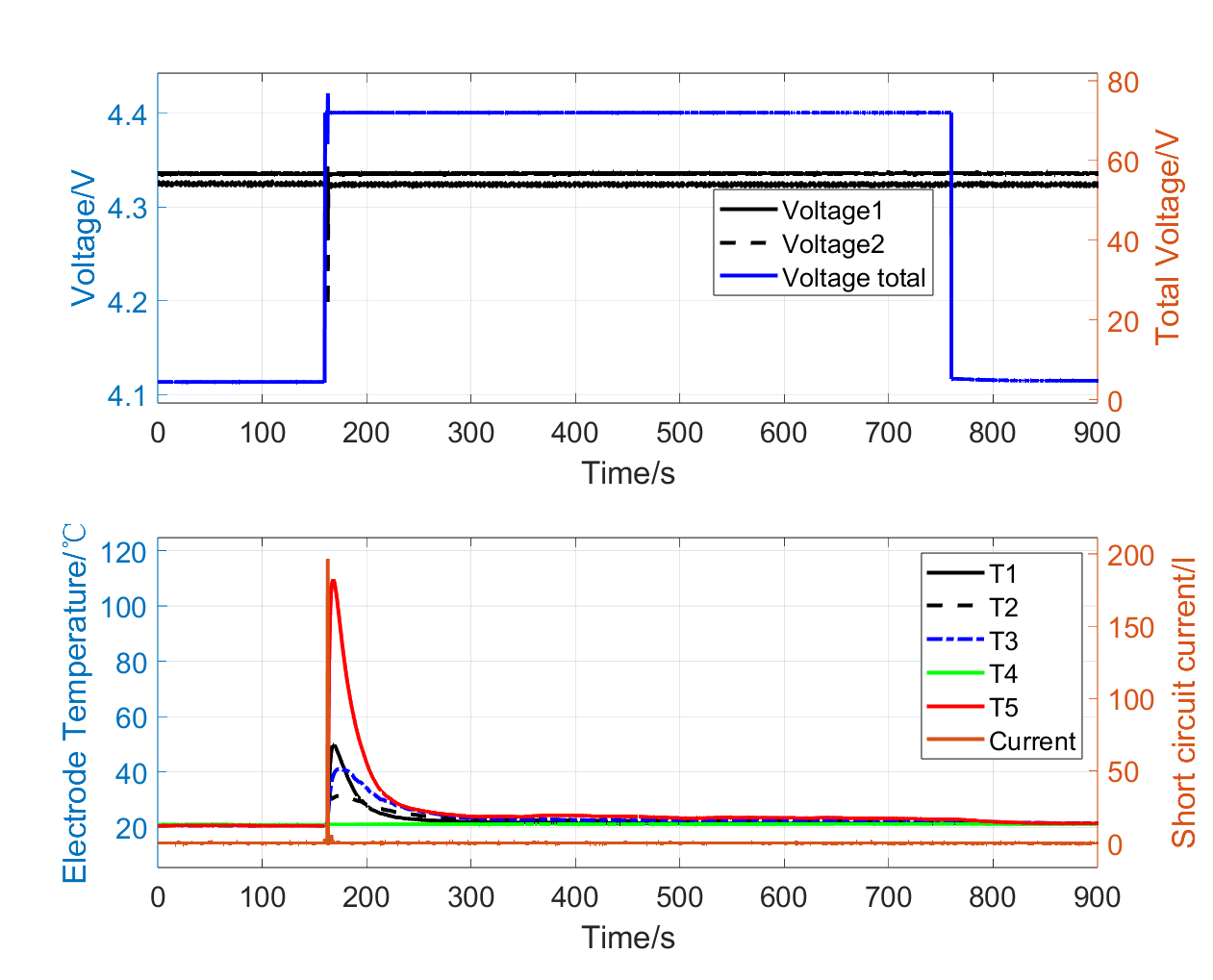

Supplement: Supplementary file 14 — Supplementary Data 1 [file 44172_2026_657_MOESM14_ESM.zip › 1.2mm-68V-Voltage-Temperature-Current Curves.tif]

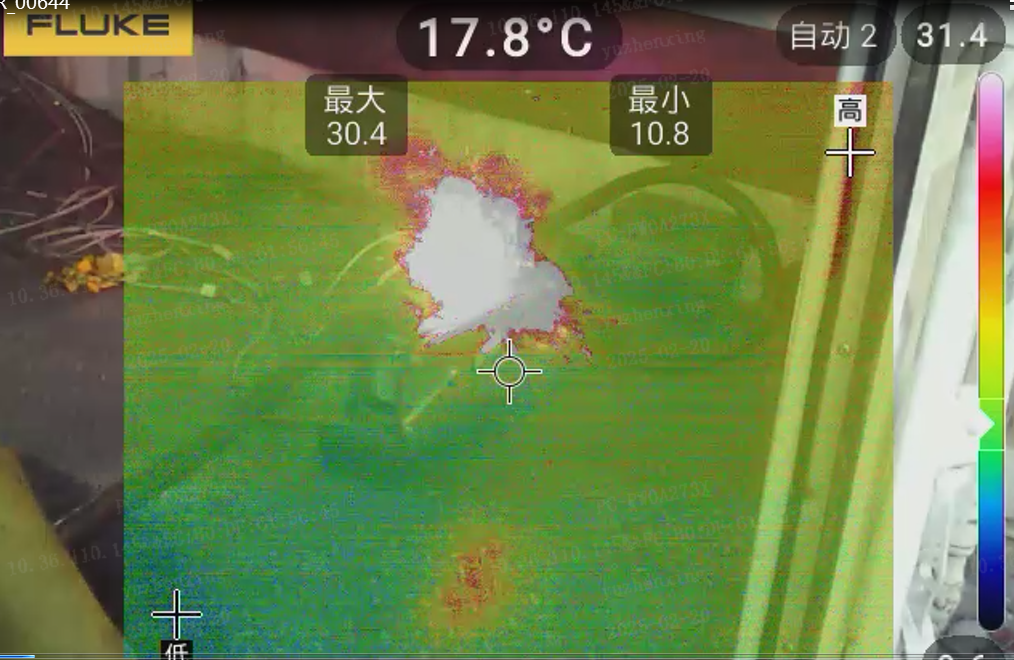

Supplement: Supplementary file 14 — Supplementary Data 1 [file 44172_2026_657_MOESM14_ESM.zip › 1.2mm-68V-Infrared Image.tif]

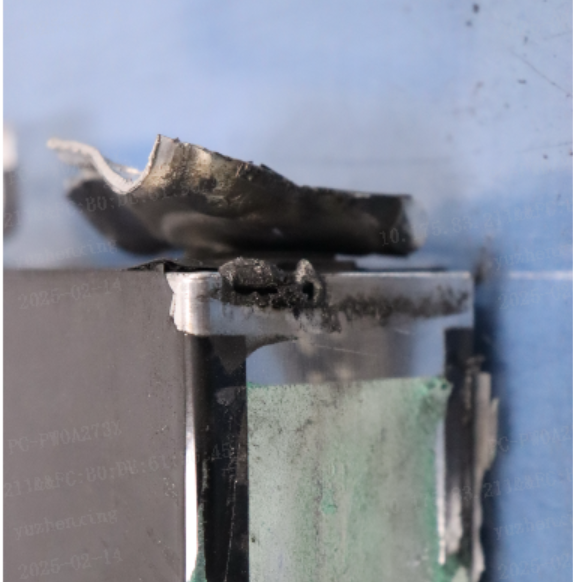

Supplement: Supplementary file 14 — Supplementary Data 1 [file 44172_2026_657_MOESM14_ESM.zip › 1.2mm-68V-Cells photo after test.tif]

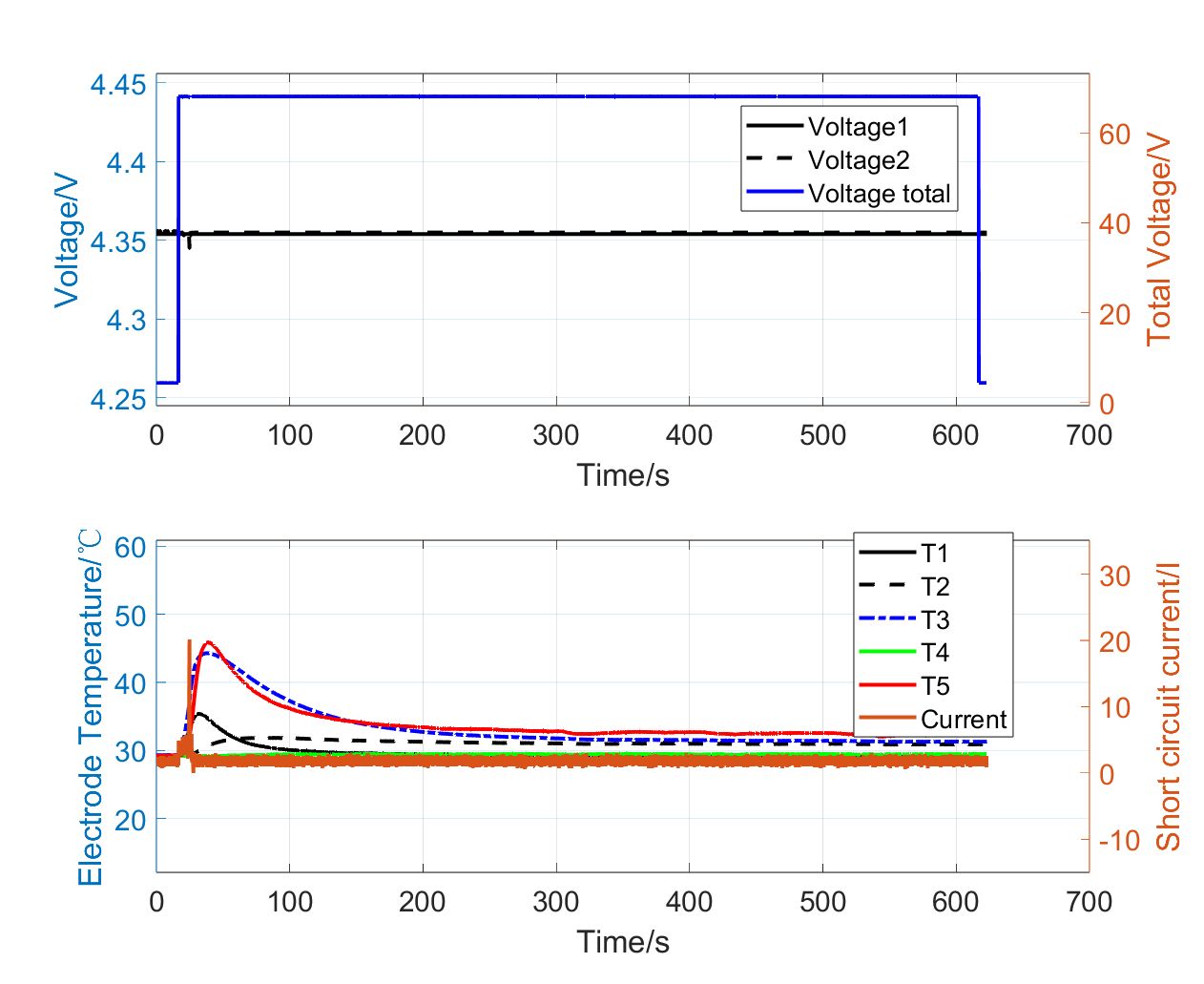

Supplement: Supplementary file 14 — Supplementary Data 1 [file 44172_2026_657_MOESM14_ESM.zip › 1.2mm-63.7V-Voltage-Temperature-Current Curves.tif]

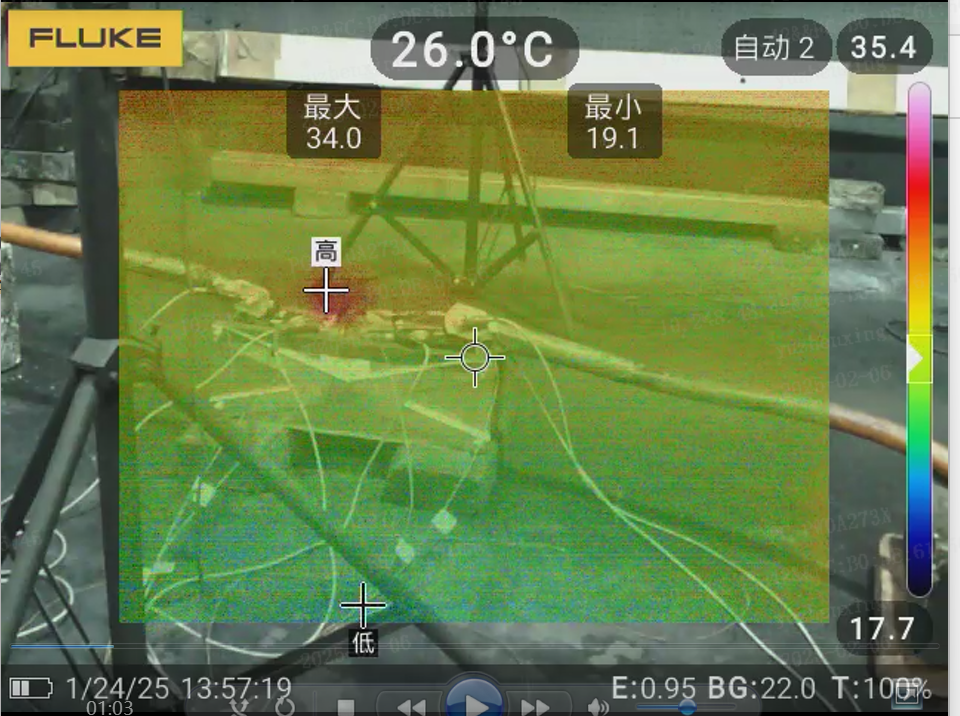

Supplement: Supplementary file 14 — Supplementary Data 1 [file 44172_2026_657_MOESM14_ESM.zip › 1.2mm-63.7V-Infrared Image.tif]

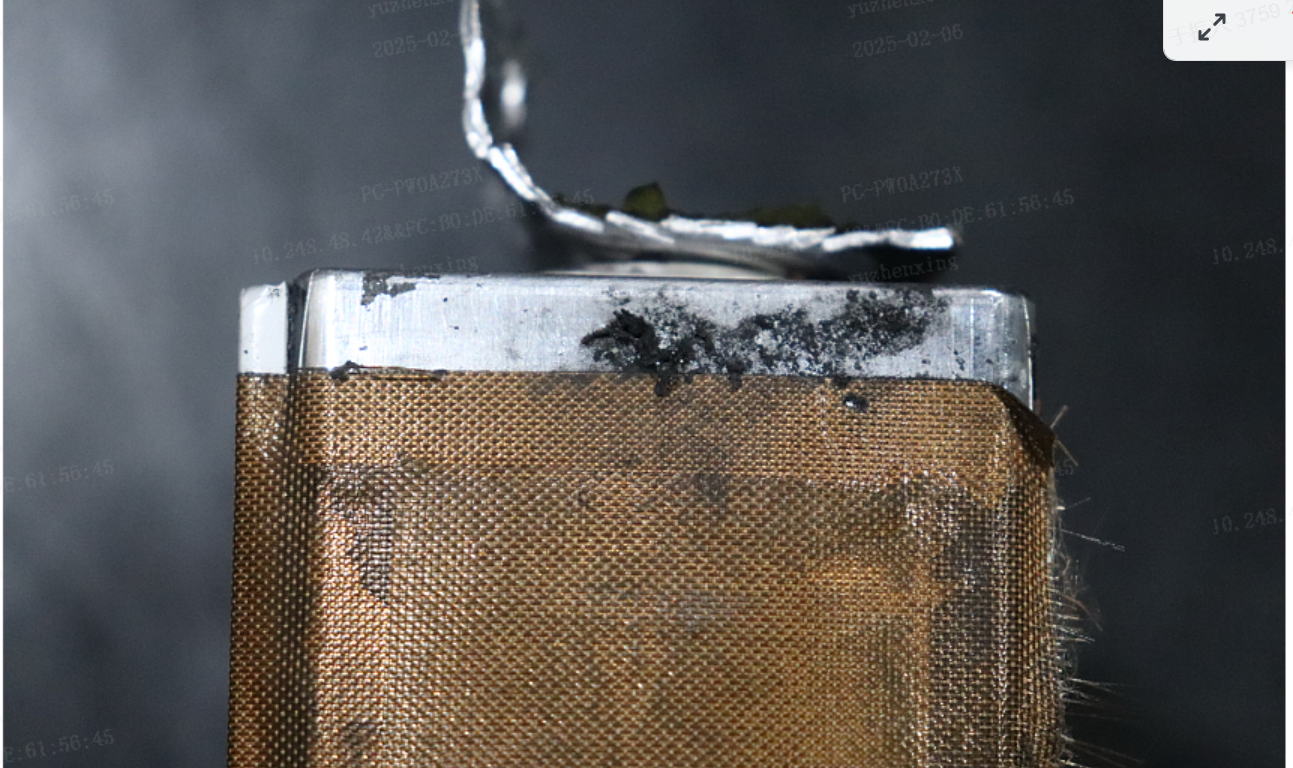

Supplement: Supplementary file 14 — Supplementary Data 1 [file 44172_2026_657_MOESM14_ESM.zip › 1.2mm-63.7V-Cells photo after test.tif]

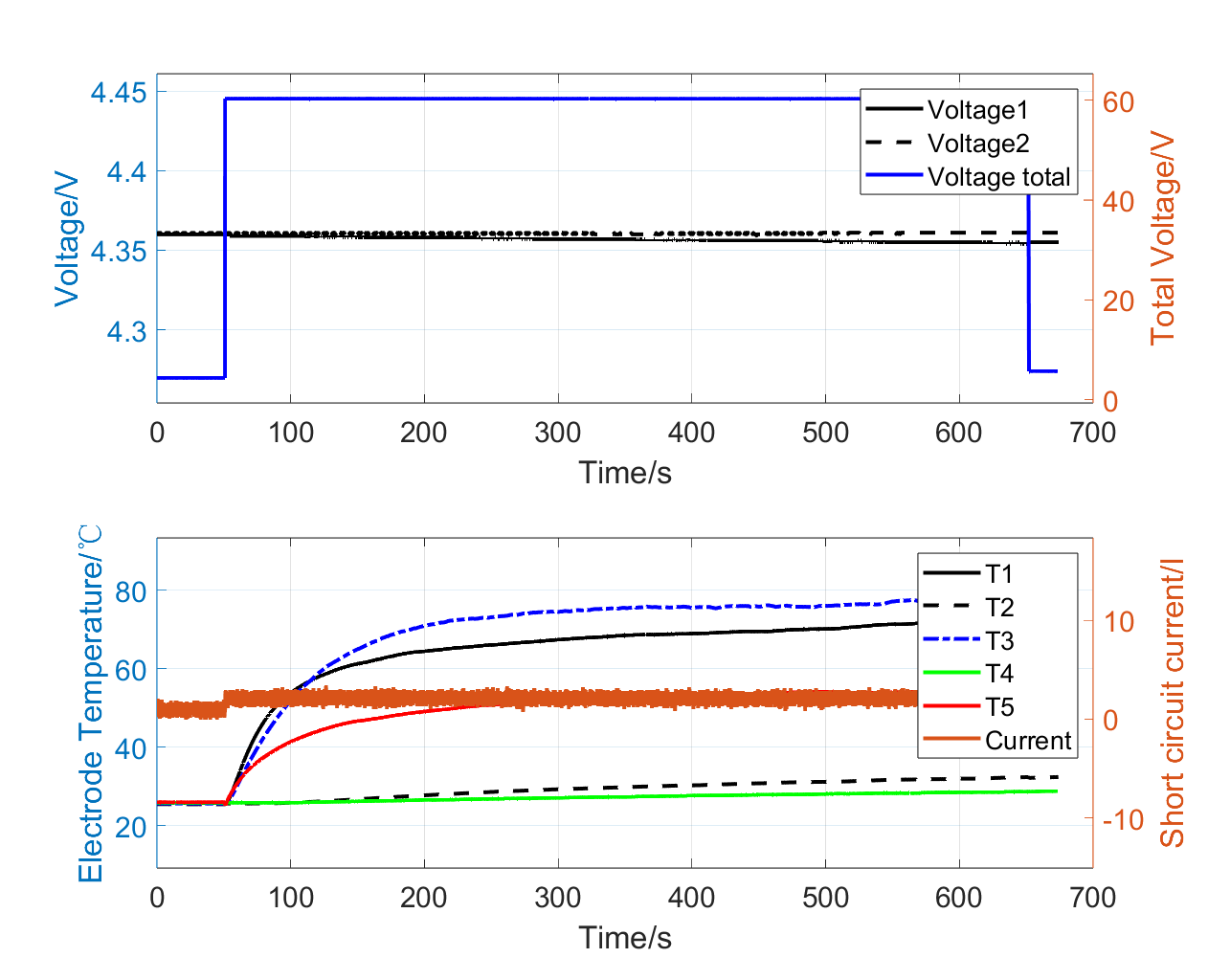

Supplement: Supplementary file 14 — Supplementary Data 1 [file 44172_2026_657_MOESM14_ESM.zip › 1.2mm-55.8V-Voltage-Temperature-Current Curves.tif]

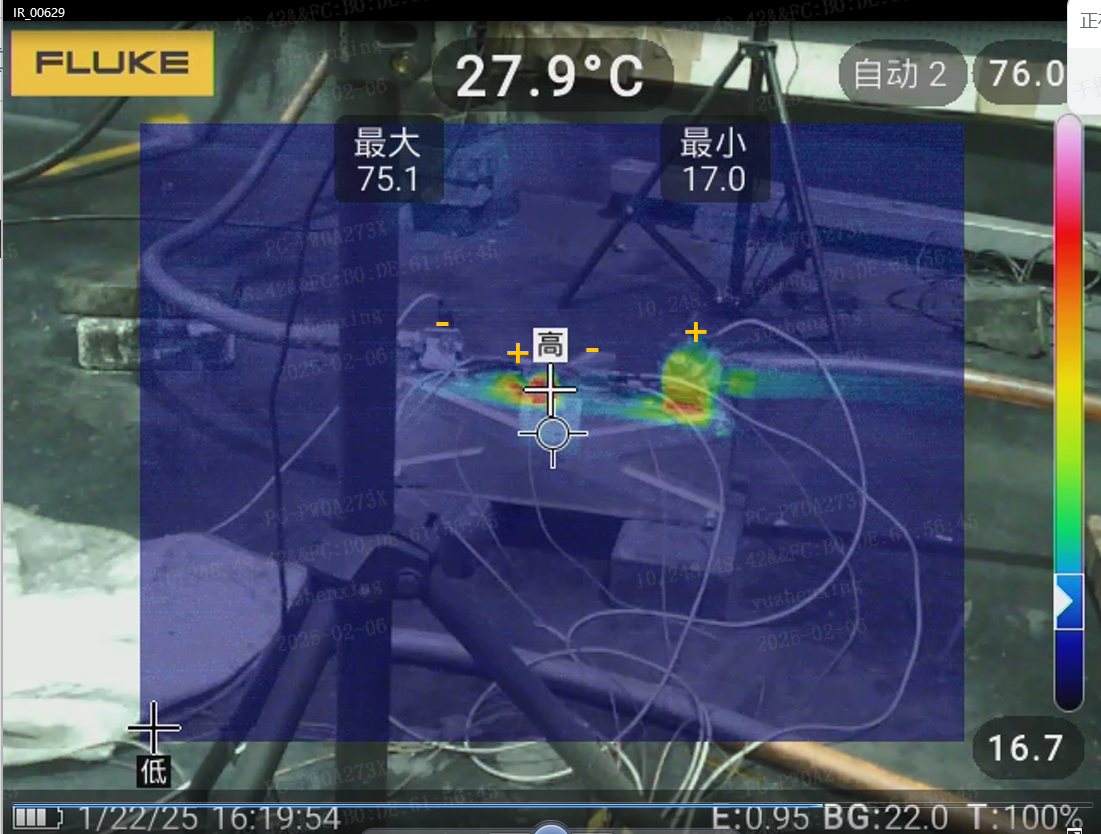

Supplement: Supplementary file 14 — Supplementary Data 1 [file 44172_2026_657_MOESM14_ESM.zip › 1.2mm-55.8V-Infrared Image.tif]

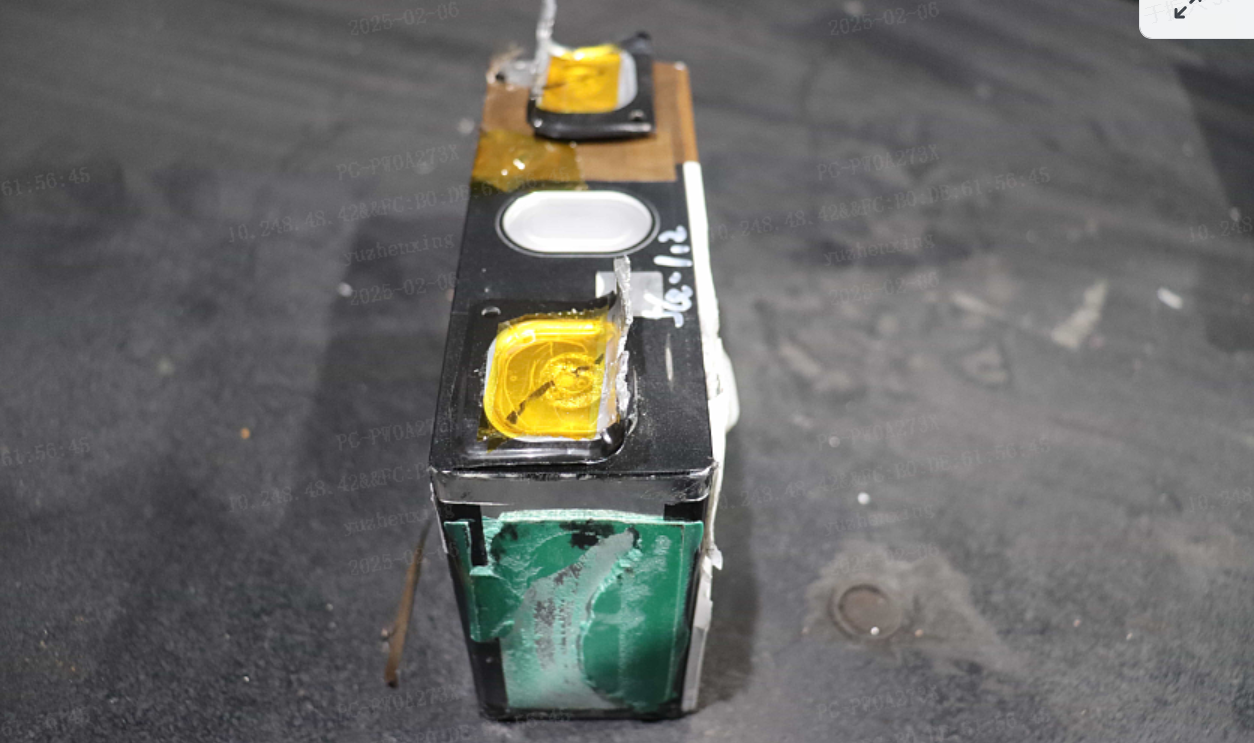

Supplement: Supplementary file 14 — Supplementary Data 1 [file 44172_2026_657_MOESM14_ESM.zip › 1.2mm-55.8V-Cells photo after test.tif]
